# Assessing the Impact of Yield Plasticity on Hybrid Performance in Maize

**DOI:** 10.1111/ppl.70278

**Published:** 2025-05-27

**Authors:** Jensina M. Davis, Lisa M. Coffey, Jonathan Turkus, Lina López‐Corona, Kyle Linders, Chidanand Ullagaddi, Dipak K. Santra, Patrick S. Schnable, James C. Schnable

**Affiliations:** ^1^ Quantitative Life Sciences Initiative, University of Nebraska‐Lincoln Lincoln Nebraska USA; ^2^ Center for Plant Science Innovation, University of Nebraska‐Lincoln Lincoln Nebraska USA; ^3^ Department of Agronomy and Horticulture University of Nebraska‐Lincoln Lincoln Nebraska USA; ^4^ Complex Biosystems Graduate Program, University of Nebraska‐Lincoln Lincoln Nebraska USA; ^5^ Department of Agronomy Iowa State University Ames Iowa USA; ^6^ US Department of Agriculture Agricultural Research Service Plant Science Research Unit Raleigh North Carolina USA; ^7^ Panhandle Research and Extension Center, University of Nebraska‐Lincoln Scottsbluff Nebraska USA

**Keywords:** agricultural crops, gene–environment interaction, nitrogen, physiological resilience, water, *Zea mays*

## Abstract

Improving crop resilience in the face of increasingly extreme and unpredictable weather and reduced access to agricultural inputs such as nitrogen fertilizer and water will require an improved understanding of phenotypic plasticity in crops. To understand the roles of different component traits in determining overall plasticity for grain yield, we generated data from a panel of 122 maize ( *Zea mays* ) hybrids grown in replicated field trials in 34 environments spanning 1126 km (700 miles) of the US Corn Belt. We observed that the levels of genetic versus environmental control and the relationships between mean parent release year, overall performance, and linear plasticity were trait‐dependent across the 18 agronomic and yield components studied. Importantly and unexpectedly, we observed no clear tradeoff between linear plasticity and mean performance and found only rare examples where genotype‐by‐environment interactions would alter selection decisions based on the environments tested in our dataset. Furthermore, we showed that overall plasticity was repeatable and that plasticity in response to nitrogen fertilization was not, which may help explain the limited success in breeding for nitrogen use efficiency. Together, these findings improve our understanding of phenotypic plasticity, with implications for maize breeding.

## 1 Introduction

Phenotypic plasticity is the ability of genetically identical individuals to exhibit different phenotypes in response to different environmental conditions. Differences in phenotypic response to the same environments between different genotypes are referred to as genotype‐by‐environment interactions (Bernardo 2020; Kusmec et al. 2018). The proportion of total phenotypic variance attributed to genotype‐by‐environment interactions varies substantially depending on the population being tested, the environments in which data are collected, and the trait(s) being studied. Phenotypic plasticity can be modelled either as changes in phenotype due to differences in environments, treating the environments as individual factors, or as a relationship between the phenotype and quantitative environmental factors, treating the environments as values along a quantitative axis. In plant breeding, new genetically distinct cultivars have been developed and selected over a period of many years, and the final selected varieties will be grown in a wide range of environments over subsequent years. Numerous plant traits of interest to growers exhibit substantial plasticity across environments. The variability in phenotypic plasticity across varieties and traits represents a challenge for plant breeders and farmers because data on how crop varieties perform in one environment will be an imperfect predictor of how these crop varieties will perform in another environment. However, an improved understanding of phenotypic plasticity will help address this challenge by improving predictions.

Changes in climate and agronomic practices also pose challenges for plant breeders and farmers. Historically, plant breeding has occurred in a world of relatively stable environments such that data from numerous locations in past years were able to capture crop performance in a range of environments that were roughly representative of the future environments in which selected crop varieties would be grown. However, due to changes in both climate and management practices, the current environments in which new crop varieties are being evaluated are likely less representative of the future environments in which selected crop varieties will be grown. One likely change in management practices is the reduced use of synthetic nitrogen fertilizers due to economic, environmental, and political factors (Clerk of the Legislature's Unicameral Information Office 2024; Gao et al. 2024; USDA NRCS 2023). Although it is difficult, if not impossible, to predict and replicate the climatic conditions of the future in our current evaluation environments, it is straightforward to create evaluation environments that consider reduced nitrogen fertilization.

One method of summarizing the phenotypic plasticity of crop varieties is by utilizing model parameters extracted from joint regression models such as Finlay–Wilkinson regression (Finlay and Wilkinson 1963). As discussed above, it is possible to model the environment as either a factor or a quantitative explanatory variable. In principle, each environmental factor likely plays a role in determining phenotypes and different crop varieties may exhibit different responses to different environmental factors. However, given both the extremely large number of potential environmental factors that can vary between experiments and the limited number of total environments in which even the largest replicated field experiments are conducted, it can often be challenging to link variation in specific plant traits to specific environmental factors. Finlay–Wilkinson regression regresses the varietal performance for a given trait across multiple environments on the population‐level performance for the trait in a given environment. This jointly estimates the overall genotypic effect as the intercept and its relative response to improved environments as the slope. This method has been widely used to study the genetic architecture of phenotypic plasticity in plants by using the fitted slope as a metric of linear phenotypic plasticity (Kusmec et al. 2017; Liu et al. 2023; Jin et al. 2023; Li et al. 2019; Li et al. 2021).

Breeders may select for different types of yield stability, a concept related to phenotypic plasticity (Kusmec et al. 2018). Several types of stability have also been defined. Type I stability indicates constant performance across environments, and a Type I stable hybrid would be modeled in Finlay–Wilkinson regression as having a linear plasticity value of 0. Type II stable lines have a population mean level of response to improved environments and would be modeled in Finlay–Wilkinson regression as having a linear plasticity value of 1. Selection for Type I stable hybrids with high yields aims to minimize the effect of genotype‐by‐environment interactions, but unfortunately, lines with Type I stability are most often low‐performing lines. In contrast, selection for Type II stability aims to exploit some of the benefits of genotype‐by‐environment interactions while minimizing their deleterious effects. Either of these measures of stability implies selection against hybrids with high linear plasticity values because of the concern that lines with high linear plasticity values will perform poorly relative to the population in poor environments (Finlay and Wilkinson 1963; Bernardo 2020). However, contrasting evidence exists for the relationship between mean trait values and plasticity. In Finlay and Wilkinson (1963), the authors studied a wheat diversity panel and found a tradeoff between extreme (both high and low) plasticity values and overall performance in terms of yield. Other studies have found either a positive relationship (Li et al. 2019; Liu et al. 2023) or no relationship (Alvarez Prado et al. 2014) between plasticity and yield, though this may be due to differences in the particular traits studied. For example, Kusmec et al. (2017) found that the direction and strength of the relationship between trait mean value and the trait linear plasticity was trait‐dependent.

Understanding the contributions of both genotype‐by‐environment interactions and phenotypic plasticity to crop performance helps guide decision‐making in plant breeding programs today. This understanding will allow us to develop and select cultivars most likely to perform well in future environmental conditions. Both phenotypic plasticity and genotype‐by‐environment interactions have been extensively studied in plant systems (Kusmec et al. 2017; Liu et al. 2023; Jin et al. 2023; Liu et al. 2021; Li et al. 2019; Incognito et al. 2022; Alvarez Prado et al. 2014; Fu and Wang 2023; Guo et al. 2024; Vanous et al. 2019; Huang et al. 2023; Mu et al. 2022). However, the majority of these studies have examined only a few traits (Vanous et al. 2019; Incognito et al. 2022; Fu and Wang 2023; Mu et al. 2022), were limited in the range or agronomic relevance of the environments in which varieties were tested (Li et al. 2019; Vanous et al. 2019; Jin et al. 2023; Alvarez Prado et al. 2014), or used little to no commercially relevant genetic material. Of the studies examining phenotypic plasticity in maize ( *Zea mays* ), many have focused on identifying genomic regions associated with variation in phenotypic plasticity, and most used inbred or doubled‐haploid lines (Kusmec et al. (2017); Li et al. 2019; Vanous et al. 2019; Alvarez Prado et al. 2014; Fu and Wang 2023; Jin et al. 2023; Liu et al. 2023), which are not grown commercially. Studying phenotypic plasticity in a hybrid population allows for an improved understanding of phenotypic plasticity in commercially relevant germplasm, as most commercial maize production uses hybrid lines. In addition, it is also possible to estimate the contribution of parental inbred lines to the phenotype of the hybrid, termed general combining ability (GCA) (Bernardo 2020).

Part of the complexity of understanding how changes in the environment affect crop productivity is that phenotypic outcomes for complex traits such as grain yield are determined by variations in a suite of component phenotypes. Many factors affect grain yield in maize; yield component phenotypes include ears per plant, kernel rows per ear, kernels per row, and mass per kernel. Each of these traits is under partially independent genetic control, and each could, in principle, exhibit different patterns of response to changes in the environment. Several studies have investigated the phenotypic plasticity of maize yield components (Liu et al. 2021; Alvarez Prado et al. 2014; Kusmec et al. (2017); Liu et al. 2023; Li et al. 2019; Jin et al. 2023), but only one (Liu et al. 2021) used a hybrid population. In addition, how the phenotypic plasticity of maize yield components and agronomic traits differs between older and newer germplasm remains unknown.

In this work, we evaluated the performance of a set of 122 maize hybrids from different breeding eras across up to 34 unique environments representing multiple nitrogen treatments in 10 of the 14 location years for 18 agronomic and yield component traits. Using this dataset, we evaluated the relationships between phenotypic plasticity and performance across environments, with implications for maize breeding programs.

## 2 Results

**FIGURE 1:**
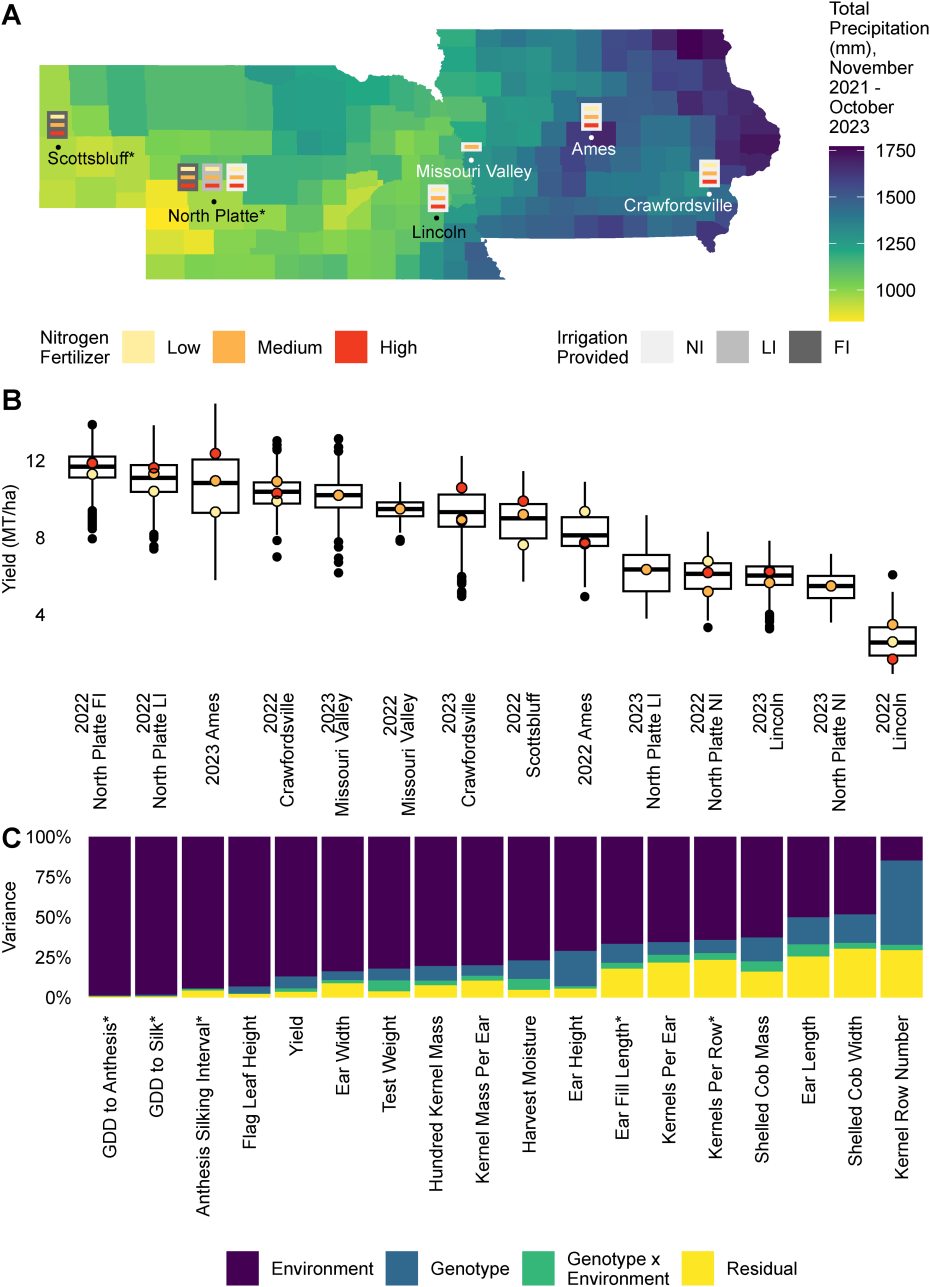
Multi‐environment hybrid maize field trials across Nebraska and Iowa show a large effect of environment on yield component traits aside from kernel row number. (A) An overview of the experimental design utilized in this study overlaid on a map of Nebraska and Iowa, showing the total precipitation during the period relevant to the growing seasons in this study (2022–2023) from the approximate end of the 2021 growing season (November 1, 2021) to the approximate end of the 2023 growing season (October 31, 2023). The experimental design included three nitrogen fertilization treatments (Low: 84 kg N/ha [75 lb. N/acre], Medium: 168–196 kg N/ha [150–175 lb. N/acre], and High: 252–280 kg N/ha [225–250 lb. N/acre]) and three irrigation treatments (No Irrigation [NI]: 0 mm, Limited Irrigation [LI]: 100–200 mm, and Full Irrigation [FI]:> 200 mm) for the two study years. *The Scottsbluff site was used in the 2022 growing season and, in North Platte, the experimental design shown applies only to the 2022 growing season. In 2023, a single field was grown in North Platte with two irrigation treatments (NI = 0 mm and LI = 114 mm) in a randomized incomplete block design with 168 kg N/ha (150 lb. N/acre) nitrogen fertilizer applied. (B) Population‐level yield in each location‐year (*n* = 135–560 plots). The colored points indicate the median yield of the corresponding nitrogen treatment within the location‐year. Box plots indicate the range from the 25th to 75th percentile of values. Black lines within the boxplots indicate the median value. Whiskers indicate the most extreme values within 1.5 times the interquartile range and black points indicate the values of data points outside that range. (C) Estimated proportion of variance explained by environment, genotype, and genotype‐by‐environment interactions for each of the 18 phenotypes measured. Asterisks denote phenotypes measured only in a subset of location‐years. GDD indicates growing degree days.

A set of trait measurements, including combined‐measured grain yield, yield components, and a number of other phenotypes, were collected from a set of 6016 four‐row maize yield plots. These plots were grown as part of multi‐environment field trials of 122 maize hybrids conducted in 2022 and 2023 at field sites in the US Corn Belt near Scottsbluff, Nebraska; North Platte, Nebraska; Lincoln, Nebraska; Missouri Valley, Iowa; Ames, Iowa; and Crawfordsville, Iowa. A number of locations included multiple nitrogen and/or irrigation treatments, resulting in 34 specific environments in which yield and other traits were assayed across the 14 location‐years of the study (Figure 1A). Grain yields (0.91–14.95 MT/ha, Figure 1B) were representative of the range of yields within 95% confidence intervals of county mean yields across Nebraska and Iowa in 2022 and 2023 (3.27–15.96 MT/ha, USDA NASS n.d.). Of the 122 hybrids, 81 were evaluated in all 34 environments. A total of 18 unique phenotypes were scored in these experiments. After the removal of extreme values (0.17%–2.97% per phenotype), the resulting dataset contained 94,053 non‐missing phenotype values.

In a variance paritioning analysis considering environment, genotype, and genotype‐by‐environment as separate factors, differences between environments comprised the largest contributing factor to the variation of 17 of the 18 phenotypes (Figure~\ref{fig:overview}C). The one exception was the phenotype kernel row number were differences between genotypes the most significant contributor to variation (53.2%). Differences between environments explained more than 98% of the variation in growing degree days (GDD) before anthesis and in GDD to silking, and they explained more than 70% of variation in flag leaf and ear heights. Genotype‐by‐environment interactions did not explain the largest proportion of variation in any of the phenotypes tested, but the phenotypes for which this factor played the largest role were ear length (7.7%), test weight (6.9%), and harvest moisture (6.9%). Although numerous components contribute to the environment as a factor, as in the variance partitioning model used here, one well‐known component is nitrogen fertilization, which is expected to have a positive relationship with grain yield.

**FIGURE 2:**
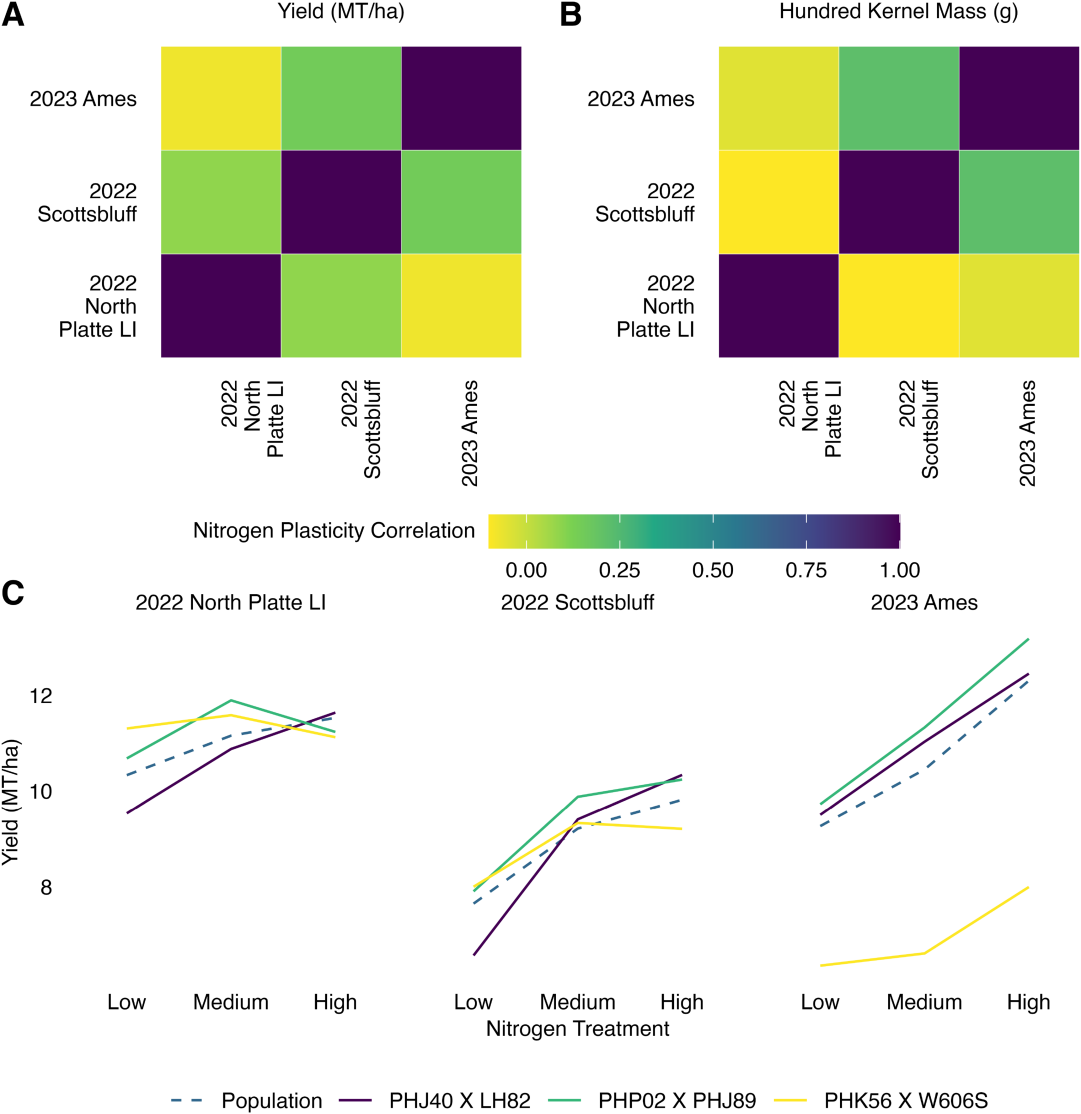
The responses of maize hybrids to nitrogen fertilization differ between location‐years. (A) Spearman rank correlations of yield linear nitrogen plasticity values across all hybrids common to both location‐years for location‐years where a population‐level positive relationship of yield with nitrogen fertilization was observed. (B) Spearman rank correlations of 100 kernel mass linear nitrogen plasticity values across all hybrids common to both location‐years for location‐years where a population‐level positive relationship of yield with nitrogen fertilization was observed. (C) Mean yields of hybrids with the lowest mean nitrogen plasticity (MNP) value (PHK56 X W606S, MNP = 0.89), an intermediate MNP value (PHP02 X PHJ89, MNP = 1.00), and the highest MNP value (PHJ40 X LH82, MNP = 1.11) and population‐level response under different nitrogen fertilization treatments in location‐years where a population‐level positive relationship of yield with nitrogen fertilization was observed.

In 3 of the 10 location‐years with nitrogen treatments (2023 Ames, 2022 Scottsbluff, and 2022 North Platte LI), we observed a positive relationship between nitrogen fertilization rate and yield at the population level (Figure 1B). We fit the Finlay–Wilkinson model for the three environments contained within each of these three location‐years to determine the repeatability of nitrogen responses across location‐years. Linear plasticity for yield across nitrogen application rates did not correlate across the location‐years studied, with Spearman rank correlations for location‐year pairs ranging from −0.07 to 0.15 (Figure 2A). Similar trends were observed for 100 kernel mass (Figure 2B) and other traits (Figure S1).

The nitrogen responses of individual lines to increased nitrogen fertilization differed between location‐years (Figure 2A,B), and sometimes within a location‐year (Figure S2). We selected three lines having the lowest mean nitrogen plasticity (MNP) value (PHK56 X W606S, MNP = 0.89), an intermediate MNP value (PHP02 X PHJ89, MNP = 1.00), and the highest MNP value (PHJ40 X LH82, MNP = 1.11) across the three location‐years (Figure 2C). All three hybrids exhibited multiple patterns of nitrogen response across the three location‐years (Figure 2C), including differential responses to increasing nitrogen fertilization from low to medium levels and from medium to high levels. The repeatability of nitrogen plasticity values within a location‐year for yield was quite low (Spearman *ρ* = 0.0473, Figure S2) when comparing nitrogen plasticities estimated with one block per nitrogen treatment in each of the three location‐years where the population‐level response to nitrogen was positive. For the maize hybrids in our population, both within and across location‐years, the relative nitrogen plasticity for yield showed poor repeatability (−0.07 ≤ *ρ* ≤ 0.17), and it often differed from the population‐level nitrogen response in the given location‐year (Figure 2C).

Given our limited success in studying the response of maize hybrids to a specific environmental factor, we used Finlay–Wilkinson joint regression to investigate the relative response to improved environments of the maize hybrid lines in our population without regard to the specific environmental factor(s) that improved the environment. We excluded the 2022 Lincoln location‐year from Finlay–Wilkinson regression analyses, given its extremely low yield value relative to all other locations (Figure 1B). We found a moderate correspondence (Spearman *ρ* = 0.4483, Figure S3) between the linear plasticity values for each hybrid estimated by two Finlay–Wilkinson models, each using half of the dataset containing one block from each environment. This correspondence is within the range of the Spearman correlations between blocks for the 18 traits directly measured in this study (0.4411–0.9559). This indicates that the linear plasticity values extracted from Finlay–Wilkinson regression models across all 31 environments used are reasonably repeatable and represent a genetically controlled trait.

**FIGURE 3:**
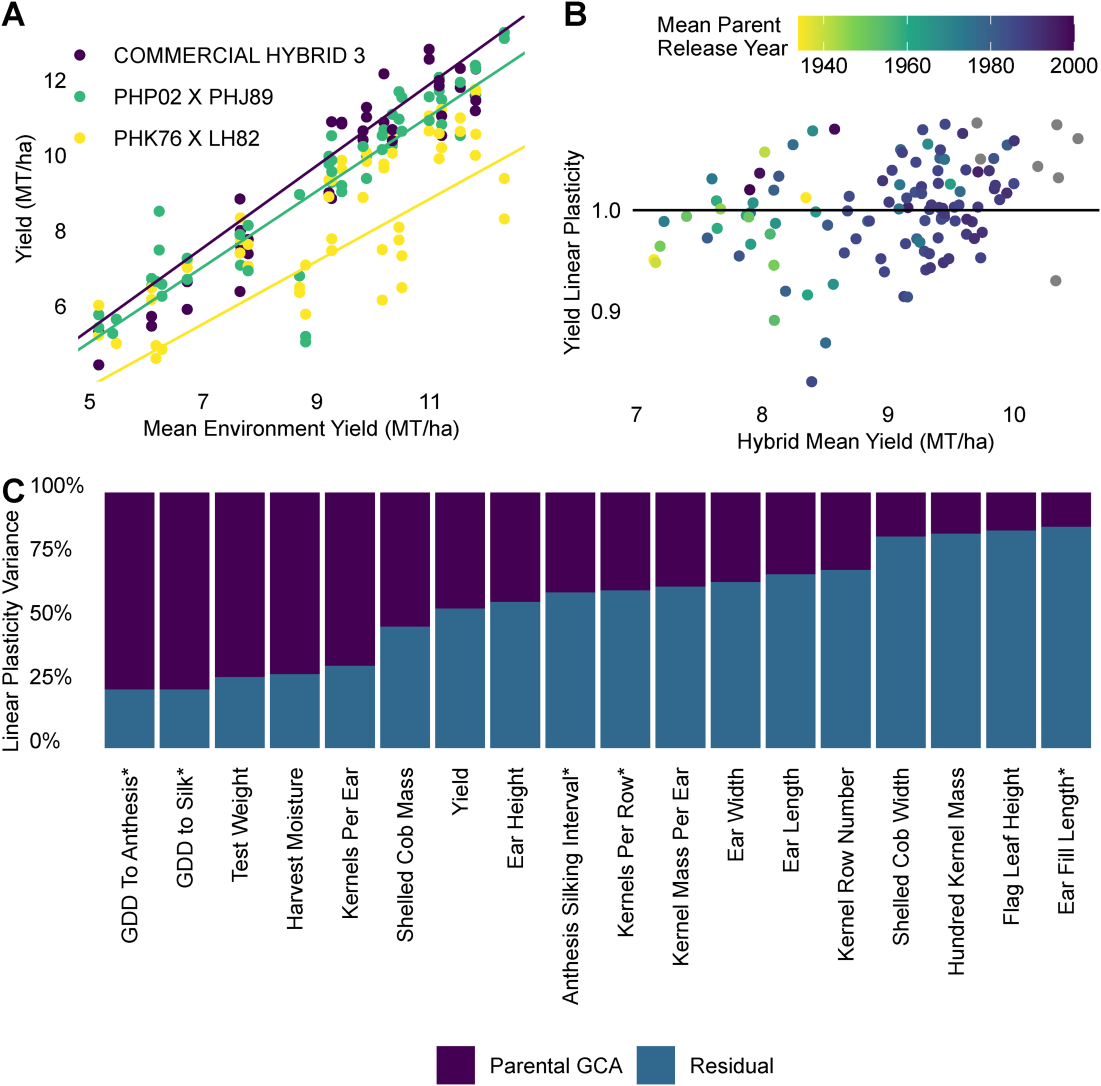
Increased linear plasticity is associated with higher performance across all environments. (A) Performance of lines with the highest (COMMERCIAL HYBRID 3, *b* = 1.09), an intermediate (PHP02 X PHJ89, *b* = 1.00), and the lowest (PHK76 X LH82, *b* = 0.83) Finlay–Wilkinson plasticity values for yield versus mean yield of each environment in this study and their corresponding Finlay–Wilkinson regression lines. (B) Finlay–Wilkinson yield linear plasticity values for each hybrid in this study versus their mean yields across all environments, colored by their mean parent release year. Gray points represent commercial check hybrids for which the inbred parents are unknown. The black horizontal line indicates a linear plasticity value of 1. (C) Proportion of the linear plasticity of yield component traits attributable to the general combining ability (GCA) of the hybrid parents. Parental GCA represents the sum of variances attributed to either the ear parent or the pollen parent GCA. Asterisks denote phenotypes measured in only a subset of location‐years. GDD indicates growing degree days.

Increased phenotypic plasticity is typically assumed to be undesirable because of the concern that lines with high plasticity will perform particularly poorly in poor environments. Figure 3A shows the Finlay–Wilkinson yield regression line for the hybrids with the highest, intermediate, and lowest Finlay–Wilkinson linear plasticity values (slopes) across the tested environments. Of these three lines, the one with the highest plasticity is predicted to have the highest yield in all tested environments, followed by the line with intermediate plasticity, indicating that increased linear plasticity may not be associated with poor performance in poor environments.

In our dataset, yield and linear plasticity were significantly positively correlated (*p* = 1.1 × 10^*−*3^). Hybrids with later mean parent release years tended to have higher yields (*p* < 2.2 × 10^*−*16^), indicating that the association between grain yield and linear plasticity may be due to the effect of artificial selection. The significance of the association between yield and linear plasticity increases marginally when controlling for mean parent release year (*p* < 3.6 × 10^*−*4^, Figure 3B), which ranged from 1934 to 2000 in our dataset. However, the performance of the most plastic or the highest yielding hybrids as a percentage of the environment mean did not drastically increase as the environment mean increased (Figure S4), as would be expected if the increases in linear plasticities were independent of the increases in performance across all environments. The increases in linear plasticity and their positive relationship with overall yield and mean parent release year are at least partially due to increased performance across all environments (Figures S5 and S6), rather than being solely due to an increase in the level of response to favorable environments.

In contrast to the pattern observed for grain yield, the kernel row number, ear length, ear fill length, kernels per row, and kernels per ear did not show significant associations (*p* > 0.05) between mean value and either linear plasticity or mean release year of inbred parents (Figure S7). Mean trait values for ear width (*p* = 0.01), 100 kernel mass (*p* = 0.04), kernel mass per ear (*p* = 3.7 × 10^−5^), and harvest moisture (*p* < 2.0 × 10^−16^) were all significantly positively correlated with linear plasticity, but none of these traits exhibited a significant correlation between the mean value and mean release year of the inbred parents (Figure S7). Flag leaf height, shelled cob width, and shelled cob mass had no significant association with linear plasticity (*p* > 0.05), but they had significant negative associations with mean parent release year (*p* < 1.0 × 10^−3^), indicating that they have declined over time. For test weight, the trait mean has significantly increased over time (*p* = 0.05), but lower linear plasticity was significantly associated with higher overall mean trait values (*p* < 2.0 × 10^−16^), indicating potential indirect selection for high and stable test weights. The overall trait means of ear height (*p* = 4.2 × 10^−15^), GDD to anthesis (4.5 × 10^−6^), GDD to silk (*p* = 1.9 × 10^−7^), and anthesis‐silking interval (*p* = 0.02) have all significantly declined over time. Increased linear plasticity was associated with decreased overall mean anthesis‐silking intervals (*p* = 3.7 × 10^−7^), whereas decreased linear plasticities for ear height (*p* = 3.6 × 10^−3^), GDD to anthesis (*p* = 2.0 × 10^−4^), and GDD to silk (*p* = 3.2 × 10^−3^) were associated with decreased overall mean trait values. The mixed relationships between how the traits have changed over time and linear plasticity with overall trait values indicate that increased or decreased linear plasticities are not universally neutral, advantageous, or disadvantageous for overall performance but, rather, are trait‐dependent (Figure S7).

Having observed the significant relationship between mean parent release year and linear plasticity, we investigated how much variation in linear plasticity could be explained by the particular parental lines used. Parental plasticity GCA explained the most variation in the linear plasticity of GDD to anthesis (77.1%), followed by GDD to silk (77.1%) and test weight (72.3%, Figure 3C), and contributed the least to the variance in the linear plasticity of flag leaf height (14.9%) and ear fill length (13.3%). We found that 45.5% of the variation in the yield linear plasticity could be explained by variation in parental GCA. The relatively large contribution of parental GCA to the linear plasticity of GDD to anthesis, GDD to silk, and test weight indicates the promising potential to modify the level of linear plasticity of hybrids for these traits via parental selection, though it may be more difficult to modify the level of linear plasticity for flag leaf height and ear fill length.

**FIGURE 4:**
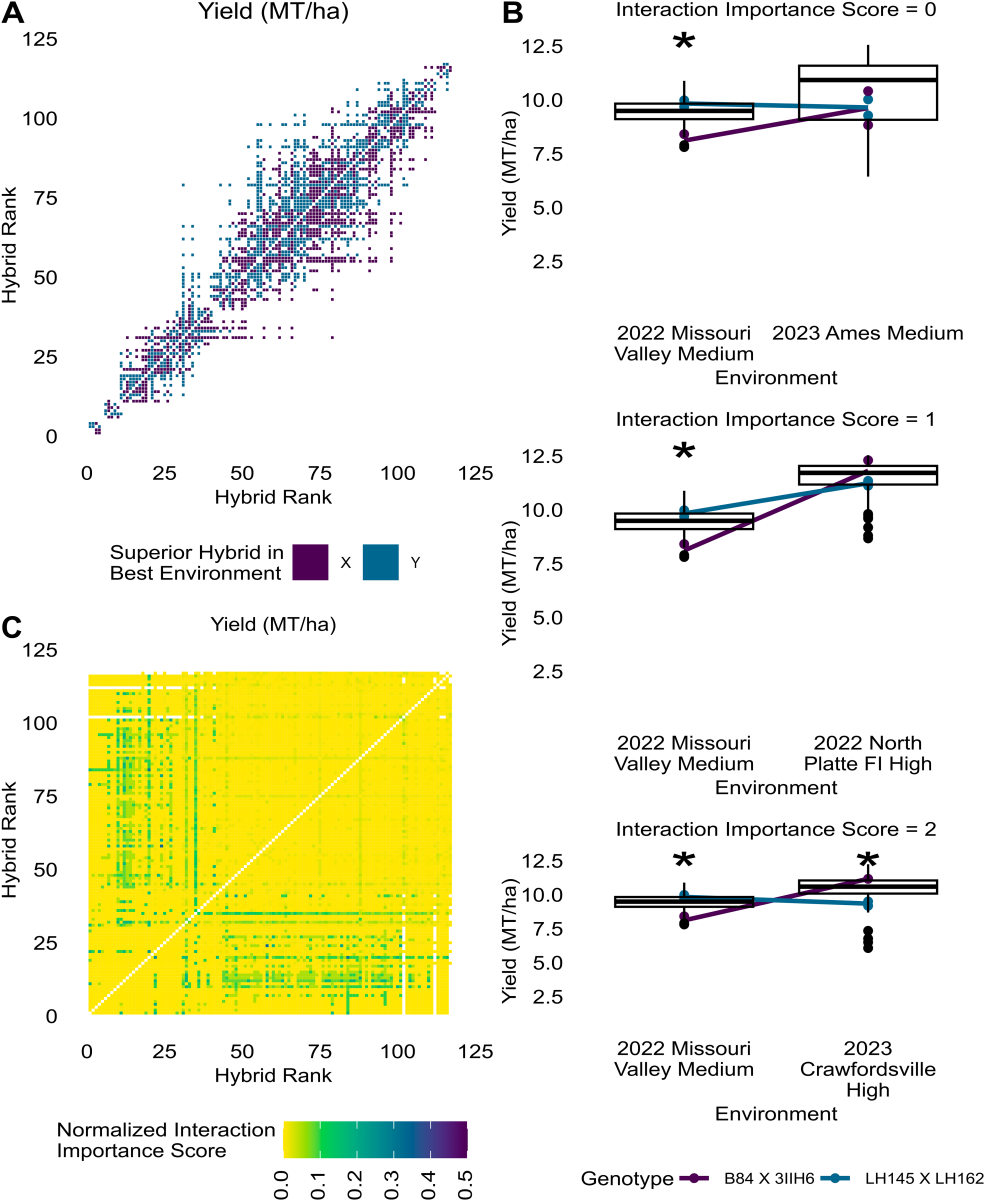
Importance of genotype‐by‐environment interactions to selection decisions regarding a pair of hybrids depends on the environments used for selection. (A) Incidence matrix indicating whether Finlay–Wilkinson regression predicts that the two hybrids will change rank order between the lowest and highest population mean environments used to fit the model. Hybrids are ranked in order of ascending yield BLUP values fitting the environment as a fixed effect. The colored squares indicate a rank order change was predicted for the hybrid pair and the color indicates which hybrid is predicted to have a higher yield in the environment with the highest population mean yield. (B) Performance and genotype‐by‐environment interactions of one hybrid pair across three environment pairs. Box plots show population‐level yield in the given environment (*n* = 163–175 plots). Boxes indicate the range from the 25th to 75th percentile of values. Black lines within the boxplots indicate the median value. Whiskers indicate the most extreme values within 1.5 times the interquartile range and black points indicate the values of data points outside that range. The colored lines connect the mean yields of each hybrid between environments. Asterisks denote a significant difference in mean yields between the two hybrids in the environment at a significance level of *α* = 0.05 according to Tukey's HSD. The top panel shows a pair of environments where the hybrid pair did not change rank order and received an interaction importance score of 0. In the middle panel, the hybrids changed rank order in this pair of environments and had a significant difference in mean yield in one of the two environments, and they received an interaction importance score of 1. The bottom panel shows an environment pair where the hybrids changed rank order and had significant differences in mean yield in both environments, and they received an interaction importance score of 2. (C) Incidence matrix indicating the frequency with which two hybrids exhibited an interaction for yield between two environments that represents a potentially important change in the selection decision between environments. For each hybrid pair, interaction scores were summed across all environment pairs and divided by the total score possible for the hybrid pair based on the number of environments in which both hybrids were present. Hybrids are ranked in order of ascending yield BLUP values fitting the environment as a fixed effect.

Genotype‐by‐environment interactions that result in a change in rank ordering between hybrids within the population of environments have a greater effect on selection decisions within breeding programs than do genotype‐by‐environment interactions that do not result in rank order changes. We found that 14.6% of hybrid pairs were predicted to change rank order between the environments with the lowest and highest population mean yields in the 31 environments used to fit the Finlay–Wilkinson model using their regression‐fitted values (Figure 4A), and that 32.6% of predicted rank order changes were for hybrid pairs with mean yields separated by more than 0.63 MT/ha (10 bushels/acre) in the most favorable environment. The maximum distance between overall ranks for hybrid pairs predicted to have a rank order change was 48 (Figure S8). The hybrids in this pair were predicted to have a mean yield difference of 1.82 MT/ha in the most favorable environment by Finlay–Wilkinson regression. However, their observed mean yields in the most favorable environment differed by 3.57 MT/ha, and their overall mean yields across the 31 environments differed by 1.06 MT/ha. Although this analysis gives useful insight into how often rank order changes may occur because of genotype‐by‐environment interactions within this population of environments, it does not indicate how often they represent meaningful differences between hybrids that may result in different selection decisions depending on the environments used for selection.

The hybrids 2369 X LH123HT (overall rank 58, in order of ascending BLUP) and K201 X OS426 (overall rank 24) exhibited rank order changes in four of the six environment pairs where both hybrids were present, with a significant difference in one of the two environments for pairs of environments, all of which had medium nitrogen application rates (2023 Ames and 2023 Missouri Valley; 2023 Ames and 2023 North Platte NI; 2023 Crawfordsville and 2023 Missouri Valley; 2023 Crawfordsville and 2023 North Platte NI). The change in rank order combined with the significant difference in one environment produced an interaction importance score of 1 for each of these four environment pairs (Figure 4B), and a total interaction importance score equal to 33.3% of the total possible score for this hybrid pair. We estimated the interaction importance scores for all hybrid pairs in all environment pairs where both hybrids were present for all 18 traits, and we provide a detailed description of this method in Methods. Eight other hybrid pairs also had normalized interaction importance scores ≥ 0.20, but these were extremely rare cases, representing only 0.13% of all hybrid pairs. Out of 6786 hybrid pairs, 17.0% exhibited no important interactions, with either no rank order changes across the tested environments or no significant difference in mean yields when a rank order change did occur. Furthermore, 92.5% of hybrid pairs had interaction importance scores less than 5% of the maximum score (Figure 4C), indicating that few of the genotype‐by‐environment interactions would result in a significantly different selection decision had a different environment been used for selection. However, the pattern and level of interaction importance scores varied substantially among traits (Figure S9), with some traits, such as GDD to silk, having interaction importance scores as high as half of the total possible score but being quite rare and concentrated in hybrid pairs where both hybrids were ranked in the upper 50% of hybrids for the trait (Figure S9D). Normalized interaction importance scores ≥ 10% of the total possible score were much more widespread for test weight and harvest moisture than for any other traits, with 9.2% and 7.9% of hybrid pairs in our population, respectively, having normalized interaction importance scores ≥ 0.10 for these traits (Figure S9F,G). Normalized interaction importance scores were lower for kernel mass per ear than for yield (Figure S9Q), indicating both that important genotype‐by‐environment interactions for kernel mass per ear are rarer than for yield, and that plasticity in ear phenotypes may not fully explain plasticity in overall grain yield. It is also notable that few of the hybrid pairs with a relatively high interaction importance score for yield overlapped with the hybrid pairs predicted by Finlay–Wilkinson regression to have a rank order change for yield in the set of environments tested (Figure 4A,C). This may be due to similar performance between many pairs of hybrids that were predicted by Finlay–Wilkinson to have rank order changes (Figures 4A and S8), indicating that few of the Finlay–Wilkinson predicted rank order changes between hybrids in our environments would have resulted in different selection decisions.

## 3 Discussion

Breeders may take several approaches toward genotype‐by‐environment interactions—ignoring them or, most often, minimizing them or exploiting them. Approaches to genotype‐by‐environment interactions in breeding programs that seek to minimize or exploit them assume that they will impact selection decisions. Hybrid pairs in our population rarely had interactions that would significantly change selection decisions due to rank order changes with significant differences in yield in one or more of the environments under comparison (Figure 4C). Though some hybrids in the lower 50% of the population for yield had important genotype‐by‐environment interactions, with a majority of hybrids in the upper 50% of the population for yield, these hybrids represented only 14.5% of our population. The low frequency of genotype‐by‐environment interactions that would change selection decisions revealed an opportunity to reduce the number of environments used for selection. However, it is possible that these results would not translate to an active breeding population. Our population of hybrids was generated using inbred parental lines that were historically patented by either public or private breeding programs over a 68‐year period, with hybrid mean parent release years ranging from 1934 to 2000. Because hybrid mean yield has increased over this period (Figure 3B, Duvick 2005), our population may show larger differences in mean performance between genotypes than a population of elite hybrids originating from the same selection cycle in an active breeding program. This may lead us to observe fewer genotype‐by‐environment interactions that would likely directly affect selection decisions.

The differences between our observation that hybrid mean yield and linear plasticity are positively associated, and observations from previous studies indicating a tradeoff between them may be due to several factors. First, it may be due to differences in the populations sampled, especially for studies, such as ours, that utilized stability measures derived from joint regression models indexed on the population used to fit the model (Figure 3A). Our population of hybrids was generated using inbred parental lines historically patented by either public or private breeding programs; thus, these parental lines represent some of the most elite parental lines existing in breeding programs at the time of patent issuance. Second, our population includes hybrids with mean parent release years ranging from 1934 to 2000, so some effects observed here may be artefacts of different time periods of crop improvement. Third, many studies (Finlay and Wilkinson 1963; Kusmec et al. (2017); Li et al. 2019; Liu et al. 2023), including this study, have indexed the regression based on the range of mean trait values in the range of environments studied, which may also contribute to the low agreement among studies. However, it is also possible that, to some extent, the association we observed between high average yield and high plasticity is an artifact of how plasticity is calculated. Yield as a percentage of the environment mean of either the most plastic or highest overall yielding hybrids does not dramatically increase as the environment mean increases (Figure S4), indicating that the increase in linear plasticity may not be independent of increased performance across all environments.

When we estimated linear plasticity for yield as a percentage of the environment mean, we observed a tradeoff between linear plasticity and both average performance relative to the population and average yield across all environments (Figure S10A,B), as was initially expected. It is typically assumed that a tradeoff between plasticity and overall performance is due to poor performance in poor environments, but if linear plasticity values are negative, this can also indicate reduced performance in improved environments. We observed a tradeoff between linear plasticity for yield as a percentage of the environment mean and average performance relative to the population in the 14 environments with the lowest mean yields out of the 31 environments that were used for plasticity analyses (Figure S10C, Spearman *ρ* = −0.39). In contrast, we observed a positive asymptotic relationship between them in the 14 environments with the highest mean yields (Figure S10D, Spearman *ρ* = 0.42). These relationships also held true when linear plasticity values were compared with mean yields across these environments (Figure S10E,F). Plasticity values estimated in the 14 best environments were far more negative (−5.55 to 1.27; Figure S10D,F) than plasticity values estimated in the 14 poorest environments (0.59–1.56, Figure S10C,E). In all cases, the mean parent release year was significantly positively associated with the measure of overall performance used (*p* < 1.0 × 10^−15^) when controlling for linear plasticity. Relative to the population, newer hybrids are responding less negatively to poor environments and more positively to improved environments than older hybrids (Figure S10).

The measures of linear plasticity we have presented above consider all factors that differ between the environments tested but do not attribute the phenotypic variation to any specific environmental factor. However, in some cases, it may be desirable to modulate the level of plasticity in response to a specific environmental factor, such as the level of nitrogen fertilization. On account of the significant input costs (Plastina 2024), greenhouse gas production (Walling and Vaneeckhaute 2020), and negative environmental (Paudel and Crago 2021) and human health impacts (Y. Wang and Lu 2020) associated with the production and agricultural use of synthetic nitrogen fertilizers, there has recently been increased interest in decreasing their use, but not at the cost of decreasing yield. Developing maize hybrids with yields that remain stable under reduced levels of nitrogen fertilization via traditional selection methods requires accurate measurement of the nitrogen plasticity under environmental conditions relevant to the target environments (Bernardo 2021). Because the true values of phenotypes are often unknown, repeatable measurements are often used instead of accurate measurements. As the genetic repeatability of measurements for a trait increases, the effectiveness of selection also generally increases, given the same selection intensity (Falconer and Mackay 1996). We found the repeatability of nitrogen plasticity for yield to be quite low across location‐years (−0.07 ≤ *ρ* ≤ 0.15, Figure 2A,B) and even within a location‐year (−0.02 ≤ *ρ* ≤ 0.17, Figure S2). The low repeatability of nitrogen plasticity measurements in this study may be due to several causes. First, nitrogen plasticities were calculated using yield estimates from only two replicates of each genotype in each of the three nitrogen levels in a location‐year, so the estimates of the genotype mean yields and resulting nitrogen plasticities may be noisy. However, two replicates in each environment per genotype were sufficient to repeatably measure complex traits such as yield and ear height in high nitrogen‐fertilization environments with Spearman rank correlations within an environment of 0.30 ≤ *ρ* ≤ 0.77 and 0.20 ≤ *ρ* ≤ 0.70, respectively. In 33% of cases, nitrogen plasticities correlated better between location‐years using two replicates per genotype in each environment than within a location‐year using one replicate per genotype in each environment (Figure 2A, Figure S2). This indicates that increasing the number of replicates can improve the repeatability of yield nitrogen plasticity by increasing the repeatability of the grain yield measurements. It is unclear how many replications of a genotype per environment would be required to measure nitrogen plasticity sufficiently repeatably to effectively select for nitrogen stability.

This study showed that hybrid maize grain yield and linear plasticity increased together during US hybrid maize breeding between 1934 and 2000 (Figure 3B) and that performance increased in all environments (Figure S6). Improved performance in all environments has contributed to increased linear plasticity with overall yield (Figure S4). There are two divergent paths toward increasing the environmental sustainability of maize production, specifically and crop production generally. First, breeders can seek to reduce the required inputs given the same land area. This approach would require breeding lines with stable yields under reduced fertilizer and irrigation inputs. We found nitrogen plasticity to be poorly repeatable within and across location‐years (Figures 2 and S2), making selection for nitrogen‐stable lines a key component of this strategy difficult. Though adding replicates or additional nitrogen fertilization rates may increase the repeatability of this trait, it would also increase the cost of field trials used for selection. However, this may not be necessary because the performance in poor environments has not decreased despite increases in plasticity (Figures 3A,B and S6) and performance has improved across all environments (Figures S5 and S4). The second possible approach is to focus on breeding for increased production in areas of high potential yield. The observed improvements in performance across all environments suggest that selection for performance in high‐yield‐potential environments may also improve performance in marginal environments. The environmental impact per bushel of corn produced tends to be lower in high‐productivity systems than in low‐yield systems (Grassini and Cassman 2012). Ideally, this would also allow for better containment and mitigation of environmental impacts, such as nitrate leaching via concentrated efforts. This approach would create incentives to breed for hybrids that not only can respond strongly to highly favorable environments but also can tolerate high‐intensity production practices such as high‐density planting.

## 4 Methods

### 4.1 Maize Field Experiments and Data Processing

Forty‐eight were drawn from a set of hybrids that have been repeatedly used in the multi‐year, multi‐site Genomes to Fields Initiative (AlKhalifah et al. 2018; McFarland et al. 2020; Lima et al. 2023a, 2023b); 36 are “Early Release” hybrids created using Corn Belt inbreds released before the 1950s, and the remainder are hybrids that have been previously utilized in other studies by our research group. The 115 non‐commercial maize hybrids used in the Genomes to Fields Initiative trials, nitrogen yield trials, and “Early Release” hybrids were created by crossing 52 inbred maize lines released under a now expired Plant Variety Protection certificate, which we refer to as ex‐PVP inbred lines, or released without intellectual property protection, typically by a public sector breeding program. Each inbred served as a parent for 1 to 13 hybrids. Individual hybrids are described in Table S1. The mean parent release year for each hybrid was calculated as the mean of the release years of the parental inbred lines. The parental line release year was determined using the information available for the parental lines on the US National Plant Germplasm System (NPGS) website (https://npgsweb.ars‐grin.gov/gringlobal/search). When no release date or intellectual property right certificate was available, the earliest date of the receipt of the line or donation to NPGS was used. For detailed information on the parental inbred lines used to generate the hybrids in this study, please see Table S2. Resequencing data for 47 of the 52 parental lines is available in Grzybowski et al. (2023), and the parental inbred lines used in this study can be matched to the inbred lines in the resequencing SNP set using Table S3.

We defined a location‐year as a unique combination of location, year, and irrigation level, as irrigation was mostly confounded with location and year in our study (Figure 1A). Table 1 describes the irrigation provided and the nitrogen fertilization rate(s) in each location‐year. We defined an environment as a unique combination of location‐year and nitrogen fertilization level. Within each environment, a randomized complete block design with two blocks was utilized. All field plots were four‐row plots with 76.2 cm (30 in.) row spacing, with a targeted planting density of 91,063–98,125 plants/ha. In Scottsbluff during the 2022 growing season, all field plots were 6.86 m (22.5 ft.) long (planted length). In all other location‐years, all field plots were 5.33 m (17.5 ft.) long. Figure 1A shows a geographic overview of the experimental design employed in this study. The map of precipitation in Nebraska and Iowa in Figure 1A was produced using data from gridMET (Abatzoglou 2013) and the packages AOI (Johnson 2024a), climateR (Johnson 2024b), zonal (Johnson 2024c), and sf (Pebesma 2018). We integrated county mean daily precipitation over the period of November 1, 2021–October 31, 2023, which coincides with the approximate end of the growing season prior to this two‐year study and the approximate end of the last growing season in this study. All other data visualizations were produced using the packages patchwork (Pedersen 2024), viridis (Garnier et al. 2023), scales (Wickham et al. 2023), and cowplot (Wilke 2020). Code for all data visualizations is available in Information S1.

**TABLE 1:** Summary of irrigation provided and nitrogen fertilization rate(s) in each location‐year.

Location‐year	Irrigation provided (mm)	Nitrogen fertilization rates (kg N/ha)	Planting date	Harvest date
2022 Scottsbluff	429	Low: 84 (75 lb. N/acre)	May 19, 2022	November 9–10, 2022
		Medium: 168 (150 lb. N/acre)		
		High: 252 (225 lb. N/acre)		
2022 North Platte No Irrigation (NI)	0	Low: 84 (75 lb. N/acre)	May 18, 2022	October 19–24, 2022
		Medium: 84 (75 lb. N/acre)		
		High: 252 (225 lb. N/acre)		
2022 North Platte Limited Irrigation (LI)	109	Low: 84 (75 lb. N/acre) Medium: 84 (75 lb. N/acre)	May 17, 2022	October 26–28, 2022
		High: 252 (225 lb. N/acre)		
2022 North Platte Full	218	Low: 84 (75 lb. N/acre)	May 17, 2022	October 31–November 1, 2022
Irrigation (FI)		Medium: 84 (75 lb. N/acre)		
		High: 252 (225 lb. N/acre)		
2022 Lincoln	0	Low: 84 (75 lb. N/acre)	May 22, 2022	October 10, 2022
		Medium: 84 (75 lb. N/acre)		
		High: 252 (225 lb. N/acre)		
2022 Missouri Valley	0	Medium: 196 (175 lb. N/acre)	April 29, 2022	October 11, 2022
2022 Ames	0	Low: 84 (75 lb. N/acre)	May 22–23, 2022	October 12–16, 2022
		Medium: 84 (75 lb. N/acre)		
		High: 280 (250 lb. N/acre)		
2022 Crawfordsville	0	Low: 84 (75 lb. N/acre)	May 11, 2022	October 7, 2022
		Medium: 84 (75 lb. N/acre)		
		High: 252 (225 lb. N/acre)		
2023 North Platte No Irrigation (NI)	0	Medium: 168 (150 lb. N/acre)	May 10, 2023	October 19–20, 2023
2023 North Platte Limited Irrigation (LI)	114	Medium: 168 (150 lb. N/acre)	May 10, 2023	October 19–20, 2023
2023 Lincoln	0	Low: 84 (75 lb. N/acre)	May 16, 2023	October 23, 2023
		Medium: 84 (75 lb. N/acre)		
		High: 252 (225 lb. N/acre)		
2023 Missouri Valley	0	Medium: 179 (160 lb. N/acre)	May 10, 2023	September 25, 2023
2023 Ames	0	Low: 84 (75 lb. N/acre)	May 19, 2023	October 19, 2023
		Medium: 84 (75 lb. N/acre)		
		High: 252 (225 lb. N/acre)		
2023 Crawfordsville	0	Low: 84 (75 lb. N/acre)	May 4, 2023	October 2, 2023
		Medium: 84 (75 lb. N/acre)		
		High: 252 (225 lb. N/acre)		

In 2022, 84 maize hybrids were grown in 6 locations (Scottsbluff, Nebraska; North Platte, Nebraska; Lincoln, Nebraska; Missouri Valley, Iowa; Ames, Iowa; and Crawfordsville, Iowa). In 2023, 81 of these same hybrids and an additional 38 hybrids were grown in five locations (North Platte, Nebraska; Lincoln, Nebraska; Missouri Valley, Iowa; Ames, Iowa; and Crawfordsville, Iowa). Within a block, each genotype present during that growing season was represented at least once, except for the environments in North Platte during the 2023 growing season because of field space constraints. Specifically, two genotypes were absent from the 2023 North Platte LI location‐year, and 36 hybrids were absent from the 2023 North Platte NI location‐year. In North Platte, during the 2022 growing season, the non‐irrigated trial was spatially separated from the irrigated trials. In Ames during the 2022 growing season, the 84 kg N/ha (75 lb. N/acre) trial was spatially separated from the 168 and 280 kg N/ha (150 and 250 lb. N/acre) trials.

For detailed information regarding agronomic practices, trait data collection, and thresholds for removing extreme values, please see Information S2. Briefly, measurements included the number of standing plants in the center two rows of the plot, the height of attachment of the primary ear, and the height of the flag leaf of representative plants in each plot during the growing season. Additionally, the date that 50% of the plants in a plot reached anthesis and silking was recorded in the Scottsbluff, North Platte, and Lincoln locations. After the majority of plants in the field had senesced, four representative ears were harvested by hand from the center of the outer two rows of each plot, artificially dried, and phenotyped. Following hand harvest of the representative ears, the center two rows of the plot were mechanically harvested by a plot combine. The plot‐level grain yield and flowering time data from the 2022 growing season were previously described in Shrestha et al. (2024). Data from all environments were combined using R version 4.3.2 (R Core Team 2023) using the packages tidyverse (Wickham et al. 2019), readxl (Wickham and Bryan 2023), lubridate (Grolemund and Wickham 2011), and weathermetrics (Anderson et al. 2013). Information S1 contains all code for combining the data and analyses described below. Ear phenotype values were averaged within each plot to produce a single value per plot. GDDs were estimated from temperature data collected by in‐field weather stations (Watchdog 2700) and imputed with data from NASA POWER (Sparks 2018) with *T*_base_ = 50*°*F (10°C) and *T*_opt_ = 86*°*F (30°C). In the 2022 Crawfordsville location‐year, the weather station malfunctioned, so the weather data from a Genomes to Fields weather station of the same brand and model located approximately 0.5 km away were used (Genomes to Fields 2022 dataset 2023). The yield per acre (15.5% moisture on a wet basis and 56‐lb bushels) was calculated from the grain weight and grain moisture content estimated by the combine during the mechanical harvest of the center two rows of the plot and converted to MT/ha by multiplying by 0.0628. The distribution of values for each trait within a location‐year was visually examined, and extreme values were removed as described in Information S2. In cases where a single extreme value for an ear phenotype caused an extreme mean value, the mean of the remaining values for the ear phenotype was used. Within each environment, all phenotype values were spatially corrected using SpATS (Rodríguez‐Álvarez et al. 2018), fitting the plot identifier as the genotype random effect to obtain plot‐level coefficients plus the model intercept used for all downstream analyses. Raw and spatially corrected values for all traits are available in Table S4. Five hybrids were present in fewer than three environments and dropped for all downstream analyses, leaving 117 hybrids that were each observed in a minimum of four environments.

### 4.2 Variance Partitioning

The proportions of variance of each phenotype that could be attributed to environment, genotype, and the interaction between genotype and environment were estimated by fitting the mixed linear model described by Equation (1), where *Y*_*ijk*_ is the performance of the *i*th genotype in the *j*th environment, *g*_*i*_ is the random effect of the *i*th genotype, *e*_*j*_ is the random effect of the *j*th environment, *η*_*ij*_ is the random effect of the interaction between the *i*th genotype and the *j*th environment, and *ε*_*ijk*_ is the deviation of the *k*th individual of the *i*th genotype in the *j*th environment from the regression‐fitted value. Model fitting was done using the lme4 R package (Bates et al. 2015). The proportion of variance attributed to each effect was estimated by dividing the variance of the effect by the total phenotypic variance.

(1) $$Y_{\mathrm{ijk}}=g_{i}+e_{j}+\eta_{\mathrm{ij}}+\varepsilon_{\mathrm{ijk}}$$

The proportion of variance in linear plasticity attributable to the GCA of the parents was estimated as described above, substituting the model described by Equation (1) with Equation (2), where *B*_*ij*_ is the linear plasticity value for the hybrid with the *i*th ear parent and the *j*th pollen parent, *e*_*i*_ is the random effect of the *i*th ear parent, *p*_*j*_ is the random effect of the *j*th pollen parent, and *ε*_*ij*_ is the deviation of the linear plasticity value of the hybrid with the *i*th ear parent and *j*th pollen parent from the regression‐fitted value.

(2) $$B_{\mathrm{ij}}=e_{i}+p_{j}+\varepsilon_{\mathrm{ij}}$$

Because some parental lines were used as both ear parents and pollen parents in the creation of this hybrid population, we summed the proportions of variance attributed to *e*_*i*_ and *p*_*j*_ for a single estimate of the variance in linear plasticity attributable to the GCA of the parental inbred lines.

### 4.3 Estimation of Linear Plasticity

The linear plasticity of each hybrid was estimated using the R package spFW (Guo 2022), ignoring spatial effects to fit the Finlay–Wilkinson joint regression model with the phenotype of interest as the response. Because the 2022 Lincoln location‐year environments had significantly lower yield on a population level than all other location‐years (Figure 1B), we did not include observations from the 2022 Lincoln environments in the data used to fit the model. The *b* value for each hybrid was extracted and regarded as the linear plasticity value. To make the data easier to interpret, 1 was added to all linear plasticity values.

The repeatability of linear plasticity values was estimated as the Spearman *ρ* between linear plasticity values for the hybrids obtained by fitting two Finlay–Wilkinson models, each using data from one block from each environment. The estimated Finlay–Wilkinson regression‐fitted values for each hybrid were used to estimate whether there was a predicted rank order change between the hybrids when comparing the environment with the lowest population mean performance and the environment with the highest population mean performance. To estimate the relationship between overall mean trait value, linear plasticity, and mean parent release year, we performed an analysis of variance using lm() and anova() functions in R, using linear plasticity and mean parent release year as predictors and the overall mean trait value for the hybrid as the response. The overall rank of each hybrid across all studied environments was determined using a best linear unbiased prediction (BLUP) model with environment as a fixed effect. Overall rank was assigned in order of ascending BLUP values, with the hybrid having the minimum BLUP assigned a rank of 1.

For location‐years where a positive relationship between grain yield and nitrogen fertilization rate was observed on a population level (2022 Scottsbluff, 2022 North Platte LI, and 2023 Ames), nitrogen linear plasticity was determined as described above using only data from the given location‐year. A total of 31 hybrids were present only under medium nitrogen in the 2023 location‐years and were dropped from the analysis prior to estimating nitrogen plasticity. Correlation of nitrogen linear plasticity values between location‐years was estimated using Spearman's *ρ* with complete observations. The repeatability of nitrogen plasticity values was estimated as described above. The Spearman *ρ* between the linear plasticities estimated using each half of the dataset at a given location‐year was estimated using all three location‐years where a positive relationship between grain yield and nitrogen fertilization rate was observed on a population level.

For each hybrid, the hybrid mean yield within each environment as a percentage of the mean of all hybrids present in the environment was estimated. The line of best fit and associated 95% confidence intervals for this performance measure (as a function of environment) were estimated using the lm() function in R for each hybrid in the upper and lower 10% of all hybrids for either linear plasticity or yield across all environments. Linear plasticity for yield as a percentage of the environment mean was estimated using the lm() function in R to fit a model with the grain yield as a percentage of the environment mean as the response and the hybrid, the environment mean, and the interaction between the hybrid and the environment mean as predictors. The linear plasticity on a percent mean basis was taken as the environment mean interaction coefficient for the respective hybrid plus the population‐level environment mean coefficient plus 1 to center the linear plasticity values near 1 for interpretability.

### 4.4 Interaction Importance Scoring

The importance of the genotype‐by‐environment interaction between two hybrids in a pair of environments for informing selection decisions for a phenotype was scored using the following procedure. For each environment, an ANOVA was performed using the aov() function in R with genotype as the sole predictor. Tukey's Honest Significance Difference (HSD) test was used to test all pairwise comparisons between hybrids. A pair of hybrids was considered to significantly differ in this environment if the adjusted *p* value was less than 0.05. For each pair of environments in which a hybrid pair was present, the pair of hybrids received a score of 0 if there was no rank order change between the two environments. If the rank order of the two hybrids changed between the two environments, the pair of hybrids received a score equal to the number of environments where there was a significant difference between the hybrids (0, 1, or 2). Figure 4B shows the performance and corresponding interaction importance scores for one hybrid pair in three pairs of environments. The overall score for the interaction between a pair of hybrids was calculated as the sum of the interaction importance scores across all environment pairs in which the pair of hybrids was present, and this score was normalized by dividing by the total possible score for the hybrid pair ([*n*_*E*_ *×* (*n*_*E*_ *−* 1)] where *n*_*E*_ is the number of environments in which a hybrid pair was present). Interaction scores for all hybrid pairs in all pairs of environments are available in Information S3.

## Author Contributions

P.S.S. and J.C.S. conceived of the project. J.M.D., L.M.C., J.T., L.L.C., K.L., C.U., and D.K.S. conducted experiments and generated data. J.M.D., L.M.C., J.T., K.L., C.U., and J.C.S. processed data. J.C.S. and P.S.S. provided supervision, guidance, and feedback on the design of analyses and approaches. J.M.D. conducted statistical analyses and visualized results. J.M.D. drafted the paper with input from J.C.S. All authors contributed to the revision of the final manuscript.

## Conflicts of Interest

James C. Schnable has equity interests in Data2Bio LLC and Dryland Genetics LLC and has performed paid work for Alphabet. Patrick S. Schnable is a co‐founder and CEO of Dryland Genetics Inc. and a co‐founder and managing partner of Data2Bio LLC. He serves as a member of the scientific advisory boards of Kemin Industries and Centro de Tecnologia Canavieira and is a recipient of research funding from Iowa Corn and Bayer Crop Science. He also serves as a consultant and/or expert witness in intellectual property disputes between seed companies. The authors declare no other conflicts of interest.

## Supporting information


Data S1.

Table S2.

Table S3.

Table S4.

Data S2.

## Data Availability

All data and code are publicly available from https://github.com/jdavis‐132/hips. Supporting Information contains Tables S1–S4, Information S1–S3, and Figures S1–S10.
