# Supplementary material for: Assessing the Impact of Yield Plasticity on Hybrid Performance in Maize
Source: Physiol Plant. 2025 May 27;177(3):e70278. doi: 10.1111/ppl.70278 (PMC12117174; doi:10.1111/ppl.70278)
Supplement: Supplementary file 1 — Data S1. [file PPL-177-e70278-s001.pdf]

Assessing the impact of yield plasticity on hybrid performance in maize

Jensina M. Davis[1,2,3,4], **Lisa M. Coffey**[5], **Jonathan Turkus**[1,2,3], **Lina López-Corona**[1,2,3,6], **Kyle Linders**[1,2,3], **Chidanand Ullagaddi**[1,2,3], **Dipak K. Santra**[3,7], **Patrick S. Schnable**[5], **and James C. Schnable**[1,2,3,4,*]

[1]Quantitative Life Sciences Initiative, University of Nebraska-Lincoln, Lincoln, NE, 68588 USA
[2]Center for Plant Science Innovation, University of Nebraska-Lincoln, Lincoln, NE, 68588 USA
[3]Department of Agronomy and Horticulture, University of Nebraska-Lincoln, Lincoln, NE, 68588 USA
[4]Complex Biosystems Graduate Program, University of Nebraska-Lincoln, Lincoln, NE, 68588 USA
[5]Department of Agronomy, Iowa State University, Ames, IA, 50011 USA
[6]US Department of Agriculture Agricultural Research Service, Plant Science Research Unit, Raleigh, NC, 27607 USA
[7]Panhandle Research and Extension Center, University of Nebraska-Lincoln, Scottsbluff, NE, 69361 USA
[*]Corresponding author: schnable@unl.edu

Table S1. **122 maize hybrids used in this study.**

Hybrid	Ear Parent Age	Pollen Parent Age	Environments Studied	Hybrid Group	Ever in Genomes to Fields Experiments
'IOWA I 205' X I159	1934	1998	5	Early Release	
2369 X 3IIH6	1988	1993	34	Has been used in G2F 'Yellow Stripe'	Yes
2369 X LH123HT	1988	1983	34	Has been used in G2F 'Yellow Stripe'	Yes
2369 X PHN82	1988	1989	34	Has been used in G2F 'Yellow Stripe'	Yes
2369 X PHP02	1988	1988	34	Used in past nitrogen study yield trials	
2369 X PHZ51	1988	1986	34	Has been used in G2F 'Yellow Stripe'	Yes
2FACC X 3IIH6	1989	1993	34	Has been used in G2F 'Yellow Stripe'	Yes
4N506 X 3IIH6	1989	1993	34	Has been used in G2F 'Yellow Stripe'	Yes
66 X A344	2002	1996	5	Early Release	
66 X B7	2002	1968	5	Early Release	
66 X CI 3A	2002	1945	5	Early Release	
66 X I159	2002	1998	5	Early Release	
66 X K201	2002	1963	5	Early Release	
66 X OS426	2002	1934	5	Early Release	
A344 X KYS	1996	1963	2	Early Release	
A344 X OS426	1996	1934	4	Early Release	
B105 X 3IIH6	1996	1993	34	Has been used in G2F 'Yellow Stripe'	Yes
B14A X MO17	1962	1964	34	Has been used in G2F 'Yellow Stripe'	Yes
B37 X H95	1958	1968	34	Has been used in G2F 'Yellow Stripe'	Yes
B37 X MO17	1958	1964	34	Has been used in G2F 'Yellow Stripe'	Yes
B37 X OH43	1958	1949	34	Has been used in G2F 'Yellow Stripe'	Yes
B7 X A344	1968	1996	5	Early Release	
B7 X I159	1968	1998	2	Early Release	

Continuation of Supplemental Table S1					
**Hybrid**	**Ear Parent Age**	**Pollen Parent Age**	**Environments Studied**	**Hybrid Group**	**Ever in Genomes to Fields Experiments**
B7 X KYS	1968	1963	1	Early Release	
B7 X OS426	1968	1934	5	Early Release	
B73 X 3IIH6	1972	1993	34	Has been used in G2F 'Yellow Stripe'	Yes
B73 X MO17	1972	1964	34	Has been used in G2F 'Yellow Stripe'	Yes
B73 X PHM49	1972	1988	34	Has been used in G2F 'Yellow Stripe'	Yes
B73 X PHN82	1972	1989	34	Has been used in G2F 'Yellow Stripe'	Yes
B73 X PHZ51	1972	1986	34	Has been used in G2F 'Yellow Stripe'	Yes
B84 X 3IIH6	1978	1993	34	Has been used in G2F 'Yellow Stripe'	Yes
C.I. 540 X I159	1948	1998	5	Early Release	
C.I. 540 X OS426	1948	1934	5	Early Release	
CI 3A X I159	1945	1998	5	Early Release	
COMMERCIAL HYBRID 1			34	Commercial Hybrid	
COMMERCIAL HYBRID 2			22	Commercial Hybrid	
COMMERCIAL HYBRID 3			22	Commercial Hybrid	
COMMERCIAL HYBRID 4			34	Commercial Hybrid	
COMMERCIAL HYBRID 5			12	Commercial Hybrid	
COMMERCIAL HYBRID 6			34	Commercial Hybrid	
COMMERCIAL HYBRID 7			12	Commercial Hybrid	
F42 X MO17	1983	1964	34	Has been used in G2F 'Yellow Stripe'	Yes
F42 X OH43	1983	1949	34	Has been used in G2F 'Yellow Stripe'	Yes
K201 X A344	1963	1996	5	Early Release	
K201 X I159	1963	1998	5	Early Release	
K201 X OS426	1963	1934	5	Early Release	
K64 X 66	1956	2002	5	Early Release	
K64 X A344	1956	1996	5	Early Release	
K64 X B7	1956	1968	5	Early Release	
K64 X CI 3A	1956	1945	5	Early Release	
K64 X I159	1956	1998	5	Early Release	
K64 X K201	1956	1963	4	Early Release	
K64 X KYS	1956	1963	5	Early Release	
K64 X OS426	1956	1934	5	Early Release	
KYS X I159	1963	1998	5	Early Release	
KYS X OS426	1963	1934	5	Early Release	
L 289 X I159	1934	1998	4	Early Release	
L 289 X OS426	1934	1934	5	Early Release	
LH123HT X 3IIH6	1983	1993	34	Has been used in G2F 'Yellow Stripe'	Yes
LH123HT X PHB47	1983	1983	34	Used in past nitrogen study yield trials	Reciprocal
LH145 X LH162	1983	1990	34	Has been used in G2F 'Yellow Stripe'	Yes
LH185 X LH145	1993	1983	32	Has been used in G2F 'Yellow Stripe'	Yes
LH185 X LH82	1993	1984	34	Used in past nitrogen study yield trials	
LH185 X W606S	1993	1963	31	Used in past nitrogen study yield trials	
LH195 X 3IIH6	1989	1993	34	Has been used in G2F 'Yellow Stripe'	Yes
LH195 X LH123HT	1989	1983	34	Has been used in G2F 'Yellow Stripe'	Yes
LH195 X LH185	1989	1993	34	Has been used in G2F 'Yellow Stripe'	Yes
LH195 X PHM49	1989	1988	34	Has been used in G2F 'Yellow Stripe'	Yes
LH195 X PHZ51	1989	1986	34	Has been used in G2F 'Yellow Stripe'	Yes
LH198 X PHB47	1991	1983	34	Used in past nitrogen study yield trials	

Continuation of Supplemental Table S1					
**Hybrid**	**Ear Parent Age**	**Pollen Parent Age**	**Environments Studied**	**Hybrid Group**	**Ever in Genomes to Fields Experiments**
LH198 X PHZ51	1991	1986	34	Has been used in G2F 'Yellow Stripe'	Yes
LH74 X PHM49	1981	1988	34	Has been used in G2F 'Yellow Stripe'	Yes
LH74 X PHN82	1981	1989	34	Has been used in G2F 'Yellow Stripe'	Yes
LH82 X PHB47	1984	1983	34	Has been used in G2F 'Yellow Stripe'	Yes
LH82 X PHJ89	1984	1991	34	Used in past nitrogen study yield trials	
LH82 X W606S	1984	1963	34	Used in past nitrogen study yield trials	
N209 X 3IIH6	1997	1993	34	Has been used in G2F 'Yellow Stripe'	Yes
ND203 X I159	1950	1998	1	Early Release	
OS426 X 'IOWA I 205'	1934	1934	5	Early Release	
OS426 X A12	1934	1965	2	Early Release	
OS426 X CI 3A	1934	1945	5	Early Release	
OS426 X I159	1934	1998	5	Early Release	
PHB47 X 3IIH6	1983	1993	34	Has been used in G2F 'Yellow Stripe'	Yes
PHB47 X LH185	1983	1993	34	Has been used in G2F 'Yellow Stripe'	Yes
PHB47 X PHJ89	1983	1991	34	Used in past nitrogen study yield trials	Reciprocal
PHB47 X PHK56	1983	1990	34	Has been used in G2F 'Yellow Stripe'	Yes
PHG39 X PHM49	1983	1988	34	Has been used in G2F 'Yellow Stripe'	Yes
PHG39 X PHN82	1983	1989	22	Has been used in G2F 'Yellow Stripe'	Yes
PHJ40 X LH82	1986	1984	34	Has been used in G2F 'Yellow Stripe'	Yes
PHK56 X 3IIH6	1990	1993	34	Used in past nitrogen study yield trials	
PHK56 X LH145	1990	1983	33	Has been used in G2F 'Yellow Stripe'	Yes
PHK56 X LH185	1990	1993	34	Used in past nitrogen study yield trials	
PHK56 X LH198	1990	1991	34	Used in past nitrogen study yield trials	Reciprocal
PHK56 X LH82	1990	1984	34	Used in past nitrogen study yield trials	
PHK56 X PHJ89	1990	1991	34	Used in past nitrogen study yield trials	
PHK56 X W606S	1990	1963	34	Used in past nitrogen study yield trials	
PHK76 X 3IIH6	1987	1993	34	Used in past nitrogen study yield trials	
PHK76 X LH145	1987	1983	34	Used in past nitrogen study yield trials	
PHK76 X LH198	1987	1991	34	Used in past nitrogen study yield trials	Reciprocal
PHK76 X LH82	1987	1984	34	Used in past nitrogen study yield trials	
PHK76 X W606S	1987	1963	34	Used in past nitrogen study yield trials	
PHN46 X PHB47	1990	1983	34	Used in past nitrogen study yield trials	
PHN46 X PHK56	1990	1990	34	Used in past nitrogen study yield trials	
PHN46 X W606S	1990	1963	34	Used in past nitrogen study yield trials	

Continuation of Supplemental Table S1					
**Hybrid**	**Ear Parent Age**	**Pollen Parent Age**	**Environments Studied**	**Hybrid Group**	**Ever in Genomes to Fields Experiments**
PHP02 X LH145	1988	1983	34	Used in past nitrogen study yield trials	
PHP02 X LH185	1988	1993	34	Used in past nitrogen study yield trials	
PHP02 X LH198	1988	1991	34	Used in past nitrogen study yield trials	
PHP02 X LH82	1988	1984	34	Used in past nitrogen study yield trials	
PHP02 X PHB47	1988	1983	34	Has been used in G2F 'Yellow Stripe'	Yes
PHP02 X PHG47	1988	1986	34	Has been used in G2F 'Yellow Stripe'	Yes
PHP02 X PHJ89	1988	1991	34	Used in past nitrogen study yield trials	
PHP02 X PHK56	1988	1990	34	Used in past nitrogen study yield trials	
PHP02 X PHK76	1988	1987	34	Used in past nitrogen study yield trials	
PHP02 X PHN46	1988	1990	34	Used in past nitrogen study yield trials	
PHP02 X W606S	1988	1963	34	Used in past nitrogen study yield trials	
PHT69 X 3IIH6	1992	1993	34	Has been used in G2F 'Yellow Stripe'	Yes
PHW52 X LH185	1988	1993	34	Has been used in G2F 'Yellow Stripe'	Yes
PHW52 X PHM49	1988	1988	34	Has been used in G2F 'Yellow Stripe'	Yes
PHW52 X PHN82	1988	1989	34	Has been used in G2F 'Yellow Stripe'	Yes
PHW52 X PHZ51	1988	1986	34	Has been used in G2F 'Yellow Stripe'	Yes
PHZ51 X LH145	1986	1983	34	Has been used in G2F 'Yellow Stripe'	Yes
WF9 X H95	1943	1968	34	Has been used in G2F 'Yellow Stripe'	Yes

Table S2. Detailed information on parental lines used to create hybrid population used in this study. (Provided as a supplemental Excel file.)

Table S3. Conversion table linking names used for parental inbred lines in Supplemental Table S4 and the resequencing data available from Grzybowski et al. 2023. (Provided as a supplemental Excel file.)

Table S4. Raw and spatially corrected phenotype values. (Provided as a supplemental Excel file.)

Information S1. Code used for data processing and analysis.

Information S2. Detailed information on data collection and processing. (Provided as supplemental pdf file.)

Information S3. Interaction importance scores for all hybrid pairs in all pairs of environments for all phenotypes.

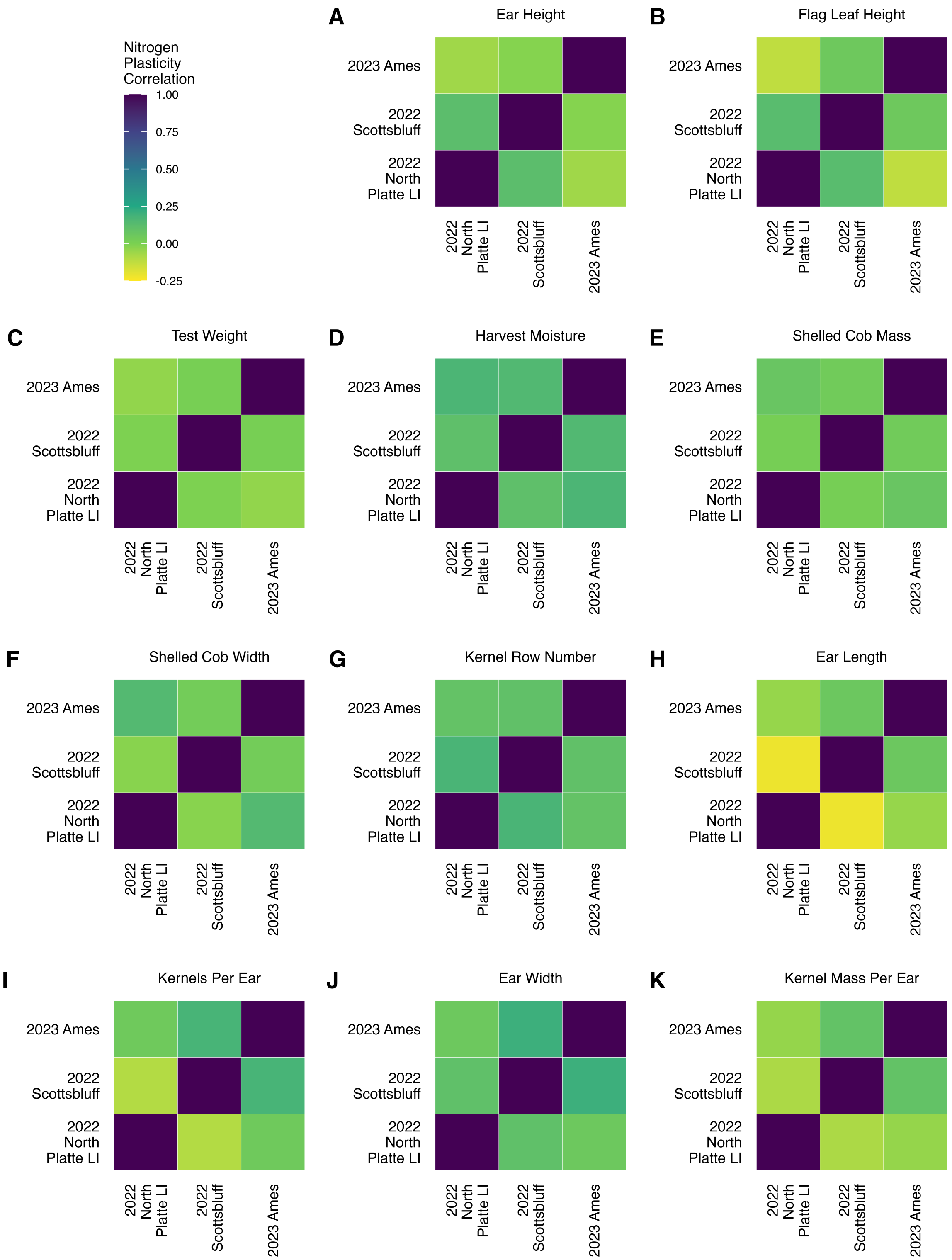

Figure S1. Nitrogen plasticity correlates poorly across location-years for all phenotypes collected in all location-years that were compared. A – K) Correlation of nitrogen plasticity for 11 phenotypes across three location years. Correlations were determined using the Spearman rank correlation of the linear nitrogen plasticity values estimated using data from all hybrids common to both location-years. Only location-years where a population-level positive relationship of yield with nitrogen fertilization was observed are shown.

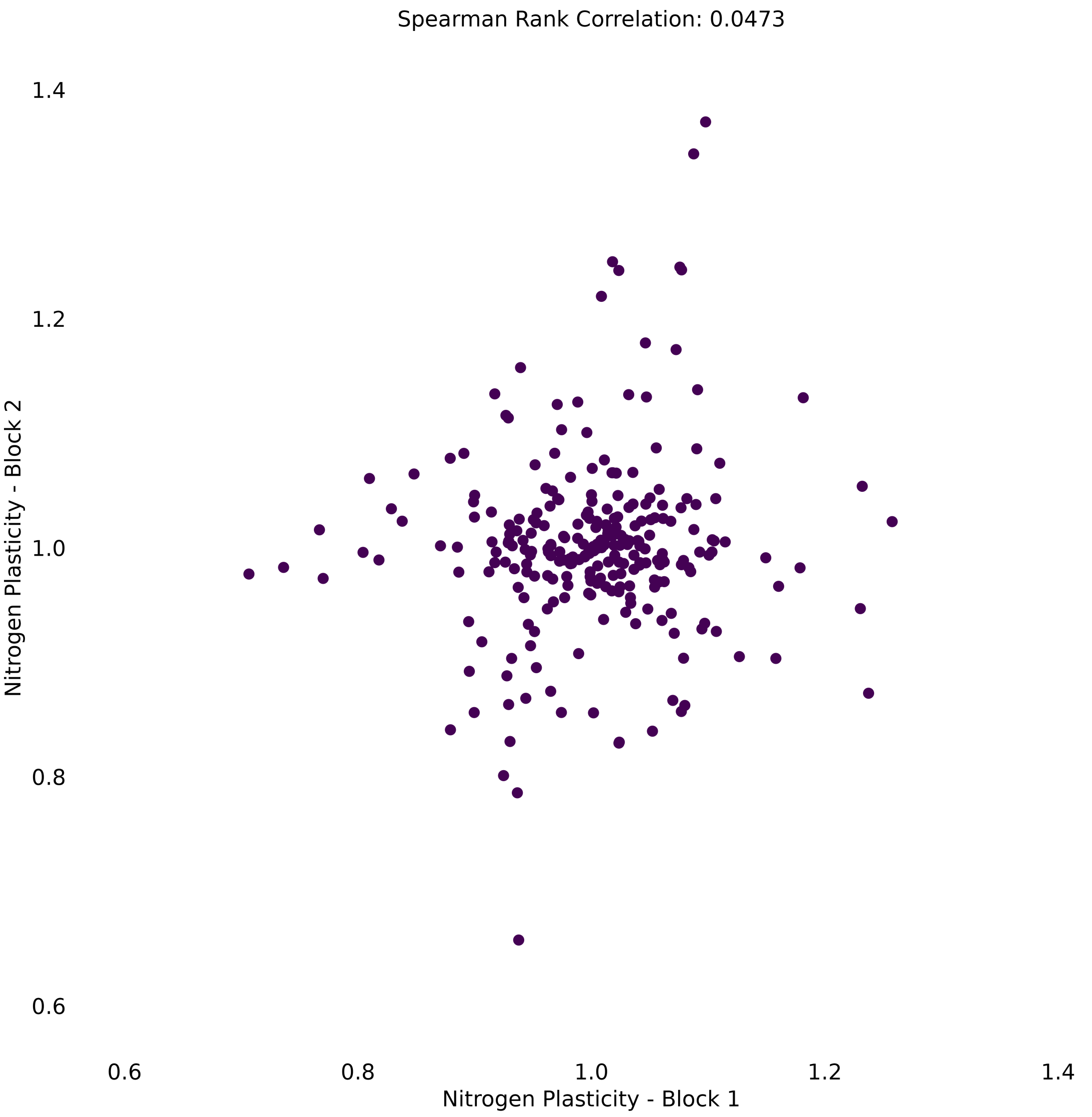

Figure S2. Nitrogen plasticity is poorly repeatable within a location-year. Nitrogen plasticity values were estimated for hybrids using 50% of the data in location-years in which a positive relationship with nitrogen fertilization rate was observed on a population level, compared to the equivalent estimates using the remaining 50% of the data. The data were split by block within an environment.

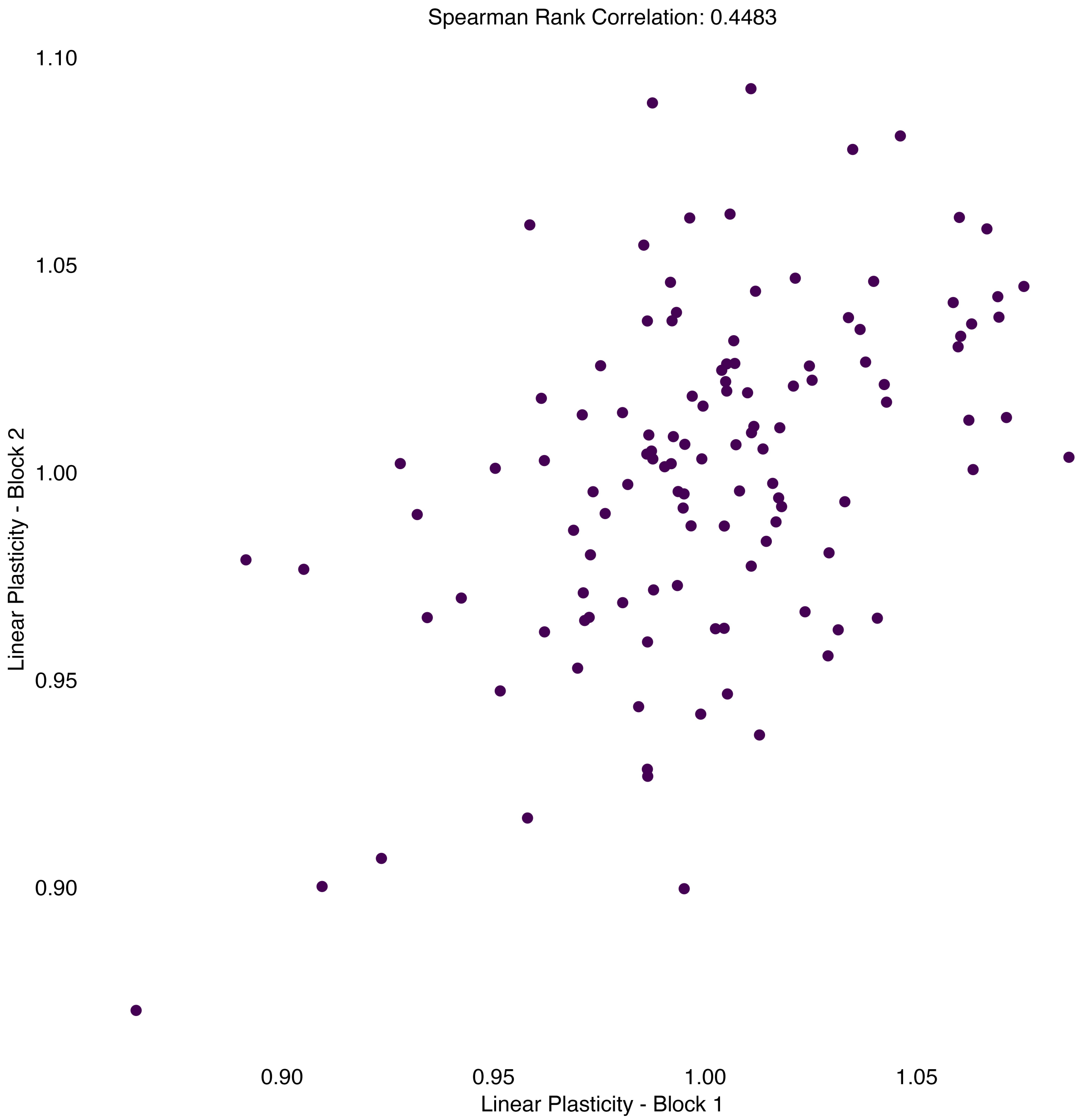

Figure S3. **Linear plasticity values estimated for hybrids across all environments are moderately repeatable.** The data were split by block within an environment.

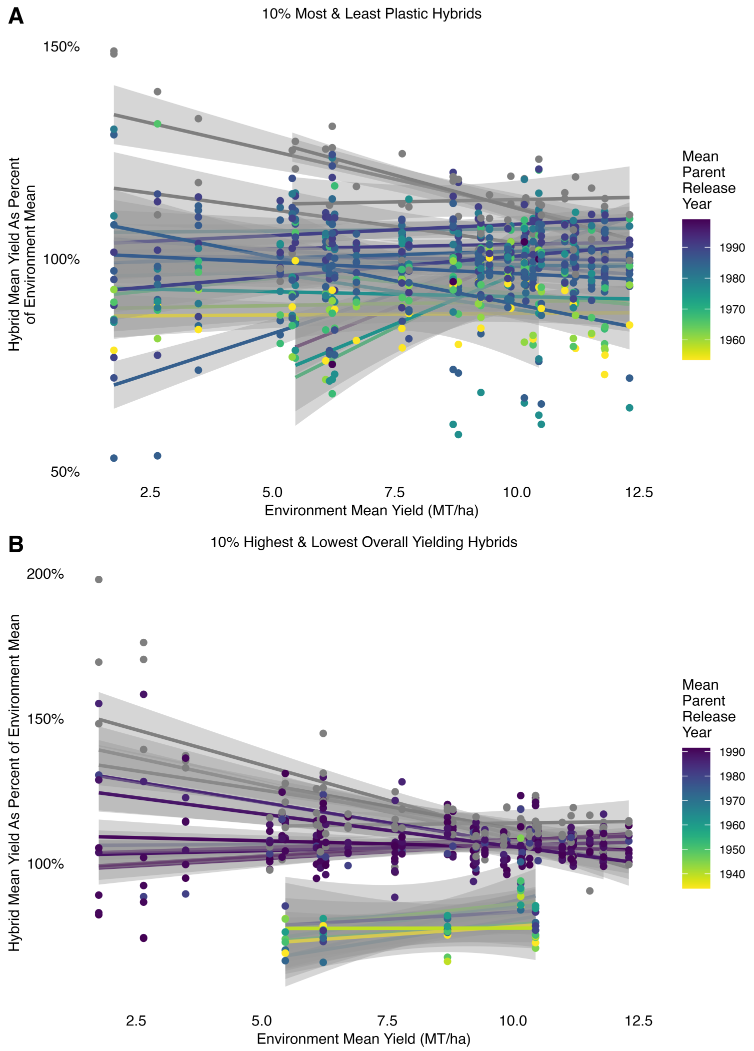

Figure S4. Performance of select hybrids as a percentage of the environment mean. A) The performance of the 10% most plastic and least plastic hybrids across all environments, with performance measured as the hybrid mean yield expressed as a percentage of the environment mean yield. Points represent hybrid means, and lines represent the line of best fit using the lm method and associated 95% confidence intervals. The color legend indicates mean parent release year. Gray indicates commercial hybrids for which both inbred parents are unknown. **B)** The performance of the hybrids with the highest and lowest 10% of yield BLUP values across all environments, with performance measured as the hybrid mean yield expressed as a percentage of the environment mean yield. Points represent hybrid means, and lines represent the line of best fit using the lm method and associated 95% confidence intervals. The color legend indicates mean parent release year. Gray indicates commercial hybrids for which both inbred parents are unknown.

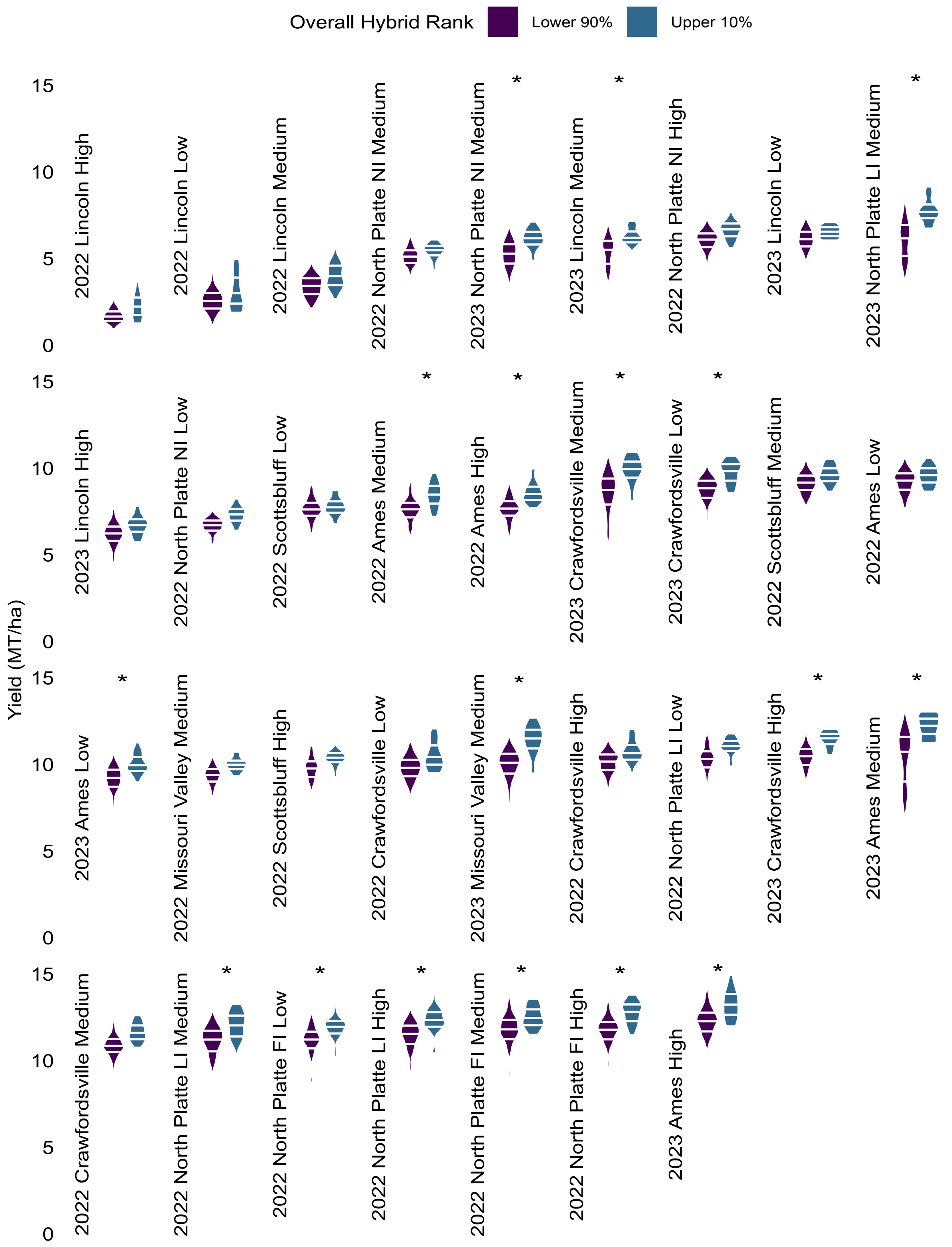

Figure S5. Yield of hybrids ranked in the upper 10% of hybrids for grain yield compared to the yield of all other hybrids by environment. Environments are shown in order of increasing environment mean yield from left to right and top to bottom. Asterisks denote environments where there was a significant difference ($p < 0.05$, Tukey's HSD) between groups within the environment. Horizontal lines within each violin denote the 25th, 50th, and 75th percentiles (Lower 90%: $n = 106 - 147$ plots; Upper 10%: $n = 18 - 22$ plots).

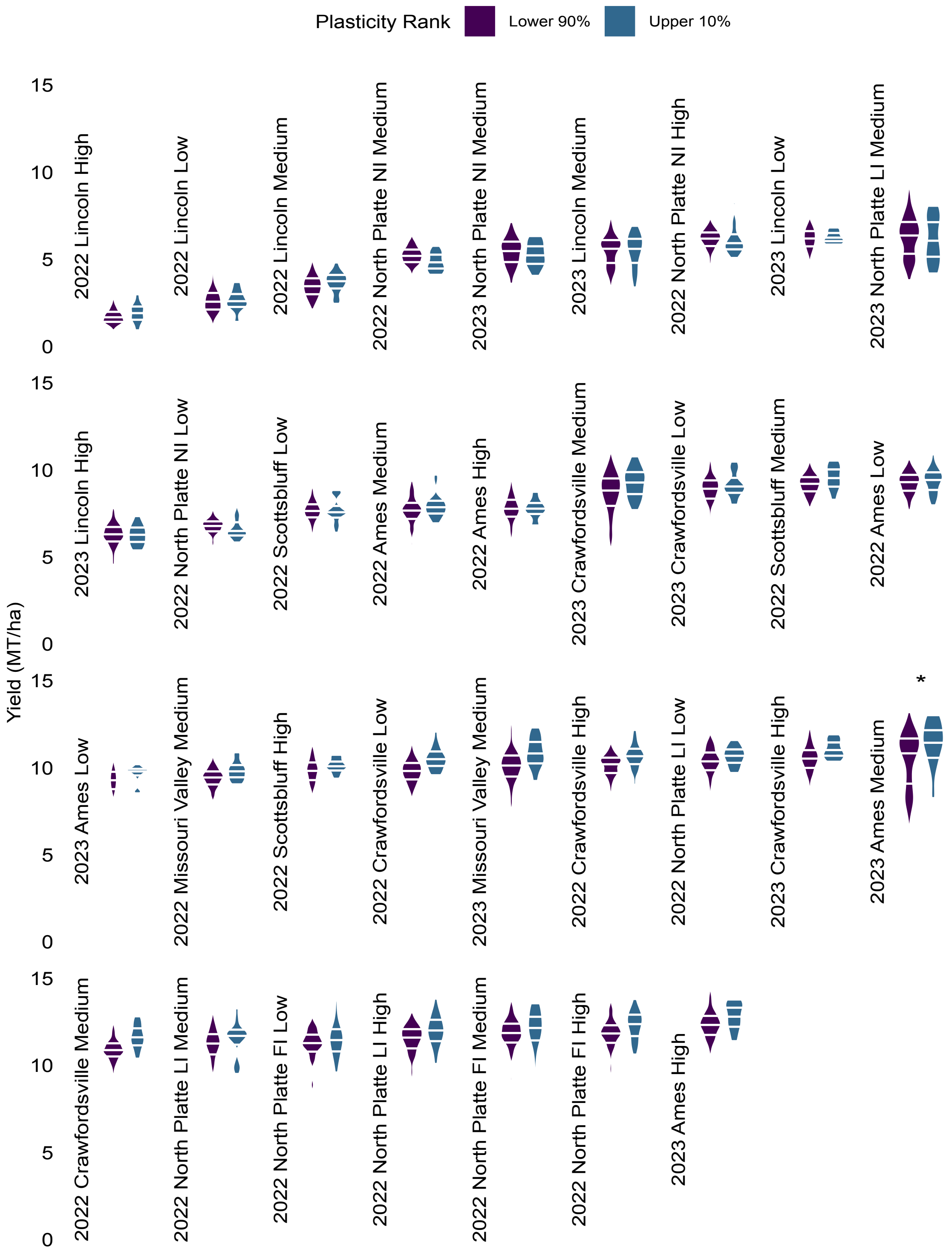

Figure S6. Yield of hybrids ranked in the upper 10% of hybrids for linear plasticity compared to the yield of all other hybrids by environment. Environments are shown in order of increasing environment mean yield from left to right and top to bottom. Asterisks denote environments where there was a significant difference ($p < 0.05$, Tukey's HSD) between groups within the environment. Horizontal lines within each violin denote the 25th, 50th, and 75th percentiles (Lower 90%: $n = 114 - 206$ plots; Upper 10%: $n = 11 - 22$ plots).

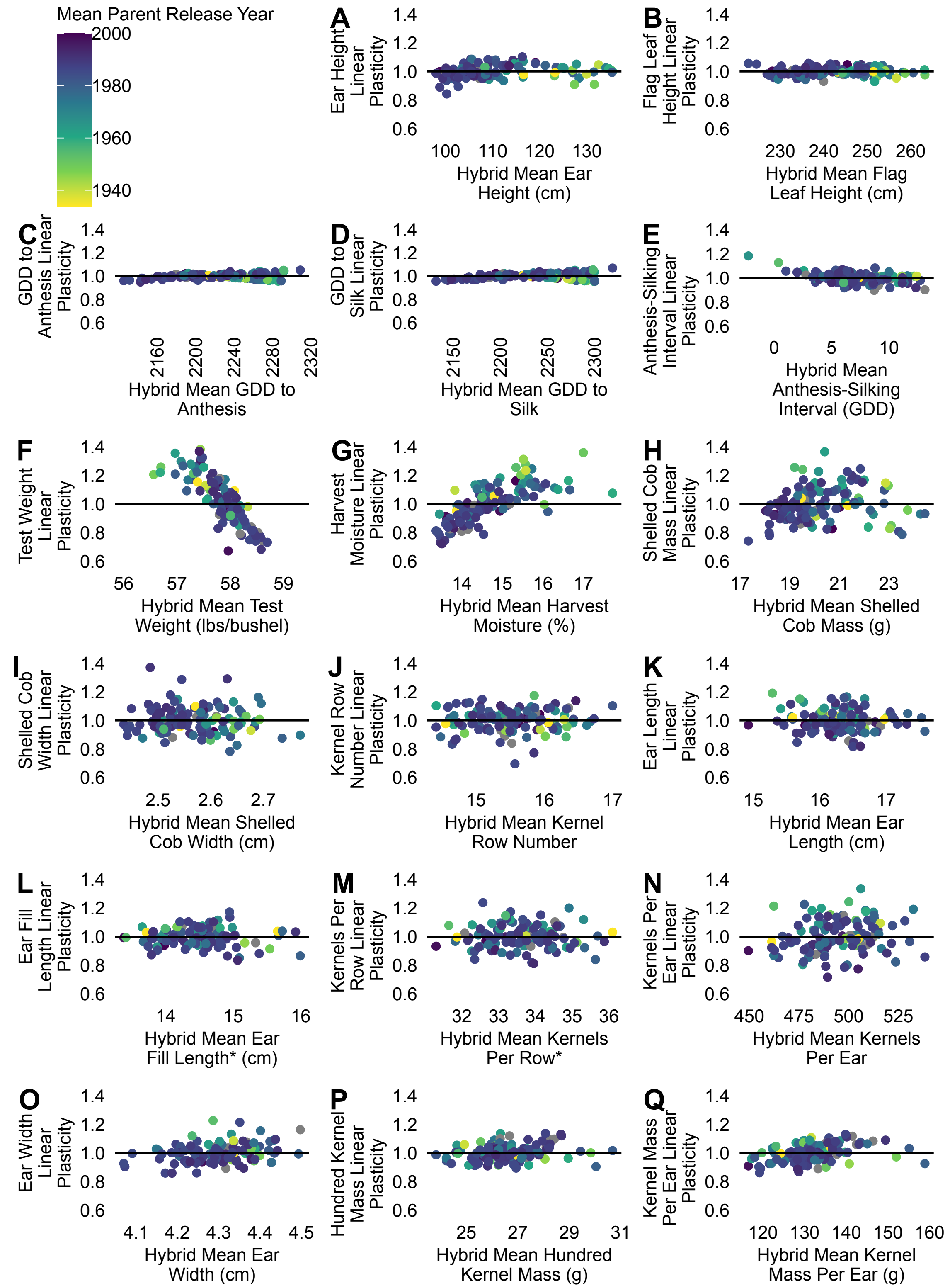

Figure S7. Relationship between Finlay-Wilkinson linear plasticity values across all environments, mean trait value, and mean parent release year is trait-dependent. A – Q) Finlay-Wilkinson linear plasticity values for each hybrid in this study versus their mean trait values across all environments, colored by their mean parent release year for 17 traits. Gray points represent commercial check hybrids for which the inbred parents are unknown. The black horizontal lines indicate a linear plasticity value of 1. GDD indicates growing degree days.

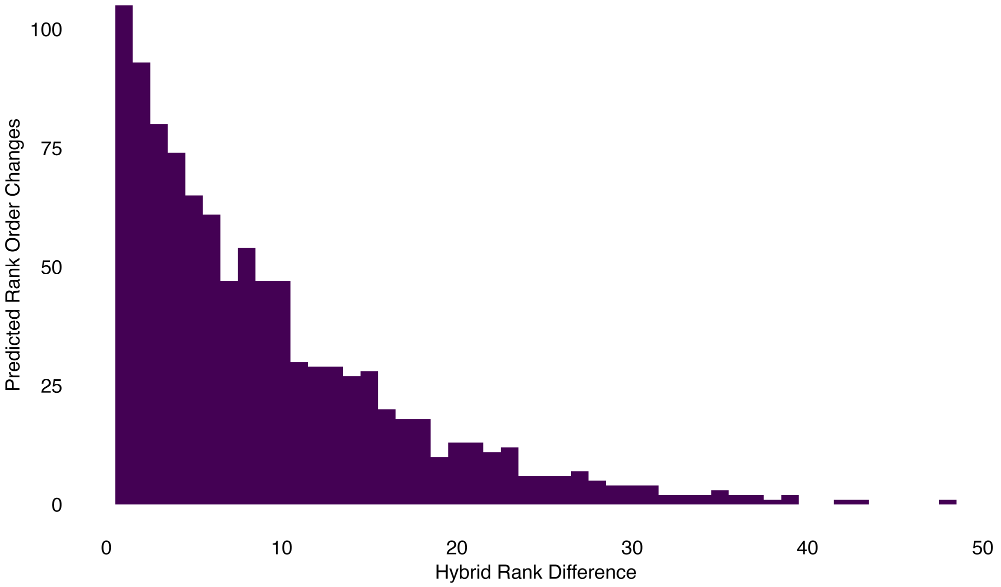

Figure S8. **Distribution of the difference in overall hybrid ranks for the hybrid pairs where Finlay-Wilkinson regression predicted a rank order change in the population of hybrids studied.**

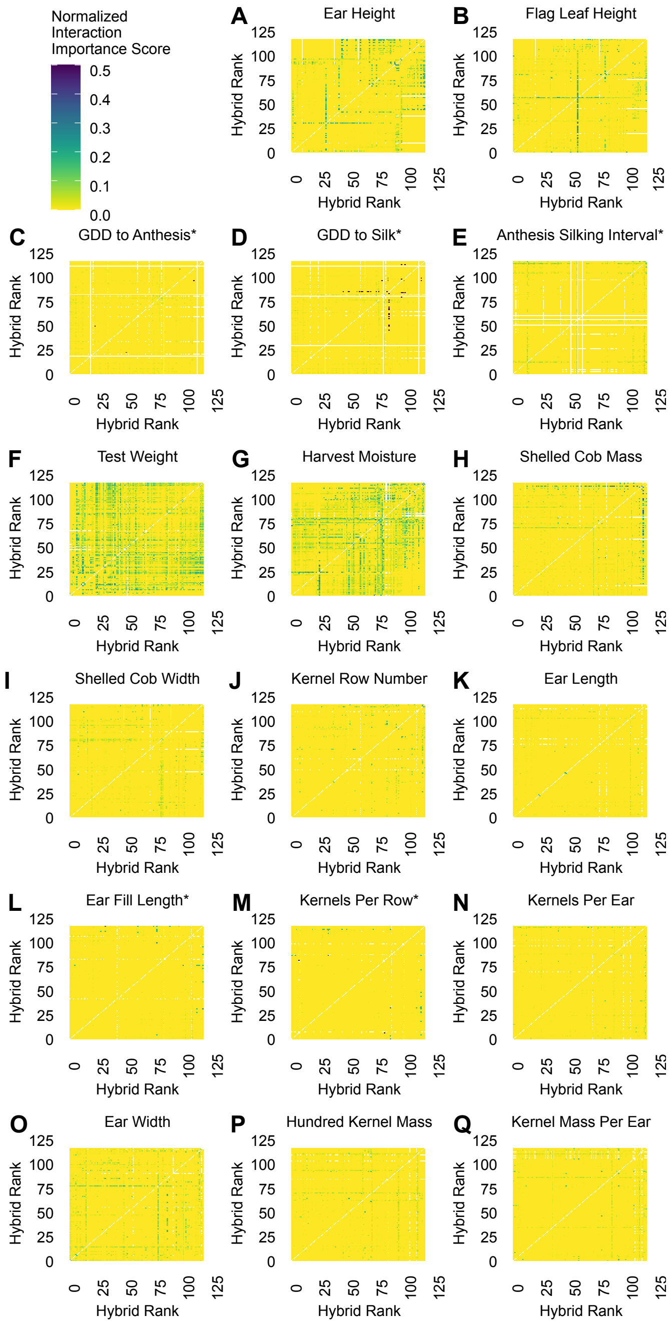

Figure S9. Pattern and level of interaction importance scores varied by trait. A – Q) Incidence matrices indicating the frequency with which a given pair of hybrids exhibited an interaction for the trait between two environments that represents a potentially important change in the selection decision between environments for 17 traits. For each hybrid and environment pair, an interaction received a score of 2 if the rank ordering of the hybrids changed between the two environments and there was a significant difference in yield between the hybrids in both environments. An interaction received a score of 1 if the rank ordering of the hybrids changed between the two environments and there was a significant difference in yield between the hybrids in one of the two environments. An interaction received a score of 0 otherwise. For each hybrid pair, interaction scores were summed across all environment pairs and divided by the total score possible for the hybrid pair based on the number of environments both hybrids were present in. Hybrids are ranked in order of ascending yield BLUP values fitting the environment as a fixed effect. Asterisks denote phenotypes measured only in a subset of environments. GDD indicates growing degree days.

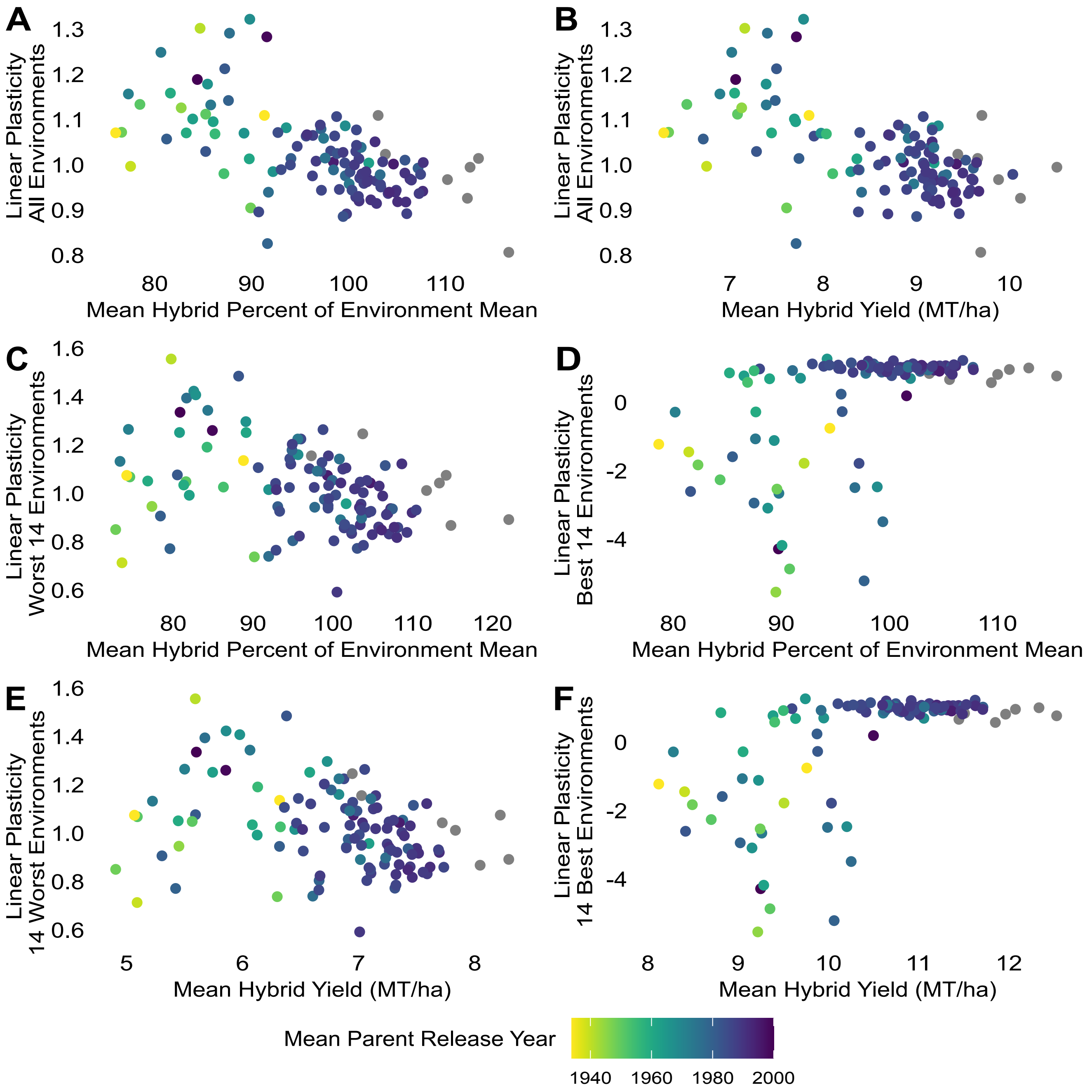

Figure S10. Linear plasticity for yield as a percentage of the environment mean yield shows a relationship with overall performance due to decreased yield response to improved environments. The color legend indicates mean parent release year. Gray points represent commercial check hybrids for which the inbred parents are unknown. **A)** Relationship between linear plasticity and average performance of hybrids relative to the population across all 31 environments used in plasticity analyses (Spearman ρ = -0.61). **B)** Relationship between linear plasticity and average yield of hybrids across all 31 environments used in plasticity analyses (Spearman ρ = -0.56). **C)** Relationship between linear plasticity and average performance of hybrids relative to the population in the 14 environments with the lowest environment mean yields used in plasticity analyses (Spearman ρ = -0.39). **D)** Relationship between linear plasticity and average performance of the hybrid relative to the population in 14 environments with the highest environment mean yields (Spearman ρ = 0.42). **E)** Relationship between linear plasticity and average yield of hybrids across the 14 environments with the lowest environment mean yields used in plasticity analyses (Spearman ρ = -0.36). **F)** Relationship between linear plasticity and average yield of hybrids across the 14 environments with the highest environment mean yields (Spearman ρ = 0.50).
