# Supplementary material for: Assessing the Impact of Yield Plasticity on Hybrid Performance in Maize
Source: Physiol Plant. 2025 May 27;177(3):e70278. doi: 10.1111/ppl.70278 (PMC12117174; doi:10.1111/ppl.70278)
Supplement: Supplementary file 5 — Data S2. [file PPL-177-e70278-s005.pdf]

HIPS README

Jensina Davis

2025-03-19

This is the documentation for the HIPS 2022 & 2023 Dataset.

Variable Definitions And Notes

Each of the following variables includes notes on how the trait was collected and the original data file the data was taken from and any specific notes regarding errors or data cleaning done. Variables are listed by the name used in the final version of the data set. For all quantitative variables, observations an order of magnitude larger than other observations, those appearing as extreme outliers in histograms, and those appearing outside the primary bounds of the distribution for the location were dropped from the data set.

qrCode

- **Definition:** This is the QR code assigned to the plot.
- **Units:** NA
- **Raw Data Sources:** This information for the 2022 Lincoln location hybrids was taken from the sheet 'Combined Dataset' in the file 'Summary of Lincoln Hybrid HIPS 2022 Data.xlsx'. This information for hybrids in the Scottsbluff, Missouri Valley, and North Platte locations in 2022 was taken from the file 'HybridHIPS_plotlevelNIR$_v2.6.csv', and this information for inbreds in these locations and Lincoln in 2022 was taken from the file '2022 Inbred HIPS Ear Data - Turkus Curated 231117.csv'. This information for the Ames and Crawfordsville locations in 2022 was taken from the unique id field in the sheets 'RawData (4-Row)' and 'RawData (2-Row)' in the file 'YTMC_Lisa_Plot_Coordinates_v4.xlsx'. In 2023, this information was taken from the unique id field in the sheets '4-row plots' and '2-row plots' in the file '2023_yield_ICIA_v3.xlsx' for the Ames and Crawfordsville locations. For the North Platte, Lincoln, and Missouri Valley 2023 locations, this information was taken from the sheets 'North Platte Hybrids - Index', 'Lincoln Hybrids - Index', 'Lincoln Inbreds - Index', 'Missouri Valley Hybrids - Index', and 'Missouri Valley Inbreds - Index' in the file 'Summary of HIPS 2023 Maps for Fields Visited by J Schanble Lab.xlsm'.
- **Data Processing Notes:** These were maintained as in the original data, but were converted to all uppercase.

location

- **Definition:** This is the location the plot was grown in. In the case of North Platte 2022, it is treated as 3 separate locations to account for the duplication of range and row numbers across the different irrigation levels.
- **Units:** NA
- **Raw Data Sources:** NA
- **Data Processing Notes:** NA

sublocation

- **Definition:** This is the field within the location the plot was grown in.
- **Units:** NA

- **Raw Data Sources:** In all locations except Ames and Crawfordsville in 2022, this is the same as the `location`. This is information for the Ames and Crawfordsville 2022 locations was determined based on experiment code, which was dependent on the nitrogen level and the field maps.
- **Data Processing Notes:** NA

irrigationProvided

- **Definition:** This is the total amount of water in inches provided to the plot via irrigation over the course of the growing season.
- **Units:** inches
- **Raw Data Sources:** This information for Lincoln, Missouri Valley, Ames, and Crawfordsville sites was taken from personal communications indicating no irrigation was applied to these sites during either growing season. This information for the 2022 Scottsbluff site was taken from personal communications. This information for the 2022 North Platte locations was taken from the file '2022_HIPS_Irrigation_North_Platte.xlsx'. This information for the 2023 North Platte location was taken from the file '2023_Irrigation_North_Platte.xlsx'.
- **Data Processing Notes:** NA

nitrogenTreatment

- **Definition:** This is the categorical level of nitrogen fertilizer the plot was supplied with. Plots denoted as 'Low' received a rate of 75 lbs/acre. Plots denoted as 'Medium' received a rate between 150 and 175 lbs/acre. Plots denoted as 'High' received a rate between 225 and 250 lbs/acre.
- **Units:** NA
- **Raw Data Sources:** This information for the 2022 Lincoln location hybrids was taken from the sheet 'Combined Dataset' in the file 'Summary of Lincoln Hybrid HIPS 2022 Data.xlsx'. This information for hybrids in the Scottsbluff, Missouri Valley, and North Platte locations in 2022 was taken from the file 'HybridHIPS_plotlevelNIR$_v2.6.csv', and this information for inbreds in these locations and Lincoln in 2022 was taken from the file '2022 Inbred HIPS Ear Data - Turkus Curated 231117.csv'. This information for the Ames and Crawfordsville locations in 2022 was based on the experiment codes, which were dependent on the nitrogen treatment. In 2023, this information was taken from the N level field in the sheets '4-row plots' and '2-row plots' in the file '2023_yield_ICIA_v3.xlsx' for the Ames and Crawfordsville locations. For the North Platte, Lincoln, and Missouri Valley 2023 locations, this information was taken from the sheets 'North Platte Hybrids - Index', 'Lincoln Hybrids - Index', 'Lincoln Inbreds - Index', 'Missouri Valley Hybrids - Index', and 'Missouri Valley Inbreds - Index' in the file 'Summary of HIPS 2023 Maps for Fields Visited by J Schanble Lab.xlsm'.
- **Data Processing Notes:** In Scottsbluff, based on the yield and grain protein content data, the labels in the plot maps and the QR codes for high and low nitrogen treatments were flipped. The QR codes are as originally scanned, and this field reflects the correction of this error.

poundsOfNitrogenPerAcre

- **Definition:** This is the rate of nitrogen fertilization the plot received, in pounds per acre.
- **Units:** lbs N/acre
- **Raw Data Sources:** This information for each plot was based on the data documentation for each field and the `nitrogenTreatment` and `location` the plot belongs to. For the Missouri Valley, Ames, and Crawfordsville location in 2022, this information was taken from the 'Basic info' sheet in the file 'YTMC_Lisa_Plot_Coordinates_v4.xlsx'. For the Missouri Valley, Ames, and Crawfordsville locations in 2023, this information was taken from the 'Basic info' sheet in the file '2023_yield_ICIA_v3.xlsx'.

- **Data Processing Notes:** NA

experiment

- **Definition:** This is the experiment code the plot belonged to, corresponding to nitrogen levels within a population (i.e. hybrid or inbred) within a location for the Missouri Valley, Ames, and Crawfordsville locations.
- **Units:** NA
- **Raw Data Sources:** This information was taken from the ‘Basic info’ sheet in the file ‘YTMC_Lisa_Plot_Coordinates_v4.xlsx’ for 2022 and ‘2023_yield_ICIA_v3.xlsx’ for 2023. Experiment codes were not assigned to Lincoln, North Platte, and Scottsbluff locations.
- **Data Processing Notes:** NA

plotLength

- **Definition:** This is the planted length of the plot in feet.
- **Units:** feet
- **Raw Data Sources:** This information for North Platte 2022 was taken from the sheets ‘No Irr Data’, ‘Reduced Irr Data’, and ‘Full Data’ in the file ‘2022_Schnable_HIPS_data_v4.xlsx’. For both 2022 and 2023, this information for the Lincoln location was taken from personal communications with Jon Turkus. This information for Missouri Vallley, Scottsbluff, Ames, and Crawfordsville in 2022 and 2023 was taken from personal communications with Lisa Coffey.
- **Data Processing Notes:** NA

totalStandCount

- **Definition:** This is the number of standing plants in two rows. In the case of hybrids, this is the middle two rows of the four-row plot, and in the case of inbreds, both rows of the two-row plot.
- **Units:** NA
- **Raw Data Sources:** This information (‘Stand’) for Missouri Valley, Crawfordsville, and Ames in 2022 was taken from the sheets ‘RawData (4-Row)’ and ‘RawData (2-Row)’ for hybrids and inbreds, respectively, in the file ‘YTMC_Lisa_Plot_Coordinates_v4.xlsx’. This information for North Platte 2022 was taken from the sheets ‘No Irr Data’, ‘Reduced Irr Data’, and ‘Full Data’ in the file ‘2022_Schnable_HIPS_data_v4.xlsx’ as the sum of the columns ‘R1 Stand’ and ‘R2 Stand’. This data was not collected for the hybrid field at Lincoln in 2022. This information for inbreds in Lincoln in 2022 was taken from the sheet ‘Comparison’ in the file ‘2022 SAM Stand Counts - Summary.xlsx’. This data was not collected in 2023 in Ames, Crawfordsville, or Missouri Valley. This information for hybrids in 2023 in Lincoln was taken from the file ‘2023HYB_HIPS_standing_count.csv’. This information for the 2023 Lincoln inbreds was taken from the file ‘2023SAM_standing_count.csv’. This information for the North Platte 2023 location was taken from sheet ‘Data’ in the file ‘2023 Schnable hips_data_v2.xlsx’.
- **Data Processing Notes:** In the 2023 inbreds, values greater than 50 were visual outliers and converted to missing values.

block

- **Definition:** This is the block number of the plot within a `sublocation` . These values were assigned based on the `location` , `sublocation` , and rep number [not included] associated with a plot.
- **Units:** NA

- **Raw Data Sources:** This information for the Lincoln 2022 location hybrids was taken from the sheet ‘Combined Dataset’ in the file ‘Summary of Lincoln Hybrid HIPS 2022 Data.xlsx’. This information for the hybrids in the 2022 Scottsbluff, Missouri Valley, and North Platte locations was taken from the file ‘HybridHIPS_plotlevelNIR$_v2.6.csv’, and this information for inbreds in these locations and Lincoln in 2022 was taken from the file ‘2022 Inbred HIPS Ear Data - Turkus Curated 231117.csv’. This information for the 2022 Missouri Valley, Ames, and Crawfordsville locations was taken from the sheets ‘RawData (4-Row)’ and ‘RawData (2-Row)’ in the file ‘YTMC_Lisa_Plot_Coordinates_v4.xlsx’. In 2023, this information was taken from the N level field in the sheets ‘4-row plots’ and ‘2-row plots’ in the file ‘2023_yield_ICIA_v3.xlsx’ for the Ames and Crawfordsville locations. For the North Platte, Lincoln, and Missouri Valley 2023 locations, this information was taken from the sheets ‘North Platte Hybrids - Index’, ‘Lincoln Hybrids - Index’, ‘Lincoln Inbreds - Index’, ‘Missouri Valley Hybrids - Index’, and ‘Missouri Valley Inbreds - Index’ in the file ‘Summary of HIPS 2023 Maps for Fields Visited by J Schanble Lab.xlsm’. When the rep number was odd, the `block` number assigned was also odd. The same convention was maintained for even rep numbers.
- **Data Processing Notes:** The rep numbers were misassigned in the QR codes used for ear phenotypes and NIR measurements for the 2022 Missouri Valley hybrids, and have been corrected in the `block` column in the dataset.

row

- **Definition:** This is the row number within a `sublocation` the plot was located in.
- **Units:** NA
- **Raw Data Sources:** This information for the 2022 Lincoln location hybrids was taken from the sheet ‘Combined Dataset’ in the file ‘Summary of Lincoln Hybrid HIPS 2022 Data.xlsx’. This information for hybrids in the 2022 Scottsbluff, Missouri Valley, and North Platte locations was taken from the file ‘HybridHIPS_plotlevelNIR$_v2.6.csv’, and this information for inbreds in these locations and Lincoln in 2022 was taken from the file ‘2022 Inbred HIPS Ear Data - Turkus Curated 231117.csv’. This information for the 2022 Ames and Crawfordsville locations was taken from the sheets ‘RawData (4-Row)’ and ‘RawData (2-Row)’ in the file ‘YTMC_Lisa_Plot_Coordinates_v4.xlsx’. In 2023, this information was taken from the N level field in the sheets ‘4-row plots’ and ‘2-row plots’ in the file ‘2023_yield_ICIA_v3.xlsx’ for the Ames and Crawfordsville locations. For the North Platte, Lincoln, and Missouri Valley 2023 locations, this information was taken from the sheets ‘North Platte Hybrids - Index’, ‘Lincoln Hybrids - Index’, ‘Lincoln Inbreds - Index’, ‘Missouri Valley Hybrids - Index’, and ‘Missouri Valley Inbreds - Index’ in the file ‘Summary of HIPS 2023 Maps for Fields Visited by J Schanble Lab.xlsm’. In some files, this is denoted as ‘Pass’.
- **Data Processing Notes:** NA

range

- **Definition:** This is the range number within a `sublocation` the plot was located in.
- **Units:** NA
- **Raw Data Sources:** This information for the Lincoln location hybrids was taken from the sheet ‘Combined Dataset’ in the file ‘Summary of Lincoln Hybrid HIPS 2022 Data.xlsx’. This information for the hybrids in the Scottsbluff, Missouri Valley, and North Platte locations was taken from the file ‘HybridHIPS_plotlevelNIR$_v2.6.csv’, and this information for inbreds in these locations and Lincoln in 2022 was taken from the file ‘2022 Inbred HIPS Ear Data - Turkus Curated 231117.csv’. This information for the Ames and Crawfordsville locations was taken from the sheets ‘RawData (4-Row)’ and ‘RawData (2-Row)’ in the file ‘YTMC_Lisa_Plot_Coordinates_v4.xlsx’. In 2023, this information was taken from the N level field in the sheets ‘4-row plots’ and ‘2-row plots’ in the file ‘2023_yield_ICIA_v3.xlsx’ for the Ames and Crawfordsville locations. For the North Platte, Lincoln, and Missouri Valley 2023 locations, this information was taken from the sheets ‘North Platte Hybrids - Index’, ‘Lincoln Hybrids - Index’, ‘Lincoln Inbreds -

Index', 'Missouri Valley Hybrids - Index', and 'Missouri Valley Inbreds - Index' in the file 'Summary of HIPS 2023 Maps for Fields Visited by J Schanble Lab.xlsm'.
- **Data Processing Notes:** NA

plotNumber

- **Definition:** This is the number assigned to the plot.
- **Units:** NA
- **Raw Data Sources:** This information for the Lincoln location hybrids was taken from the sheet 'Combined Dataset' in the file 'Summary of Lincoln Hybrid HIPS 2022 Data.xlsx'. This information for the hybrids in the Scottsbluff, Missouri Valley, and North Platte locations was taken from the file 'HybridHIPS_plotlevelNIR$_v2.6.csv', and this information for inbreds in these locations and Lincoln in 2022 was taken from the file '2022 Inbred HIPS Ear Data - Turkus Curated 231117.csv'. This information for the Ames and Crawfordsville locations was taken from the sheets 'RawData (4-Row)' and 'RawData (2-Row)' in the file 'YTMC_Lisa_Plot_Coordinates_v4.xlsx'. In 2023, this information was taken from the N level field in the sheets '4-row plots' and '2-row plots' in the file '2023_yield_ICIA_v3.xlsx' for the Ames and Crawfordsville locations. For the North Platte, Lincoln, and Missouri Valley 2023 locations, this information was taken from the sheets 'North Platte Hybrids - Index', 'Lincoln Hybrids - Index', 'Lincoln Inbreds - Index', 'Missouri Valley Hybrids - Index', and 'Missouri Valley Inbreds - Index' in the file 'Summary of HIPS 2023 Maps for Fields Visited by J Schanble Lab.xlsm'.
- **Data Processing Notes:** In the Scottsbluff hybrids in 2022, it was determined based on correlations between replicates of genotypes that planting of the lowest numbered plots actually started in the SW corner of the field rather than the SE corner as indicated by the field maps. The plot numbers in the `qrCode` were transformed to the plot number assigned to that row and range in the original field maps. In Missouri Valley 2022, 100 was added to hybrid plot numbers in `block` 1 and 200 was added to hybrid plot numbers in `block 2` to de-duplicate the plot numbers within the `location`. In Missouri Valley 2022, 400 was added to the inbred plot numbers in `block 2` to de-duplicate the plot numbers within the `location`. In the Missouri Valley 2023 inbreds, 400 was added to inbred plot numbers in `block 2` to de-duplicate the plot numbers.

genotype

- **Definition:** This is the genotype grown in the plot.
- **Units:** NA
- **Raw Data Sources:** This information for the Lincoln location hybrids was taken from the sheet 'Combined Dataset' in the file 'Summary of Lincoln Hybrid HIPS 2022 Data.xlsx'. This information for the hybrids in the Scottsbluff, Missouri Valley, and North Platte locations was taken from the file 'HybridHIPS_plotlevelNIR$_v2.6.csv', and this information for inbreds in these locations and Lincoln in 2022 was taken from the file '2022 Inbred HIPS Ear Data - Turkus Curated 231117.csv'. This information for the Ames and Crawfordsville locations was taken from the sheets 'RawData (4-Row)' and 'RawData (2-Row)' in the file 'YTMC_Lisa_Plot_Coordinates_v4.xlsx'. In 2023, this information was taken from the N level field in the sheets '4-row plots' and '2-row plots' in the file '2023_yield_ICIA_v3.xlsx' for the Ames and Crawfordsville locations. For the North Platte, Lincoln, and Missouri Valley 2023 locations, this information was taken from the sheets 'North Platte Hybrids - Index', 'Lincoln Hybrids - Index', 'Lincoln Inbreds - Index', 'Missouri Valley Hybrids - Index', and 'Missouri Valley Inbreds - Index' in the file 'Summary of HIPS 2023 Maps for Fields Visited by J Schanble Lab.xlsm'.
- **Data Processing Notes:** All characters in genotype names were converted to uppercase. Some plots' genotypes were listed as 'SOLAR' 1-4, withno note on the hybrid grown in the plot. These plots' genotypes were transformed to NA, with a note on the solar panel in the notes field. Genotype names truncated in

`qrCode` were changed to their full version, genotype names of the form 'COMMERCIAL HYBRID X' were converted to the market name of the commercial hybrid, 'FILLER' was converted to the actual genotype name when known or NA if unknown. Additionally, typos in the genotype names '4N506 X 3IIH!6' and 'PHP02 X PHJ894' were corrected to '4N506 X 3IIH6' and 'PHP02 X PHJ89', respectively.

plantingDate

- **Definition:** This is the date the plot was planted.
- **Units:** NA
- **Raw Data Sources:** This information for each plot was based on the data documentation for each field. For the Missouri Valley, Ames, and Crawfordsville locations, this information was taken from the sheet 'Field Info' in the file YTMC_Lisa_Plot_Coordinates_v4.xlsx' in 2022 and the file '2023_yield_ICIA_v3.xlsx' in 2023. For the Scottsbluff, North Platte, and Lincoln locations, this information was taken from personal communications in both 2022 and 2023.
- **Data Processing Notes:** NA

anthesisDate

- **Definition:** This is the date when 50% of plants in the plot reached anthesis (tasseling).
- **Units:** NA
- **Raw Data Sources:** This information for the 2022 Lincoln location hybrids was taken from the sheet 'Combined Dataset' in the file 'Summary of Lincoln Hybrid HIPS 2022 Data.xlsx'. This information for the 2022 Lincoln location inbreds was taken from the sheet 'SAM' in the file '2022 Digitized Flowering Notes.xlsx'. This information for the 2022 Scottsbluff location hybrids and inbreds was taken from the sheets 'Hybrid_flowering data' and 'Inbred_flowering_data' in the file 'Corn_data_Scottsbluff-2022_rk_11.11.2022'. This information for the 2022 North Platte locations was taken from the sheets 'Full Data', 'Reduced Irr Data', and 'No Irr Data' in the file '2022_Schnable_HIPS_data_v4.xlsx'. This information for the 2023 hybrids in Lincoln was taken from the file 'Hybrid Hips flowering NOTEs 2023 (1).xlsx'. This information for the 2023 inbreds in Lincoln was taken from the file 'UNL Inbreds flowering note 2023 - Ullagaddi Transcribed.csv'. This information for the 2023 North Platte location was taken from the file '2023 Schnable hips_data_v2.xlsx'. This data was not collected at the Missouri Valley, Crawfordsville, and Ames locations in either year.
- **Data Processing Notes:** In the 2023 hybrids, the `anthesisDate` for plot 146 in North Platte was in December 1901, so it was removed as a data entry error.

silkDate

- **Definition:** This is the date when 50% of plants in the plot reached silking.
- **Units:** NA
- **Raw Data Sources:** This information for the 2022 Lincoln location hybrids was taken from the sheet 'Combined Dataset' in the file 'Summary of Lincoln Hybrid HIPS 2022 Data.xlsx'. This information for the 2022 Lincoln location inbreds was taken from the sheet 'SAM' in the file '2022 Digitized Flowering Notes.xlsx'. This information for the 2022 Scottsbluff location hybrids and inbreds was taken from the sheets 'Hybrid_flowering data' and 'Inbred_flowering_data' in the file 'Corn_data_Scottsbluff-2022_rk_11.11.2022'. This information for the 2022 North Platte locations was taken from the sheets 'Full Data', 'Reduced Irr Data', and 'No Irr Data' in the file '2022_Schnable_HIPS_data_v4.xlsx'. This information for the 2023 hybrids in Lincoln was taken from the file 'Hybrid Hips flowering NOTEs 2023 (1).xlsx'. This information for the 2023 inbreds in Lincoln was taken from the file 'UNL Inbreds flowering note 2023 - Ullagaddi Transcribed.csv'. This information for the 2023 North Platte location was taken from the file '2023

Schnable hips_data_v2.xlsx'. This data was not collected at the Missouri Valley, Crawfordsville, and Ames locations in either year.
- **Data Processing Notes:** In the 2023 hybrids, the `silkDate` for plot 349 in North Platte was in January 1900, so it was removed as a data entry error.

daysToAnthesis

- **Definition:** This is the number of days between the `plantingDate` and `anthesisDate`.
- **Units:** days
- **Raw Data Sources:** NA
- **Data Processing Notes:** In the 2023 hybrids, the `anthesisDate` for plot 146 in North Platte was in December 1901, so it was removed as a data entry error. Furthermore, all values less than 50 days were visual outliers and were converted to missing values. In the 2023 inbreds, all values less than 42.5 days were visual outliers and converted to missing values.

daysToSilk

- **Definition:** This is the number of days between the `plantingDate` and `silkDate`.
- **Units:** days
- **Raw Data Sources:** NA
- **Data Processing Notes:** Plots 1397 at North Platte and 1156 at Scottsbluff were visual outliers in scatterplots and were dropped. In the 2022 inbreds, values greater than 110 days were visual outliers and were converted to missing values. In the 2023 hybrids, the `silkDate` for plot 349 in North Platte was in January 1900, so it was removed as a data entry error.

anthesisSilkingInterval

- **Definition:** This is the anthesis-silking interval in days, and is calculated as the difference between the `silkDate` and `anthesisDate` for a plot.
- **Units:** days
- **Raw Data Sources:** NA
- **Data Processing Notes:** Negative values indicate that silking occurred prior to anthesis. Values greater than 20 days were visual outliers in the histogram and were dropped. In the 2022 inbreds, values less than -10 days or greater than 20 days were visual outliers and were converted to missing values. In the 2023 hybrids, plots 146 and 349 in North Platte had data entry errors in either `anthesisDate` or `silkDate` and so `anthesisSilkingInterval` was not calculated. Furthermore, all values greater than 15 days were visual outliers and were converted to missing values. In the 2023 inbreds, values less than -15 or greater than 25 days were visual outliers and converted to missing values.

GDDToAnthesis

- **Definition:** This is the cumulative number of GDDs for the location on between `plantingDate` and `anthesisDate` for the plot.
- **Units:** growing degree days (GDDs)
- **Raw Data Sources:** In-field weather station data for the 2022 growing season was taken from the files in the directory 'weather'. In-field weather station data for the 2023 growing season was taken from the files in the directory '2023/2023 Weather Stations'.
- **Data Processing Notes:** GDDs were calculated using temperatures in Fahrenheit with a crop base temperature of 50 degrees and a crop maximum temperature of 86 degrees. Where there was a weather

station data for the field, the temperatures recorded therein were used to calculate the daily minimum and maximum temperatures and converted from Celsius if needed. In cases where there was missing data or the maximum temperature was above the maximum observed temperature for the nearest town, data was imputed from NASA POWER using the latitude and longitude of the center of the plot. In Crawfordsville 2022, there was no weather station but a G2F field was located nearby. The weather data for Crawfordsville 2022 was accessed from doi:10.25739/3d3g-pe51 (doi:10.25739/3d3g-pe51) for the site IAH1. This data was previously cleaned and imputed using data from NASA. In the 2022 hybrids, values in Lincoln greater than 2250 GDDs, values in North Platte1 less than 1750 GDDs, values in North Platte3 less than 2250 or greater than 3500 GDDs, and values in Scottsbluff less than 1500 GDDs were visual outliers in the histogram and were dropped. In the 2022 inbreds, values greater than 3000 GDDs were visual outliers and converted to missing values. In the 2023 hybrids, values less than 1100 were visual outliers and converted to missing values. In the 2023 inbreds, values less than 1000 GDDs were visual outliers and converted to missing values.

GDDToSilk

- **Definition:** This is the cumulative number of GDDs for the location on between `plantingDate` and `silkDate` for the plot.
- **Units:** growing degree days (GDDs)
- **Raw Data Sources:** In-field weather station data for the 2022 growing season was taken from the files in the directory ‘weather’. In-field weather station data for the 2023 growing season was taken from the files in the directory ‘2023/2023 Weather Stations’.
- **Data Processing Notes:** GDDs were calculated using temperatures in Fahrenheit with a crop base temperature of 50 degrees and a crop maximum temperature of 86 degrees. Where there was a weather station data for the field, the temperatures recorded therein were used to calculate the daily minimum and maximum temperatures and converted from Celsius if needed. In cases where there was missing data or the maximum temperature was above the maximum observed temperature for the nearest town, data was imputed from NASA POWER using the latitude and longitude of the center of the plot. In Crawfordsville 2022, there was no weather station but a G2F field was located nearby. The weather data for Crawfordsville 2022 was accessed from doi:10.25739/3d3g-pe51 (doi:10.25739/3d3g-pe51) for the site IAH1. This data was previously cleaned and imputed using data from NASA. Values greater than 3500 GDDs in the 2022 hybrids were visual outliers in the histogram and were dropped. In the 2022 inbreds, all values greater than 3250 GDDs were visual outliers and were converted to missing values. In the 2023 hybrids, values less than 1100 were visual outliers and converted to missing values. In the 2023 inbreds, values less than 1000 or greater than 2800 GDDs were visual outliers and converted to missing values.

anthesisSilkingIntervalGDD

- **Definition:** This is the cumulative number of GDDs for the location on between `anthesisDate` and `silkDate` for the plot.
- **Units:** growing degree days (GDDs)
- **Raw Data Sources:** Negative values indicate silking occurred prior to anthesis. In-field weather station data for the 2022 growing season was taken from the files in the directory ‘weather’. In-field weather station data for the 2023 growing season was taken from the files in the directory ‘2023/2023 Weather Stations’.
- **Data Processing Notes:** GDDs were calculated using temperatures in Fahrenheit with a crop base temperature of 50 degrees and a crop maximum temperature of 86 degrees. Where there was a weather station data for the field, the temperatures recorded therein were used to calculate the daily minimum and maximum temperatures and converted from Celsius if needed. In cases where there was missing data or the maximum temperature was above the maximum observed temperature for the nearest town, data was

imputed from NASA POWER using the latitude and longitude of the center of the plot. In Crawfordsville 2022, there was no weather station but a G2F field was located nearby. The weather data for Crawfordsville 2022 was accessed from doi:10.25739/3d3g-pe51 (doi:10.25739/3d3g-pe51) for the site IAH1. This data was previously cleaned and imputed using data from NASA. Values greater than 500, values in North Platte1 greater than 250, and values in Scottsbluff greater than 375 were visual outliers and converted to missing values. In the 2022 inbreds, values less than -250 or greater than 750 GDDs were visual outliers and were converted to missing values. In the 2023 hybrids, values less than -50 or greater than 200 were visual outliers and converted to missing values. In the 2023 inbreds, all values less than -500 or greater than 1000 GDDs were visual outliers and converted to missing values.

earHeight

- **Definition:** This is the height in centimeters of the point of attachment to the stalk of the top ear.
- **Units:** cm
- **Raw Data Sources:** This information for the 2022 Lincoln location hybrids was taken from the sheet ‘Combined Dataset’ in the file ‘Summary of Lincoln Hybrid HIPS 2022 Data.xlsx’. For the 2022 Lincoln inbreds, this information was taken from the file ‘230331 SAM Height and Leaf Dimension Data Data - Digitized and Reviewed.xlsx’. For Scottsbluff hybrids, this data was taken from the sheet ‘Hybrid_height data’ from the file ‘Corn_data_Scottsbluff-2022_rk_11.11.2022’ and converted from inches. This information for the North Platte 2022 location was taken from the sheets ‘Full Data’, ‘Reduced Irr Data’, and ‘No Irr Data’ in the file ‘2022_Schnable_HIPS_data_v4.xlsx’ and converted from meters. This information for Missouri Valley 2022 was taken from the file ‘Plant_data_MO_Valley_2022.xlsx’. This information for Ames 2022 was taken from the file ‘Plant_data_Ames_2022.xlsx.’ This information for Crawfordsville 2022 was taken from the file ‘Plant_data_Crawfordsville.xlsx’. This information for North Platte 2023 was taken from the file ‘2023 Schnable hips_data_v2.xlsx’ and converted from meters. This information for Lincoln 2023 hybrids was taken from the file ‘2023HIBHIPS_plat_height.csv’ and converted from feet. This information for Lincoln 2023 inbreds was taken from the file ‘231018 Finalized Height Data from Lopez-Corona - Additional info added by Turkus.xlsx’ and converted from feet. This information for the 2023 Missouri Valley, Ames, and Crawfordsville was taken from the sheets ‘4-row plots’ and ‘2-row plots’ in the file ‘2023_yield_ICIA_v3.xlsx’.
- **Data Processing Notes:** In Lincoln, this is the average of measurements from two representative plants in the plot. In all other locations, it measured from a single representative plant in the plot. All measurements from plants noted as stunted or without a silk were marked as missing data prior to calculation. Ear heights less than 35 cm were visual outliers in the histogram and were dropped. Plots 5244, 5283, 5141 in Lincoln were dropped as they appeared to be an order of magnitude too low. In the 2022 inbreds, values in Crawfordsville less than 12 cm or greater than 175 cm were visual outliers and were converted to missing values. In the 2023 hybrids, all values greater than 225 cm and values in North Platte less than 80 cm were visual outliers and were converted to missing values. In the 2023 inbreds, values less than 12 or greater than 200 cm were visual outliers and converted to missing values.

flagLeafHeight

- **Definition:** This is the height in centimeters of the point of attachment to the stalk of the flag leaf.
- **Units:** cm
- **Raw Data Sources:** This information for the 2022 Lincoln location hybrids was taken from the sheet ‘Combined Dataset’ in the file ‘Summary of Lincoln Hybrid HIPS 2022 Data.xlsx’. For the 2022 Lincoln inbreds, this information was taken from the file ‘230331 SAM Height and Leaf Dimension Data Data - Digitized and Reviewed.xlsx’. For Scottsbluff hybrids, this data was taken from the sheet ‘Hybrid_height data’ from the file ‘Corn_data_Scottsbluff-2022_rk_11.11.2022’ and converted from inches. This

information for the North Platte 2022 location was taken from the sheets ‘Full Data’, ‘Reduced Irr Data’, and ‘No Irr Data’ in the file ‘2022_Schnable_HIPS_data_v4.xlsx’ and converted from meters. This information for Missouri Valley 2022 was taken from the file ‘Plant_data_MO_Valley_2022.xlsx’. This information for Ames 2022 was taken from the file ‘Plant_data_Ames_2022.xlsx.’ This information for Crawfordsville 2022 was taken from the file ‘Plant_data_Crawfordsville.xlsx’. This information for North Platte 2023 was taken from the file ‘2023 Schnable hips_data_v2.xlsx’ and converted from meters. This information for Lincoln 2023 hybrids was taken from the file ‘2023HIBHIPS_plat_height.csv’ and converted from feet. This information for Lincoln 2023 inbreds was taken from the file ‘231018 Finalized Height Data from Lopez-Corona - Additional info added by Turkus.xlsx’ and converted from feet. This information for the 2023 Missouri Valley, Ames, and Crawfordsville was taken from the sheets ‘4-row plots’ and ‘2-row plots’ in the file ‘2023_yield_ICIA_v3.xlsx’.

- **Data Processing Notes:** In Lincoln, this is the average of measurements from two representative plants in the plot. In all other locations, it measured from a single representative plant in the plot. All measurements from plants noted as stunted or without a silk were marked as missing data prior to calculation. Plot 1322 at Scottsbluff was dropped due to having a value 60 cm (~1.9 feet) greater than all other observations. Flag leaf heights less than 50 cm were visual outliers in the histogram and were dropped. In the 2022 inbreds, all values greater than 250 cm, values in Ames less than 45 cm, values in Lincoln less than 50 cm, and values in Missouri Valley less than 50 or greater than 225 cm were visual outliers and were converted to missing values. In the 2023 hybrids, all values less than 100 or greater than 350 cm were visual outliers and were converted to missing values. In the 2023 inbreds, values less than 50 or greater than 500 cm were visual outliers and converted to missing values.

plantDensity

- **Definition:** This is density of the plants per acre in the plot.
- **Units:** plants/acre
- **Raw Data Sources:** In the case of the 2023 Missouri Valley, Ames, and Crawfordsville locations, no stand count was collected, so the Population (acre) column in the sheets ‘4-row plots’ and ‘2-row plots’ in the file ‘2023_yield_ICIA_v3.xlsx’ was used, which was based on the number of seeds planted.
- **Data Processing Notes:** This is calculated as the mean number of plants per row (i.e., one-half of `totalStandCount`) scaled to be equivalent to 1/1000th of an acre (a `plotLength` of 17.5 feet with an assumed spacing of 30”) and multiplied by 1000. In the 2023 hybrids, values less than 20,000 or greater than 50,000 were visual outliers and were converted to missing values.

combineYield

- **Definition:** This is the weight, in pounds, of the grain harvested from the inner two rows of the plot by the combine.
- **Units:** lbs
- **Raw Data Sources:** This data was only collected for hybrid plots. This information for the 2022 Lincoln location was taken from the sheet ‘Combined Dataset’ in the file ‘Summary of Lincoln Hybrid HIPS 2022 Data.xlsx’. For the Scottsbluff location, this information was taken from the file ‘Dipak Corn22_HM.xlsx’. This information for the 2022 North Platte location was taken from the sheets ‘Full Data’, ‘Reduced Irr Data’, and ‘No Irr Data’ in the file ‘2022_Schnable_HIPS_data_v4.xlsx.’ This information for the Missouri Valley, Crawfordsville, and Ames locations was taken from the sheet ‘RawData (4-Row)’ in the file ‘YTMC_Lisa_Plot_Coordinates_v4.xlsx’. This information for the 2023 North Platte location was taken from the file ‘2023 Schnable hips_data_v2.xlsx’. This information for the 2023 Lincoln location was taken from the file ‘231127 Hybrid HIPS Lincoln - Combine Harvest Data - Turkus Curated.csv’. This information for the

2023 Missouri Valley, Ames, and Crawfordsville locations was taken from the sheet ‘4-row plots’ in the file ‘2023_yield_ICIA_v3.xlsx’.

- **Data Processing Notes:** All observations with any of the following values in combineNotes were dropped: “Left row ran over”, “Right row gone”, “Run over”, “Clog”, “Animal damage”, “Animal damage, not enough grain for accurate moisture”, “One row”, “Same as last”, “11/9/2022, 11:45:04 AM, Tare Warning, Test Weight: 5.75”, “Plot lost to belt issue”. Plot 250 in North Platte1 was also dropped, as it had a weight approximately twice all other values and appeared that 4 rows, instead of two, were potentially harvested. Plots 1094, 1095, 1265, and 1435 in Scottsbluff were dropped as they were damaged due to solar panel removal. In Lincoln 2023, some plots accidentally had ears from the center row harvested during hand harvesting. When this was noted, these values were converted to missing data.

yieldPerAcre

- **Definition:** This is the yield, in 56-lb bushels of grain at 15.5% moisture per acre.
- **Units:** bushels/acre
- **Raw Data Sources:** NA
- **Data Processing Notes:** This value was calculated from the `combineYield`, `combineMoisture`, and `plotLength` values for the plot. This calculation assumes a 30” row spacing. In Lincoln 2023, some plots accidentally had ears from the center row harvested during hand harvesting. When this was noted, these values were converted to missing data. Plots 1094, 1095, 1265, and 1435 in Scottsbluff were dropped as they were damaged due to solar panel removal.

combineMoisture

- **Definition:** This is the moisture content, in percent, on a wet (fresh-weight) basis, measured by the combine at the time of harvesting the plot.
- **Units:** percent
- **Raw Data Sources:** This data was only collected for hybrid plots. This information for the 2022 Lincoln location was taken from the sheet ‘Combined Dataset’ in the file ‘Summary of Lincoln Hybrid HIPS 2022 Data.xlsx’. For the Scottsbluff location, this information was taken from the file ‘Dipak Corn22_HM.xlsx’. This information for the 2022 North Platte location was taken from the sheets ‘Full Data’, ‘Reduced Irr Data’, and ‘No Irr Data’ in the file ‘2022_Schnable_HIPS_data_v4.xlsx.’ This information for the Missouri Valley, Crawfordsville, and Ames locations was taken from the sheet ‘RawData (4-Row)’ in the file ‘YTMC_Lisa_Plot_Coordinates_v4.xlsx’. This information for the 2023 North Platte location was taken from the file ‘2023 Schnable hips_data_v2.xlsx’. This information for the 2023 Lincoln location was taken from the file ‘231127 Hybrid HIPS Lincoln - Combine Harvest Data - Turkus Curated.csv’. This information for the 2023 Missouri Valley, Ames, and Crawfordsville locations was taken from the sheet ‘4-row plots’ in the file ‘2023_yield_ICIA_v3.xlsx’.
- **Data Processing Notes:** All observations equal to zero or with any of the following values in combineNotes were dropped: “Left row ran over”, “Right row gone”, “Run over”, “Clog”, “Animal damage”, “Animal damage, not enough grain for accurate moisture”, “One row”, “Same as last”, “11/9/2022, 11:45:04 AM, Tare Warning, Test Weight: 5.75”, “Plot lost to belt issue”, “Water stress, not enough grain for accurate moisture”, “Not enough grain for accurate moisture”. Plot 250 in North Platte1 was also dropped, as it had a weight approximately twice all other values and appeared that 4 rows, instead of two, were potentially harvested. In the 2023 hybrids, all values less than 10 percent were visual outliers and were converted to missing values.

combineTestWeight

- **Definition:** This is the test weight, e.g. the weight in pounds of a volumetric bushel (1.24 cubic feet), of the grain harvested from the plot by the combine.
- **Units:** lbs
- **Raw Data Sources:** This data was only collected for hybrid plots. This information for the 2022 Lincoln location was taken from the sheet ‘Combined Dataset’ in the file ‘Summary of Lincoln Hybrid HIPS 2022 Data.xlsx’. For the Scottsbluff location, this information was taken from the file ‘Dipak Corn22_HM.xlsx’. This information for the 2022 North Platte location was taken from the sheets ‘Full Data’, ‘Reduced Irr Data’, and ‘No Irr Data’ in the file ‘2022_Schnable_HIPS_data_v4.xlsx.’ This information for the Missouri Valley, Crawfordsville, and Ames locations was taken from the sheet ‘RawData (4-Row)’ in the file ‘YTMC_Lisa_Plot_Coordinates_v4.xlsx’. This information for the 2023 North Platte location was taken from the file ‘2023 Schnable hips_data_v2.xlsx’. This information for the 2023 Lincoln location was taken from the file ‘231127 Hybrid HIPS Lincoln - Combine Harvest Data - Turkus Curated.csv’. This information for the 2023 Missouri Valley, Ames, and Crawfordsville locations was taken from the sheet ‘4-row plots’ in the file ‘2023_yield_ICIA_v3.xlsx’.
- **Data Processing Notes:** All observations equal to zero or with any of the following values in combineNotes were dropped: “Left row ran over”, “Right row gone”, “Run over”, “Clog”, “Animal damage”, “Animal damage, not enough grain for accurate moisture”, “One row”, “Same as last”, “11/9/2022, 11:45:04 AM, Tare Warning, Test Weight: 5.75”, “Plot lost to belt issue”. Plot 250 in North Platte1 was also dropped, as it had a weight approximately twice all other values and appeared that 4 rows, instead of two, were potentially harvested. For the Missouri Valley, Ames, and Crawfordsville locations, this data was taken from the file ‘YTMC_Lisa_Plot_Coordinates_v4.xlsx’. In the 2023 hybrids, all values less than 45 pounds were visual outliers and were converted to missing values.

earLength

- **Definition:** This is the mean length of the cob after shelling (i.e. with the kernels removed) of one ear from the plot.
- **Units:** cm
- **Raw Data Sources:** This information for hybrids at the 2022 Scottsbluff, North Platte, Lincoln, and Missouri Valley locations was taken from the file ‘plotleveleardata_v2.csv’. This information for the hybrid 2022 Ames and Crawfordsville locations was taken from the files in the folder ‘5 Cob Traits Station’ and converted from millimeters to centimeters. This information for the 2022 inbreds at the Lincoln and Missouri Valley locations was taken from the file ‘2022 Inbred HIPS Ear Data - Turkus Curated 231117.csv’ and converted from millimeters. This information for the inbred 2022 Ames and Crawfordsville locations was taken from the file ‘5_cob_trait_INBRED_2022_compiled_v2.xlsx’. This information for the hybrids at the 2023 North Platte, Lincoln, and Missouri Valley locations was taken from the file ‘2023_Hyb_HIPS_LNK_MV_NP_Final_KL_Curated.xlsx’. This information for the hybrid 2023 Ames and Crawfordsville locations was taken from the file ‘5_cob_Traits_HYBRID_2023_compiled.xlsx’. This information for the inbred 2023 Lincoln and Missouri Valley locations was taken from the file ‘2023_Inbred_Hips_Ear_Phenotyping_MV_LNK_Final_KL_Curated.xlsx’.
- **Data Processing Notes:** Plot 426 in North Platte1 was dropped as all underlying observations were an order of magnitude larger than all other observations. For Ames and Crawfordsville locations, when the ear had severe bending, a string was used to measure the length and this is denoted in the notes field. In the 2022 inbreds, all values greater than 30 cm were visual outliers and converted to missing values. Furthermore, values in Ames greather than 25 cm, values in Crawfordsville less than 5 or greater than 21 cm, and values in Missouri Valley greater than 20 cm were visual outliers and converted to missing values. In the 2023 hybrids, all values greater than 22.5 cm, values in Crawfordsville less than 12.5 cm, and values

in North Platte less than 10 or greater than 20 cm were visual outliers and converted to missing values. In the 2023 inbreds, values in Lincoln greater than 17.5 cm were visual outliers and converted to missing values.

earFillLength

- **Definition:** This is the mean length filled with kernels on one ear from the plot in centimeters prior to shelling (i.e. with the kernels attached to the cob).
- **Units:** cm
- **Raw Data Sources:** This information for hybrids at the 2022 Scottsbluff, North Platte, Lincoln, and Missouri Valley locations was taken from the file 'plotleveleardata_v2.csv'. This information was not collected for Ames or Crawfordsville locations. This information for the 2022 inbreds at the Lincoln and Missouri Valley locations was taken from the file '2022 Inbred HIPS Ear Data - Turkus Curated 231117.csv' and converted from millimeters. This information for the hybrids at the 2023 North Platte, Lincoln, and Missouri Valley locations was taken from the file '2023_Hyb_HIPS_LNK_MV_NP_Final_KL_Curated.xlsx'. This information for the inbred 2023 Lincoln and Missouri Valley locations was taken from the file '2023_Inbred_Hips_Ear_Phenotyping_MV_LNK_Final_KL_Curated.xlsx'.
- **Data Processing Notes:** Plot 426 in North Platte1 was dropped as all underlying observations were an order of magnitude larger than all other observations, and the mean for plots 879 and 837 in North Platte2 was calculated from 3 (rather than 4) ears due to the observation for the fourth ear being an order of magnitude larger than all other observations. This data was initially not collected for ears phenotyped by the UNL team (North Platte, Scottsbluff, Lincoln, Missouri Valley), but began approximately one-third of the way through phenotyping the first hybrid location in 2022, Missouri Valley. In the 2022 inbreds, values in Lincoln less than 2.5 or greater than 17 cm, values in Missouri Valley greater than 20 cm, and values in Scottsbluff greater than 18 cm were visual outliers and were converted to missing values. In the 2023 inbreds, values in Lincoln less than 3.75 cm wer visual outliers and converted to missing values.

earWidth

- **Definition:** This is the mean width of one ear from the plot in centimeters prior to shelling (i.e. with the kernels attached to the cob).
- **Units:** cm
- **Raw Data Sources:** This information for hybrids at the 2022 Scottsbluff, North Platte, Lincoln, and Missouri Valley locations was taken from the file 'plotleveleardata_v2.csv'. This information for the hybrid 2022 Ames and Crawfordsville locations was taken from the files in the folder '3 Ear Traits Station' and converted from millimeters to centimeters. This information for the 2022 inbreds at the Lincoln and Missouri Valley locations was taken from the file '2022 Inbred HIPS Ear Data - Turkus Curated 231117.csv' and converted from millimeters. This information for the inbred 2022 Ames and Crawfordsville locations was taken from the file '3_ear_trait_INBRED_2022_compiled_v2.xlsx'. This information for the hybrids at the 2023 North Platte, Lincoln, and Missouri Valley locations was taken from the file '2023_Hyb_HIPS_LNK_MV_NP_Final_KL_Curated.xlsx'. This information for the hybrid 2023 Ames and Crawfordsville locations was taken from the file '3_ear_Traits_HYBRID_2023_compiled.xlsx'. This information for the inbred 2023 Lincoln and Missouri Valley locations was taken from the file '2023_Inbred_Hips_Ear_Phenotyping_MV_LNK_Final_KL_Curated.xlsx'.
- **Data Processing Notes:** Plot 426 in North Platte1 was dropped as all underlying observations were an order of magnitude larger than all other observations. Plot 6129 at Lincoln and plot 1585236 were calculated using 3 rather than 4 ears as the remaining observation was an order of magnitude larger than the other observations. In the 2022 inbreds, Lincoln plot 1115 was calculated as the mean of 4 ears rather than 6 as the remaining observations appeared to be data entry errors. In the 2022 inbreds, values greater

than 10 cm were visual outliers and were converted to missing values. Furthermore, values in Ames and Crawfordsville greater than 5.5 cm, values in Lincoln less than 2 or greater than 4.25 cm, and values in Missouri Vallley greater than 5 cm were visual outliers and were converted to missing values. In the 2023 hybrids, all values less than 3.5 cm were visual outliers and converted to missing values. In the 2023 inbreds, values in Missouri Valley less than 2.5 cm were visual outliers and converted to missing values.

shelledCobWidth

- **Definition:** This is the mean width of one cob from the plot in centimeters after shelling (i.e. with the kernels removed from the cob).
- **Units:** cm
- **Raw Data Sources:** This information for hybrids at the 2022 Scottsbluff, North Platte, Lincoln, and Missouri Valley locations was taken from the file 'plotleveleardata_v2.csv'. This information for the hybrid 2022 Ames and Crawfordsville locations was taken from the files in the folder '5 Cob Traits Station' and converted from millimeters to centimeters. This information for the 2022 inbreds at the Lincoln and Missouri Valley locations was taken from the file '2022 Inbred HIPS Ear Data - Turkus Curated 231117.csv' and converted from millimeters. This information for the inbred 2022 Ames and Crawfordsville locations was taken from the file '5_cob_trait_INBRED_2022_compiled_v2.xlsx'. This information for the hybrids at the 2023 North Platte, Lincoln, and Missouri Valley locations was taken from the file '2023_Hyb_HIPS_LNK_MV_NP_Final_KL_Curated.xlsx'. This information for the hybrid 2023 Ames and Crawfordsville locations was taken from the file '5_cob_Traits_HYBRID_2023_compiled.xlsx'. This information for the inbred 2023 Lincoln and Missouri Valley locations was taken from the file '2023_Inbred_Hips_Ear_Phenotyping_MV_LNK_Final_KL_Curated.xlsx'.
- **Data Processing Notes:** Plot 426 in North Platte1 was dropped as all underlying observations were an order of magnitude larger than all other observations. Observations where `shelledCobWidth` was greater than `earWidth` were dropped. In the 2022 inbreds, Lincoln plot 1041 was calculated as the mean of 5 ears rather than 6 as the remaining observation appeared to be a data entry error. Furthermore, values in Crawfordsville less than 1 cm, values in Lincoln less than 0.75 or greater than 3 cm, and values in Scottsbluff less than 1.25 or greater than 3.5 cm were visual outliers and were converted to missing values. In the 2023 hybrids, all values less than 2 or greater than 3.25 cm were visual outliers and converted to missing values. In the 2023 inbreds, values in Lincoln less than 1.75 cm were visual outliers and converted to missing values.

kernelsPerRow

- **Definition:** This is the mean number of kernels in each kernel row.
- **Units:** NA
- **Raw Data Sources:** This information for hybrids at the 2022 Scottsbluff, North Platte, Lincoln, and Missouri Valley locations was taken from the file 'plotleveleardata_v2.csv'. This information was not collected for Ames or Crawfordsville locations. This information for the 2022 inbreds at the Lincoln and Missouri Valley locations was taken from the file '2022 Inbred HIPS Ear Data - Turkus Curated 231117.csv'. This information for the hybrids at the 2023 North Platte, Lincoln, and Missouri Valley locations was taken from the file '2023_Hyb_HIPS_LNK_MV_NP_Final_KL_Curated.xlsx'. This information for the inbred 2023 Lincoln and Missouri Valley locations was taken from the file '2023_Inbred_Hips_Ear_Phenotyping_MV_LNK_Final_KL_Curated.xlsx'.
- **Data Processing Notes:** Plot 4209 at Lincoln was calculated using 3 ears rather than 4 as the remaining observation was an order of magnitude larger than the others. In the 2022 inbreds, all values greater than 40 and values in Lincoln greater than 37 were visual outliers and were converted to missing values. In the

2023 hybrids, all values less than 20 were visual outliers and converted to missing values. In the 2023 inbreds, values in Lincoln greater than 32.5 were visual outliers and converted to missing values.

kernelRowNumber

- **Definition:** This is the mean number of kernel rows, i.e. the number of kernels around the circumference of the ear at the middle of the ear.
- **Units:** NA
- **Raw Data Sources:** This information for hybrids at the 2022 Scottsbluff, North Platte, Lincoln, and Missouri Valley locations was taken from the file ‘plotleveleardata_v2.csv’. This information for the hybrid 2022 Ames and Crawfordsville locations was taken from the files in the folder ‘2 KRN and Ear Documentation’ This information for the 2022 inbreds at the Lincoln and Missouri Valley locations was taken from the file ‘2022 Inbred HIPS Ear Data - Turkus Curated 231117.csv’. This information for the inbred 2022 Ames and Crawfordsville locations was taken from the file ‘2_KRN_trait_INBRED_2022_compiled_v2.xlsx’. This information for the hybrids at the 2023 North Platte, Lincoln, and Missouri Valley locations was taken from the file ‘2023_Hyb_HIPS_LNK_MV_NP_Final_KL_Curated.xlsx’. This information for the hybrid 2023 Ames and Crawfordsville locations was taken from the file ‘2_KRN_trait_HYBRID_2023_compiled.xlsx’. This information for the inbred 2023 Lincoln and Missouri Valley locations was taken from the file ‘2023_Inbred_Hips_Ear_Phenotyping_MV_LNK_Final_KL_Curated.xlsx’.
- **Data Processing Notes:** Plots 367 and 866 at North Platte was calculated using 3 ears rather than 4 as the remaining observation was an order of magnitude larger than all others. In the 2022 inbreds, plot 677 at Scottsbluff was calculated as the mean of 5 ears rather than 6 as the remaining observation appeared to be due to a data entry error. In the 2022 inbreds, values in Crawfordsville less than 8, values in Lincoln less than 5, values in Missouri Valley less than 6 and values in Scottsbluff greater than 18 were visual outliers and converted to missing values. In the 2023 hybrids, all values less than 11.25 were visual outliers and converted to missing values.

kernelsPerEar

- **Definition:** This is the mean number of kernels per ear from the plot.
- **Units:** NA
- **Raw Data Sources:** This information for hybrids at the 2022 Scottsbluff, North Platte, Lincoln, and Missouri Valley locations was taken from the file ‘plotleveleardata_v2.csv’. This information for the hybrid 2022 Ames and Crawfordsville locations was taken from the files in the folder ‘6 Seed Traits Station’. This information for the 2022 inbreds at the Lincoln and Missouri Valley locations was taken from the file ‘2022 Inbred HIPS Ear Data - Turkus Curated 231117.csv’ and converted from millimeters. This information for the inbred 2022 Ames and Crawfordsville locations was taken from the file ‘6_seed_trait_INBRED_2022_compiled_v2.xlsx’. This information for the hybrids at the 2023 North Platte, Lincoln, and Missouri Valley locations was taken from the file ‘2023_Hyb_HIPS_LNK_MV_NP_Final_KL_Curated.xlsx’. This information for the hybrid 2023 Ames and Crawfordsville locations was taken from the file ‘6_seed_trait_HYBRID_2023_compiled.xlsx’. This information for the inbred 2023 Lincoln and Missouri Valley locations was taken from the file ‘2023_Inbred_Hips_Ear_Phenotyping_MV_LNK_Final_KL_Curated.xlsx’.
- **Data Processing Notes:** It does not account for kernels that fell off the cob between harvest and phenotyping. Plot 1381 at North Platte was calculated using 3 ears rather than 4 as the remaining observation was an order of magnitude larger than the other observations. In the 2022 inbreds, Scottsbluff plot 162 was a visual outlier and converted to a missing value. Furthermore, values in Ames greater than 630, values in Crawfordsville greater than 625, values in Lincoln greater than 500, values in Missouri Valley

greater than 525, and values in Scottsbluff greater than 600 were visual outliers and were converted to missing values. In the 2023 hybrids, it appeared that all three ears from plot 23-C-1746688 had been counted together, so the total number was divided by 3. Furthermore, all values less than 250 and values in North Platte greater than 750 were visual outliers and converted to missing values. In the 2023 inbreds, values in Lincoln greater than 450 and values in Missouri Valley greater than 600 were visual outliers and converted to missing values.

hundredKernelMass

- **Definition:** This is the mean weight of one hundred kernels from one ear from the plot in grams after to shelling (i.e. with the kernels detached from the cob).
- **Units:** g
- **Raw Data Sources:** This information for hybrids at the 2022 Scottsbluff, North Platte, Lincoln, and Missouri Valley locations was taken from the file 'plotleveleardata_v2.csv'. This information was not directly collected for Ames or Crawfordsville locations. This information for the 2022 inbreds at the Lincoln and Missouri Valley locations was taken from the file '2022 Inbred HIPS Ear Data - Turkus Curated 231117.csv'. This information for the hybrids at the 2023 North Platte, Lincoln, and Missouri Valley locations was taken from the file '2023_Hyb_HIPS_LNK_MV_NP_Final_KL_Curated.xlsx'. This information for the inbred 2023 Lincoln and Missouri Valley locations was taken from the file '2023_Inbred_Hips_Ear_Phenotyping_MV_LNK_Final_KL_Curated.xlsx'.
- **Data Processing Notes:** This data was only collected for the Scottsbluff, North Platte, Lincoln, and Missouri Valley locations if there were at least 100 kernels on the ear. For the Ames and Crawfordsville locations, this was calculated as `kernelMassPerEar` divided by `kernelsPerEar` multiplied by one hundred. Plots 5122 and 5245 at Lincoln, and 835, 838, 1169, and 1211 at North Platte were calculated using 3 ears rather than 4, as the remaining observation was an order of magnitude larger than the others. In the 2022 inbreds, values greater than 50 grams, values in Crawfordsville less than 10 or greater than 37.5 grams, values in Lincoln greater than 31 grams, and values in Scottsbluff greater than 32 grams were visual outliers and were converted to missing values. In the 2023 hybrids, plots 23-C-1746410, 23-C-1746525, and 23-C-1746805 were calculated as the mean of 2 ears, and plot 23-C-1746706 was calculated as the mean of 1 ear, rather than 3 ears, as the remaining observation(s) appeared to be data entry errors. Furthermore, all values less than 15 or greater than 45 grams were visual outliers and converted to missing values. In the 2023 inbreds, values in Lincoln greater than 35 grams were visual outliers and converted to missing values.

kernelMassPerEar

- **Definition:** This is the mean mass of kernels in grams, per ear.
- **Units:** g
- **Raw Data Sources:** This trait was not directly measured in the Scottsbluff, North Platte, Lincoln, and Missouri Valley locations. This information for the hybrid 2022 Ames and Crawfordsville locations was taken from the files in the folder '6 Seed Traits Station'. This information for the inbred 2022 Ames and Crawfordsville locations was taken from the file '6_seed_trait_INBRED_2022_compiled_v2.xlsx'. This information for the hybrid 2023 Ames and Crawfordsville locations was taken from the file '6_seed_trait_HYBRID_2023_compiled.xlsx'.
- **Data Processing Notes:** For the North Platte, Scottsbluff, Lincoln, and Missouri Valley locations, this is calculated as the difference between the ear weight and `shelledCobMass` . In the cases that significant spillage was denoted for plots from the Ames and Crawfordsville locations, the same estimation used at Lincoln was used in place of the direct measurement. Plot 256 at Missouri Valley and plot 664 at North Platte were calculated using 3 ears rather than 4 as the remaining observation was an order of magnitude

larger than the other observations. In Ames and Crawfordsville, some ears' kernels were re-weighed due to an off-balance scale. In this case, the re-weighing values replaced the original values. In the 2022 inbreds, values in Ames greater than 155 grams, values in Crawfordsville greater than 150 grams, values in Lincoln greater than 100 grams, values in Missouri Valley greater than 135 grams, and values n Scottsbluff greater than 148 were visual outlers and were converted to missing values. In the 2023 hybrids, values in Crawfordsville greater than 275 grams were visual outliers and were converted to missing values. In the 2023 inbreds, values in Missouri Valley greater than 150 grams and values in Lincoln greater than 100 grams were visual outliers and were converted to missing values.

shelledCobMass

- **Definition:** This is the mean weight of the cob in grams of one ear from the plot after to shelling (i.e. with the kernels detached from the cob).
- **Units:** g
- **Raw Data Sources:** This information for hybrids at the 2022 Scottsbluff, North Platte, Lincoln, and Missouri Valley locations was taken from the file 'plotleveleardata_v2.csv'. This information for the hybrid 2022 Ames and Crawfordsville locations was taken from the files in the folder '5 Cob Traits Station'. This information for the 2022 inbreds at the Lincoln and Missouri Valley locations was taken from the file '2022 Inbred HIPS Ear Data - Turkus Curated 231117.csv'. This information for the inbred 2022 Ames and Crawfordsville locations was taken from the file '5_cob_trait_INBRED_2022_compiled_v2.xlsx'. This information for the hybrids at the 2023 North Platte, Lincoln, and Missouri Valley locations was taken from the file '2023_Hyb_HIPS_LNK_MV_NP_Final_KL_Curated.xlsx'. This information for the hybrid 2023 Ames and Crawfordsville locations was taken from the file '5_cob_Traits_HYBRID_2023_compiled.xlsx'. This information for the inbred 2023 Lincoln and Missouri Valley locations was taken from the file '2023_Inbred_Hips_Ear_Phenotyping_MV_LNK_Final_KL_Curated.xlsx'.
- **Data Processing Notes:** Plot 1226 at North Platte 2022 was calculated using 3 rather than 4 ears as the remaining observation was an order of magnitude larger than all other observations. In the 2022 inbreds, all values greater than 40 grams, values in Crawfordsville greater than 35 grams, values in Lincoln greater than 25 grams, and values in Missouri Valley greater than 31 grams were visual outliers and were converted to missing values. In the 2023 hybrids, values in Ames greater than 45 grams, values in Crawfordsville greater than 40 grams, values in Lincoln greater than 35 grams, and values in Missouri Valley and North Platte less than 10 or greater than 42.5 grams were visual outliers and were converted to missing values. In the 2023 inbreds, plots 2271, and 2039 in Lincoln and plots 367(4 ears) and 681 in Missouri Valley were calculated as means of 5 (rather than 6) ears, as the remaining observation appeared to be data entry errors. Furthermore, values in Lincoln greater than 30 grams and values in Missouri Valley greater than 35 grams were visual outliers and converted to missing values.

percentMoisture

- **Definition:** This is the percent moisture measured by NIR.
- **Units:** percent
- **Raw Data Sources:** For the hybrid 2022 Lincoln, Missouri Valley, North Platte, and Scottsbluff locations, this information was taken from the file 'HybridHIPS_plotlevelNIR_v2.5.csv'.
- **Data Processing Notes:** In Missouri Valley 2022 hybrids, five replicates per plot were used, and three replicates per plot were used in all other locations. In the case any NIR measurement for a replicate was less than zero, this entire replicate was dropped prior to computing the median.

percentStarch

- **Definition:** This is the median proportion of the grain composed of starch as a percent of dry matter as measured by NIR spectrometry.
- **Units:** percent
- **Raw Data Sources:** For the hybrid 2022 Lincoln, Missouri Valley, North Platte, and Scottsbluff locations, this information was taken from the file ‘HybridHIPS_plotlevelNIR_v2.5.csv’.
- **Data Processing Notes:** In Missouri Valley 2022 hybrids, five replicates per plot were used, and three replicates per plot were used in all other locations. In the case any NIR measurement for a replicate was less than zero, this entire replicate was dropped prior to computing the median.

percentProtein

- **Definition:** This is the median proportion of the grain composed of protein as a percent of dry matter as measured by NIR spectrometry.
- **Units:** percent
- **Raw Data Sources:** For the hybrid 2022 Lincoln, Missouri Valley, North Platte, and Scottsbluff locations, this information was taken from the file ‘HybridHIPS_plotlevelNIR_v2.5.csv’.
- **Data Processing Notes:** In Missouri Valley 2022 hybrids, five replicates per plot were used, and three replicates per plot were used in all other locations. In the case any NIR measurement for a replicate was less than zero, this entire replicate was dropped prior to computing the median.

percentOil

- **Definition:** This is the median proportion of the grain composed of oil as a percent of dry matter as measured by NIR spectrometry.
- **Units:** percent
- **Raw Data Sources:** For the hybrid 2022 Lincoln, Missouri Valley, North Platte, and Scottsbluff locations, this information was taken from the file ‘HybridHIPS_plotlevelNIR_v2.5.csv’.
- **Data Processing Notes:** In Missouri Valley 2022 hybrids, five replicates per plot were used, and three replicates per plot were used in all other locations. In the case any NIR measurement for a replicate was less than zero, this entire replicate was dropped prior to computing the median.

percentFiber

- **Definition:** This is the median proportion of the grain composed of fiber as a percent of dry matter as measured by NIR spectrometry.
- **Units:** percent
- **Raw Data Sources:** For the hybrid 2022 Lincoln, Missouri Valley, North Platte, and Scottsbluff locations, this information was taken from the file ‘HybridHIPS_plotlevelNIR_v2.5.csv’.
- **Data Processing Notes:** In Missouri Valley 2022 hybrids, five replicates per plot were used, and three replicates per plot were used in all other locations. In the case any NIR measurement for a replicate was less than zero, this entire replicate was dropped prior to computing the median.

percentAsh

- **Definition:** This is the median proportion of the grain composed of ash as a percent of dry matter as measured by NIR spectrometry.
- **Units:** percent

- **Raw Data Sources:** For the hybrid 2022 Lincoln, Missouri Valley, North Platte, and Scottsbluff locations, this information was taken from the file 'HybridHIPS_plotlevelNIR_v2.5.csv'.
- **Data Processing Notes:** In Missouri Valley 2022 hybrids, five replicates per plot were used, and three replicates per plot were used in all other locations. In the case any NIR measurement for a replicate was less than zero, this entire replicate was dropped prior to computing the median.

kernelColor

- **Definition:** This is the color of the kernels from the plot.
- **Units:** NA
- **Raw Data Sources:** This data was not collected for the Ames and Crawfordsville locations. This information for 2022 hybrids was taken from the file 'plotleveleardata_v2.csv'. This information for 2022 inbreds was taken from the file 2022 Inbred HIPS Ear Data - Turkus Curated 231117.csv'. This information for 2023 hybrids was taken from the file '2023_Hyb_HIPS_LNK_MV_NP_Final_KL_Curated.xlsx'. This information for 2023 inbreds was taken from the file '2023_Inbred_Hips_Ear_Phenotyping_MV_LNK_Final_KL_Curated.xlsx'.
- **Data Processing Notes:** In 2022, these values were manually entered prior to shelling and curated during data processing to select options. In 2023, these values were taken from kernels after shelling by scanning the QR code associated with the closest pre-defined color.

percentLodging

- **Definition:** This is the percent of plants that lodged in the middle two rows of the plot for hybrids and in the whole plot for inbreds.
- **Units:** percent
- **Raw Data Sources:** This data was not collected at the Scottsbluff and Lincoln locations. This information for the 2022 Missouri Valley, Crawfordsville, and Ames locations was taken from the sheets 'RawData (4-Row)' and 'RawData (2-Row)' for hybrids and inbreds, respectively, in the file 'YTMC_Lisa_Plot_Coordinates_v4.xlsx'. This information for North Platte in 2022 was taken from the sheets 'No Irr Data', 'Reduced Irr Data', and 'Full Data' in the file '2022_Schnable_HIPS_data_v4.xlsx'. This information for North Platte 2023 was taken from the file '2023 Schnable hips_data_v2.xlsx'.
- **Data Processing Notes:** In cases where there was both stalk lodging and root lodging recorded, this is the sum of those fields divided by `totalStandCount` and multiplied by 100.

harvestDate

- **Definition:** This is the date and time the plot was harvested by the combine for hybrids, and the date of hand harvest for inbreds.
- **Units:** NA
- **Raw Data Sources:** This information for the hybrid 2022 Lincoln location was taken from the sheet 'Combined Dataset' in the file 'Summary of Lincoln Hybrid HIPS 2022 Data.xlsx'. For the hybrid 2022 Scottsbluff location, this information was taken from the file 'Dipak Corn22_HM.xlsx'. For the hybrid 2022 Missouri Valley, Ames, and Crawfordsville locations, this data was taken from the file 'YTMC_Lisa_Plot_Coordinates_v4.xlsx'. This information for the North Platte 2022 location was taken from the sheets 'No Irr Data', 'Reduced Irr Data', and 'Full Data' in the file '2022_Schnable_HIPS_data_v4.xlsx'. This information for the 2023 North Platte location was taken from the file '2023 Schnable hips_data_v2.xlsx'. This information for the 2023 Lincoln location was taken from the file '231127 Hybrid HIPS Lincoln - Combine Harvest Data - Turkus Curated.csv'. This information for the 2023 Missouri Valley, Ames, and Crawfordsville locations was taken from the sheet '4-row plots' in the file

'2023_yield_ICIA_v3.xlsx'. For inbreds in 2022, this information was taken from the file 'HIPS_Sites_Summary_2022.xlsx'. In 2023 inbreds, this was taken from the file 'HIPS_Sites_Summary_2023.xlsx'.

- **Data Processing Notes:** In the case where a single date was not available for hand harvest of inbreds, the first date in the range was used.

notes

- **Definition:** This contains any notes regarding the the plot.
- **Units:** NA
- **Raw Data Sources:** Any raw data files used as described for other fields that contained notes column(s).
- **Data Processing Notes:** This information was aggregated across all notes columns for a plot in the original data files.

Location Notes

Lincoln 2022

The alley length in both the Inbred HIPS and Hybrid HIPS fields was 2.5 feet, and the distance between seeds in a row was 6 inches. Nitrogen application was made on March 29, 2022 at the treatment-specified rates (Low: 75 lbs/acre, Medium: 150 lbs/acre, High: 225 lbs/acre) with urea ammonium nitrate (32-0-0) Conventional tillage was done prior to planting.

Inbred HIPS field

Plots in the Inbred HIPS field were 10 feet long center to center, including 7.5 feet of plants and 2.5 feet of alley. The planting date was May 5, 2022. Plots were hand-harvested (4 ears per plot) on October 8, 2022. The previous crop in the field was soybeans. The GPS coordinates for the field corners were:

- NE corner: 40°51'32.96"N, 96°35'50.40"W
- SW corner: 40°51'31.39"N, 96°35'54.10"W
- NW Corner: 40°51'32.93"N, 96°35'54.10"W
- SE Corner: 40°51'31.44"N, 96°35'50.36"W

Plots were two-row plots, and the field contained 16 plots (160 feet) north to south and 58 plots (290 feet) east to west.

- 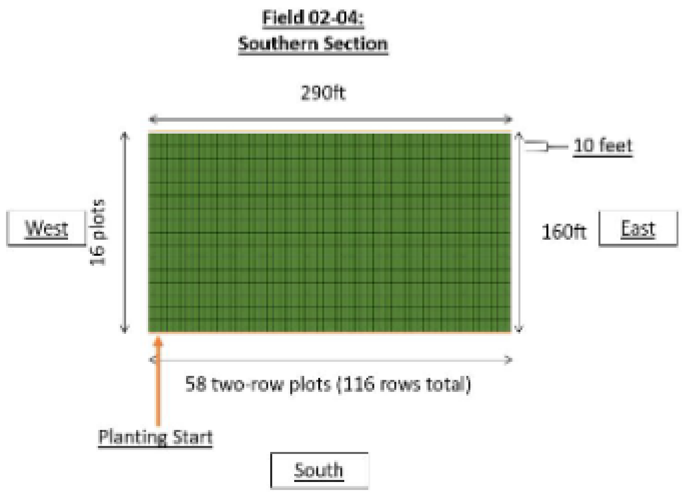

Hybrid HIPS field

Plots in the Hybrid HIPS field were 20 feet long center to center, including 17.5 feet of plants and 2.5 feet of alley. The planting date was May 22, 2022. Plots were hand-harvested (4 ears per plot) on October 1, 2022 and combine harvested on October 10, 2022. The previous crop in the field was maize. The GPS coordinates for the field corners were:

- NE corner: 40°51’8.59”N, 96°36’50.26”W
- SW corner: 40°51’7.25”N, 96°37’0.54”W
- NW Corner: 40°51’8.70”N, 96°37’0.52”W
- SE Corner: 40°51’7.12”N, 96°36’50.29”W

The field was 150 feet north to south and 800 feet east to west. Differential weed pressure existed throughout the field according to James.

- 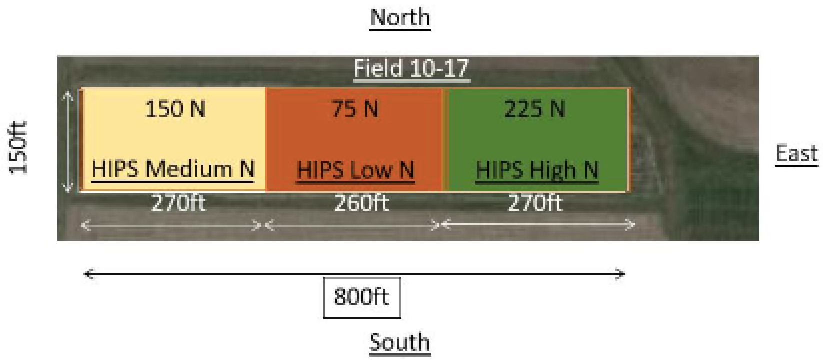

Missouri Valley 2022

175 lbs of urea was applied on both the hybrid and inbred fields in this location. The nitrogen fertilizer was applied on June 6, 2022. The field latitude is 41.671747 N and the longitude is -95.943982 W. The fields were not irrigated. Planting was completed on April 29, 2022 and the harvest was completed on October 11, 2022. The previous crop for was corn.

Inbred HIPS field

There were 752 plots (376 plots per replication) in this field. The plots were two-row plots. The plot numbers shown in the image correspond to those used in the QR codes.

		Row									
		53	54	55	56	57	58	59	60	61	
Range	86	Fill	Fill	376	375	374	373	372	371	370	
	85	361	362	363	364	365	366	367	368	369	
	84	360	359	358	357	356	355	354	353	352	
	83	343	344	345	346	347	348	349	350	351	
	82	342	341	340	339	338	337	336	335	334	
	81	325	326	327	328	329	330	331	332	333	
	80	324	323	322	321	320	319	318	317	316	
	79	307	308	309	310	311	312	313	314	315	
	78	306	305	304	303	302	301	300	299	298	
	77	289	290	291	292	293	294	295	296	297	
	76	288	287	286	285	284	283	282	281	280	
	75	271	272	273	274	275	276	277	278	279	
	74	270	269	268	267	266	265	264	263	262	
	73	253	254	255	256	257	258	259	260	261	
	72	252	251	250	249	248	247	246	245	244	
	71	235	236	237	238	239	240	241	242	243	
	70	234	233	232	231	230	229	228	227	226	
	69	217	218	219	220	221	222	223	224	225	
	68	216	215	214	213	212	211	210	209	208	
	67	199	200	201	202	203	204	205	206	207	
	66	198	197	196	195	194	193	192	191	190	Rep 2
	65	181	182	183	184	185	186	187	188	189	
	64	180	179	178	177	176	175	174	173	172	
	63	163	164	165	166	167	168	169	170	171	
	62	162	161	160	159	158	157	156	155	154	
	61	145	146	147	148	149	150	151	152	153	
	60	144	143	142	141	140	139	138	137	136	
	59	127	128	129	130	131	132	133	134	135	
	58	126	125	124	123	122	121	120	119	118	
	57	109	110	111	112	113	114	115	116	117	
	56	108	107	106	105	104	103	102	101	100	
	55	91	92	93	94	95	96	97	98	99	
	54	90	89	88	87	86	85	84	83	82	
	53	73	74	75	76	77	78	79	80	81	
	52	72	71	70	69	68	67	66	65	64	
	51	55	56	57	58	59	60	61	62	63	
	50	54	53	52	51	50	49	48	47	46	
	49	37	38	39	40	41	42	43	44	45	
	48	36	35	34	33	32	31	30	29	28	
	47	19	20	21	22	23	24	25	26	27	
	46	18	17	16	15	14	13	12	11	10	
	45	1	2	3	4	5	6	7	8	9	
	44	Fill	Fill	376	375	374	373	372	371	370	
	43	361	362	363	364	365	366	367	368	369	
	42	360	359	358	357	356	355	354	353	352	
	41	343	344	345	346	347	348	349	350	351	
	40	342	341	340	339	338	337	336	335	334	
	39	325	326	327	328	329	330	331	332	333	
	38	324	323	322	321	320	319	318	317	316	
	37	307	308	309	310	311	312	313	314	315	
	36	306	305	304	303	302	301	300	299	298	
	35	289	290	291	292	293	294	295	296	297	
	34	288	287	286	285	284	283	282	281	280	
	33	271	272	273	274	275	276	277	278	279	
	32	270	269	268	267	266	265	264	263	262	
	31	253	254	255	256	257	258	259	260	261	
	30	252	251	250	249	248	247	246	245	244	
	29	235	236	237	238	239	240	241	242	243	
	28	234	233	232	231	230	229	228	227	226	
	27	217	218	219	220	221	222	223	224	225	
	26	216	215	214	213	212	211	210	209	208	
	25	199	200	201	202	203	204	205	206	207	
	24	198	197	196	195	194	193	192	191	190	Rep 1
	23	181	182	183	184	185	186	187	188	189	
	22	180	179	178	177	176	175	174	173	172	
	21	163	164	165	166	167	168	169	170	171	
	20	162	161	160	159	158	157	156	155	154	
	19	145	146	147	148	149	150	151	152	153	
	18	144	143	142	141	140	139	138	137	136	
	17	127	128	129	130	131	132	133	134	135	
	16	126	125	124	123	122	121	120	119	118	
	15	109	110	111	112	113	114	115	116	117	
	14	108	107	106	105	104	103	102	101	100	
	13	91	92	93	94	95	96	97	98	99	
	12	90	89	88	87	86	85	84	83	82	

11	73	74	75	76	77	78	79	80	81
10	72	71	70	69	68	67	66	65	64
9	55	56	57	58	59	60	61	62	63
8	54	53	52	51	50	49	48	47	46
7	37	38	39	40	41	42	43	44	45
6	36	35	34	33	32	31	30	29	28
5	19	20	21	22	23	24	25	26	27
4	18	17	16	15	14	13	12	11	10
3	1	2	3	4	5	6	7	8	9
	53	54	55	56	57	58	59	60	61

Hybrid HIPS field

There were 176 plots (88 plots per replication) in this field. The plots were four-row plots.

	1	2	3	4	
45	85	86	87	88	
44	81	82	83	84	
43	77	78	79	80	
42	73	74	75	76	
41	69	70	71	72	
40	65	66	67	68	
39	61	62	63	64	
38	57	58	59	60	
37	53	54	55	56	
36	49	50	51	52	
35	45	46	47	48	Rep 2
34	41	42	43	44	
33	37	38	39	40	
32	33	34	35	36	
31	29	30	31	32	
30	25	26	27	28	
29	21	22	23	24	
28	17	18	19	20	
27	13	14	15	16	
26	9	10	11	12	
25	5	6	7	8	
24	1	2	3	4	
23	85	86	87	88	
22	81	82	83	84	
21	77	78	79	80	
20	73	74	75	76	
19	69	70	71	72	
18	65	66	67	68	
17	61	62	63	64	
16	57	58	59	60	
15	53	54	55	56	
14	49	50	51	52	
13	45	46	47	48	Rep 1
12	41	42	43	44	
11	37	38	39	40	
10	33	34	35	36	
9	29	30	31	32	
8	25	26	27	28	
7	21	22	23	24	
6	17	18	19	20	
5	13	14	15	16	
4	9	10	11	12	
3	5	6	7	8	
2	1	2	3	4	

The plot numbers shown in the image correspond to those used in the QR codes. The QR codes mis-assigned the replicate numbers. These have been fixed in the dataset.

North Platte 2022

Only the Hybrid HIPS population was grown at this location. Plot numbers are unique across the North Platte location, but range and row numbers were duplicated in each field. Each field is a different irrigation treatment. To account for this, the location was split in 3 by irrigation treatment in the data. North Platte1 is the full irrigation field; North Platte2 is the partial irrigation field, and North Platte3 is the dryland, i.e. rainfed, field. The plots were 4-row, 20 foot plots, with 17 feet of the plot planted and 3 feet of alley. Nitrogen treatments were blocked within each irrigation treatment. The full irrigation field was planted on May 17, 2022, and harvested on October 21, 2022 and November 1, 2022 with planted plot lengths of 17.5 feet. The partial irrigation field was planted on May 17, 2022 and harvested October 26 - 28, 2022 with planted plot lengths of 17.5 feet. The dryland field was planted on May 18, 2022 and harvested on October 19-21 and 24, 2022 with planted plot lengths of 17 feet. The previous crop was soybeans. Nitrogen was applied on June 16, 2022 as 32-0-0 with a 360 Y drop applicator.

Full Irrigation

		1	2	3	4	5	6	7	8	9	10	11	12	13	14	15	16	Nitrogen Treatment	
**Range**	**35**	513	514	515	516	517	518	519	520	521	522	523	524	525	526	527	528	High	
	**34**	497	498	499	500	501	502	503	504	505	506	507	508	509	510	511	512	High	
	**33**	481	482	483	484	485	486	487	488	489	490	491	492	493	494	495	496	High	Onion sa
	**32**	465	466	467	468	469	470	471	472	473	474	475	476	477	478	479	480	High	
	**31**	449	450	451	452	453	454	455	456	457	458	459	460	461	462	463	464	High	
	**30**	433	434	435	436	437	438	439	440	441	442	443	444	445	446	447	448	High	
	**29**	417	418	419	420	421	422	423	424	425	426	427	428	429	430	431	432	High	
	**28**	401	402	403	404	405	406	407	408	409	410	411	412	413	414	415	416	High	
	**27**	385	386	387	388	389	390	391	392	393	394	395	396	397	398	399	400	High	
	**26**	369	370	371	372	373	374	375	376	377	378	379	380	381	382	383	384	High	
	**25**	353	354	355	356	357	358	359	360	361	362	363	364	365	366	367	368	High	
	**24**	Border	Border	Border	Border	Border	Border	Border	Border	Border	Border	Border	Border	Border	Border	Border	Border	Border	
	**23**	337	338	339	340	341	342	343	344	345	346	347	348	349	350	351	352	Medium	
	**22**	321	322	323	324	325	326	327	328	329	330	331	332	333	334	335	336	Medium	Onion sa
	**21**	305	306	307	308	309	310	311	312	313	314	315	316	317	318	319	320	Medium	
	**20**	289	290	291	292	293	294	295	296	297	298	299	300	301	302	303	304	Medium	
	**19**	273	274	275	276	277	278	279	280	281	282	283	284	285	286	287	288	Medium	
	**18**	257	258	259	260	261	262	263	264	265	266	267	268	269	270	271	272	Medium	
	**17**	241	242	243	244	245	246	247	248	249	250	251	252	253	254	255	256	Medium	
	**16**	225	226	227	228	229	230	231	232	233	234	235	236	237	238	239	240	Medium	
	**15**	209	210	211	212	213	214	215	216	217	218	219	220	221	222	223	224	Medium	
	**14**	193	194	195	196	197	198	199	200	201	202	203	204	205	206	207	208	Medium	
	**13**	177	178	179	180	181	182	183	184	185	186	187	188	189	190	191	192	Medium	
	**12**	Border	Border	35	Border	Border	Border	Border	Border	Border	Border	Border	Border	Border	Border	Border	Border	Border	
	**11**	161	162	Border	164	165	166	167	168	169	170	171	172	173	174	175	176	Low	Onion sa
	**10**	145	146	163	148	149	150	151	152	153	154	155	156	157	158	159	160	Low	
	**9**	129	130	147	132	133	134	135	136	137	138	139	140	141	142	143	144	Low	
	**8**	113	114	131	116	117	118	119	120	121	122	123	124	125	126	127	128	Low	
	**7**	97	98	115	100	101	102	103	104	105	106	107	108	109	110	111	112	Low	
	**6**	81	82	99	84	85	86	87	88	89	90	91	92	93	94	95	96	Low	
	**5**	65	66	83	68	69	70	71	72	73	74	75	76	77	78	79	80	Low	
	**4**	49	50	67	52	53	54	55	56	57	58	59	60	61	62	63	64	Low	
	**3**	33	34	51	36	37	38	39	40	41	42	43	44	45	46	47	48	Low	
	**2**	17	18	19	20	21	22	23	24	25	26	27	28	29	30	31	32	Low	
	**1**	1	2	3	4	5	6	7	8	9	10	11	12	13	14	15	16	Low	Onion sa
		**1**	**2**	**3**	**4**	**5**	**6**	**7**	**8**	**9**	**10**	**11**	**12**	**13**	**14**	**15**	**16**		
		**Row**																	

Colour	Meaning
Red	Plots lost, border seed
Yellow	Seed mixed (tube jammed)
Blue	Order
Green	Possibly gone, check
Purple	Two borders, check for missing envelope

Partial Irrigation

		1	2	3	4	5	6	7	8	9	10	11	12	13	14	15	16	Nitrogen Treatment	
**Range**	**35**	1041	1042	1043	1044	1045	1046	1047	1048	1049	1050	1051	1052	1053	1054	1055	1056	Low	
	**34**	1025	1026	1027	1028	1029	1030	1031	1032	1033	1034	1035	1036	1037	1038	1039	1040	Low	
6x bags of 3	**33**	1009	1010	1011	1012	1013	1014	1015	1016	1017	1018	1019	1020	1021	1022	1023	1024	Low	Onion sacl
	**32**	993	994	995	996	997	998	999	1000	1001	1002	1003	1004	1005	1006	1007	1008	Low	
	**31**	977	978	979	980	981	982	983	984	985	986	987	988	989	990	991	992	Low	
	**30**	961	962	963	964	965	966	967	968	969	970	971	972	973	974	975	976	Low	
	**29**	945	946	947	948	949	950	951	952	953	954	955	956	957	958	959	960	Low	
	**28**	929	930	931	932	933	934	935	936	937	938	939	940	941	942	943	944	Low	
	**27**	913	914	915	916	917	918	919	920	921	922	923	924	925	926	927	928	Low	
	**26**	897	898	899	900	901	902	903	904	905	906	907	908	909	910	911	912	Low	
	**25**	881	882	883	884	885	886	887	888	889	890	891	892	893	894	895	896	Low	
	**24**	Border	Border	Border	Border	Border	Border	Border	Border	Border	Border	Border	Border	Border	Border	Border	Border	Border	
	**23**	865	866	867	868	869	870	871	872	873	874	875	876	877	878	879	880	Medium	
bags of 10	**22**	849	850	851	852	853	854	855	856	857	858	859	860	861	862	863	864	Medium	Onion sacl
	**21**	833	834	835	836	837	838	839	840	841	842	843	844	845	846	847	848	Medium	
	**20**	817	818	819	820	821	822	823	824	825	826	827	828	829	830	831	832	Medium	
	**19**	801	802	803	804	805	806	807	808	809	810	811	812	813	814	815	816	Medium	
	**18**	785	786	787	788	789	790	791	792	793	794	795	796	797	798	799	800	Medium	
	**17**	769	770	771	772	773	774	775	776	777	778	779	780	781	782	783	784	Medium	
	**16**	753	754	755	756	757	758	759	760	761	762	763	764	765	766	767	768	Medium	
	**15**	737	738	739	740	741	742	743	744	745	746	747	748	749	750	751	752	Medium	
	**14**	721	722	723	724	725	726	727	728	729	730	731	732	733	734	735	736	Medium	
	**13**	705	706	707	708	709	710	711	712	713	714	715	716	717	718	719	720	Medium	
	**12**	Border	Border	Border	Border	Border	Border	Border	Border	Border	Border	Border	Border	Border	Border	Border	Border	Border	
bags of 10	**11**	689	690	691	692	693	694	695	696	697	698	699	700	701	702	703	704	High	Onion sacl
	**10**	673	674	675	676	677	678	679	680	681	682	683	684	685	686	687	688	High	
	**9**	657	658	659	660	661	662	663	664	665	666	667	668	669	670	671	672	High	
	**8**	641	642	643	644	645	646	647	648	649	650	651	652	653	654	655	656	High	
	**7**	625	626	627	628	629	630	631	632	633	634	635	636	637	638	639	640	High	
	**6**	609	610	611	612	613	614	615	616	617	618	619	620	621	622	623	624	High	
	**5**	593	594	595	596	597	598	599	600	601	602	603	604	605	606	607	608	High	
	**4**	577	578	579	580	581	582	583	584	585	586	587	588	589	590	591	592	High	
	**3**	561	562	563	564	565	566	567	568	569	570	571	572	573	574	575	576	High	
	**2**	545	546	547	548	549	550	551	552	553	554	555	556	557	558	559	560	High	
bags of 10	**1**	529	530	531	532	533	534	535	536	537	538	539	540	541	542	543	544	High	Onion sacl
		**1**	**2**	**3**	**4**	**5**	**6**	**7**	**8**	**9**	**10**	**11**	**12**	**13**	**14**	**15**	**16**		

No Irrigation																									
		1	2	3	4	5	6	7	8	9	10	11	12	13	14	15	16	17	18	19	20	21	22	Nitrogen Treatment	
Range	29	1577	1578	1579	1580	1581	1582	1583	1584	1585	1586	1587	1588	Border	Border	1589	1590	1591	1592	1593	1594	1595	1596	Medium	
	28	1557	1558	1559	1560	1561	1562	1563	1564	1565	1566	1567	1568	Border	Border	1569	1570	1571	1572	1573	1574	1575	1576	Medium	
of 3	27	1537	1538	1539	1540	1541	1542	1543	1544	1545	1546	1547	1548	Border	Border	1549	1550	1551	1552	1553	1554	1555	1556	Medium	
	26	1517	1518	1519	1520	1521	1522	1523	1524	1525	1526	1527	1528	Border	Border	1529	1530	1531	1532	1533	1534	1535	1536	Medium	
	25	1497	1498	1499	1500	1501	1502	1503	1504	1505	1506	1507	1508	Border	Border	1509	1510	1511	1512	1513	1514	1515	1516	Medium	
	24	1477	1478	1479	1480	1481	1482	1483	1484	1485	1486	1487	1488	Border	Border	1489	1490	1491	1492	1493	1494	1495	1496	Medium	
	23	1457	1458	1459	1460	1461	1462	1463	1464	1465	1466	1467	1468	Border	Border	1469	1470	1471	1472	1473	1474	1475	1476	Medium	2x onion
	22	1437	1438	1439	1440	1441	1442	1443	1444	1445	1446	1447	1448	Border	Border	1449	1450	1451	1452	1453	1454	1455	1456	Medium	
	21	1417	1418	1419	1420	1421	1422	1423	1424	1425	1426	1427	1428	Border	Border	1429	1430	1431	1432	1433	1434	1435	1436	Medium	
	20	Border	Border	Border	Border	Border	Border	Border	Border	Border	Border	Border	Border	Border	Border	Border	Border	Border	Border	Border	Border	Border	Border	Border	
	19	1397	1398	1399	1400	1401	1402	1403	1404	1405	1406	1407	1408	Border	Border	1409	1410	1411	1412	1413	1414	1415	1416	High	
	18	1377	1378	1379	1380	1381	1382	1383	1384	1385	1386	1387	1388	Border	Border	1389	1390	1391	1392	1393	1394	1395	1396	High	
	17	1357	1358	1359	1360	1361	1362	1363	1364	1365	1366	1367	1368	Border	Border	1369	1370	1371	1372	1373	1374	1375	1376	High	
0	16	1337	1338	1339	1340	1341	1342	1343	1344	1345	1346	1347	1348	Border	Border	1349	1350	1351	1352	1353	1354	1355	1356	High	
	15	1317	1318	1319	1320	1321	1322	1323	1324	1325	1326	1327	1328	Border	Border	1329	1330	1331	1332	1333	1334	1335	1336	High	
	14	1297	1298	1299	1300	1301	1302	1303	1304	1305	1306	1307	1308	Border	Border	1309	1310	1311	1312	1313	1314	1315	1316	High	
	13	1277	1278	1279	1280	1281	1282	1283	1284	1285	1286	1287	1288	Border	Border	1289	1290	1291	1292	1293	1294	1295	1296	High	
	12	1257	1258	1259	1260	1261	1262	1263	1264	1265	1266	1267	1268	Border	Border	1269	1270	1271	1272	1273	1274	1275	1276	High	2x onion
	11	1237	1238	1239	1240	1241	1242	1243	1244	1245	1246	1247	1248	Border	Border	1249	1250	1251	1252	1253	1254	1255	1256	High	
	10	Border	Border	Border	Border	Border	Border	Border	Border	Border	Border	Border	Border	Border	Border	Border	Border	Border	Border	Border	Border	Border	Border	Border	
	9	1217	1218	1219	1220	1221	1222	1223	1224	1225	1226	1227	1228	Border	Border	1229	1230	1231	1232	1233	1234	1235	1236	Low	
	8	1197	1198	1199	1200	1201	1202	1203	1204	1205	1206	1207	1208	Border	Border	1209	1210	1211	1212	1213	1214	1215	1216	Low	
	7	1177	1178	1179	1180	1181	1182	1183	1184	1185	1186	1187	1188	Border	Border	1189	1190	1191	1192	1193	1194	1195	1196	Low	
	6	1157	1158	1159	1160	1161	1162	1163	1164	1165	1166	1167	1168	Border	Border	1169	1170	1171	1172	1173	1174	1175	1176	Low	
0	5	1137	1138	1139	1140	1141	1142	1143	1144	1145	1146	1147	1148	Border	Border	1149	1150	1151	1152	1153	1154	1155	1156	Low	
	4	1117	1118	1119	1120	1121	1122	1123	1124	1125	1126	1127	1128	Border	Border	1129	1130	1131	1132	1133	1134	1135	1136	Low	
	3	1097	1098	1099	1100	1101	1102	1103	1104	1105	1106	1107	1108	Border	Border	1109	1110	1111	1112	1113	1114	1115	1116	Low	
	2	1077	1078	1079	1080	1081	1082	1083	1084	1085	1086	1087	1088	Border	Border	1089	1090	1091	1092	1093	1094	1095	1096	Low	
	1	1057	1058	1059	1060	1061	1062	1063	1064	1065	1066	1067	1068	Border	Border	1069	1070	1071	1072	1073	1074	1075	1076	Low	2x onion
		1	2	3	4	5	6	7	8	9	10	11	12	13	14	15	16	17	18	19	20	21	22		

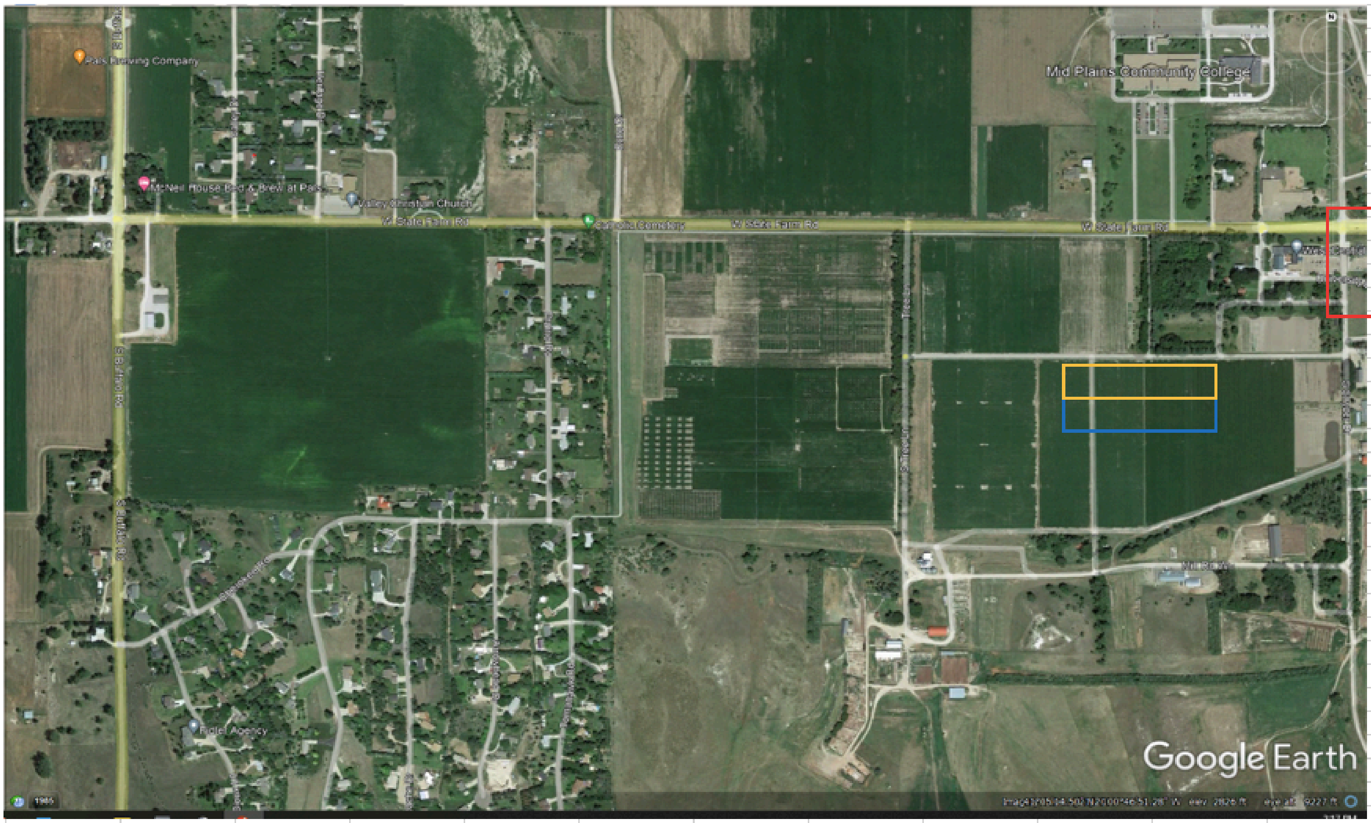

In the image above, the blue rectangle is the approximate location of the full irrigation field, the yellow rectangle is the approximate location of the partial irrigation field, and the red rectangle is the approximate location of the dryland field.

The irrigation amounts and timing for the full and partial fields is as follows:

	Full	Reduced
Date	Inch	Inch
15-Jun-22	0.6	0.3
17-Jun-22	0.6	0.3
22-Jun-22	0.7	0.35
30-Jun-22	0.7	0.35
14-Jul-22	0.6	0.3
20-Jul-22	0.6	0.3
10-Aug-22	0.7	0.35
13-Aug-22	0.7	0.35
16-Aug-22	0.7	0.35
23-Aug-22	0.7	0.35
5-Sep-22	0.7	0.35
8-Sep-22	0.6	0.3
13-Sep-22	0.7	0.35

Scottsbluff 2022

The field was irrigated for one hour from 4:00 p.m. to 5:00 p.m. every Friday evening, for a total of 16.86 inches of irrigation provided over the growing season.. The row spacing was 30”. In-field measurements (flowering time, height data, and combine yield measurements) use the range-row system defined in the sheet ‘Layout (Original)’ of the files ‘Scottsbluff Hybrid HIPS - Summary.xlsx’ and ‘Scottsbluff Inbred HIPS - Summary.xlsx’. In the data, these range-row assignments were dropped in favor of using the range-row assignments listed in the QR codes and used by the ear phenotype and NIR grain composition measurements, which better capture the spatial distance between plots where there are border plots. The geographic location within the field of a plot by its plot number is the same in both layouts, but the range-row numbering is different. The range-row system used in the QR codes and the data is depicted in the sheet ‘Layout (Modified)’ of the files ‘Scottsbluff Hybrid HIPS - Summary’ and ‘Scottsbluff Inbred HIPS - Summary.xlsx’, and shown in the image below. ‘Fill’ is equivalent to ‘Border’ at other locations. The field was planted north to south and together, the inbred and hybrid fields are 625 feet north to south. The fields were planted on May 19, 2022. A combination of 10-34-0 (NPK) liquid fertilizer and urea were applied to meet the nitrogen treatment level requirements, as shown below. Urea applications were made on July 8, 2022. Both field layouts denote that planting of the hybrid plots started in the southeast corner of the field; however, the correlations between two replicates of a genotype within a treatment indicate that planting started in the southwest corner of the field. The inbreds were still located directly to the west of the hybrids. In the corrected layout, plot 1001 is in the SW corner of the field, plot 1025 is in the NW corner of the field, the NE corner of the field is a fill plot, and plot 1491 is in the SE corner of the field. The previous crop was dry beans.

Max. of 5 gal/acre of 10-34-0 can be applied in furrow (1 gal = 1.16 lbs N; 3.95 lbs P)								
N&P in 5 gal.liq fert.								
N (lbs)	5.8							
P (lbs)	19.75							
			*5 gal (10-34-0) liq. product					
Trials	N-trmts lbs/a	soil (N1)	*N2	*P	Extra N	Urea/a (N.46%)	Area	Urea needed
Hyb_N1	75	18	5.8	19.75	51.2	111	43750	112
Hyb_N2	150	18	5.8	19.75	126.2	274	43750	276
Hyb_N3	225	18	5.8	19.75	201.2	437	43750	439
Inbred-full yld	250	18	5.8	19.75	226.2	492	37500	423
							Sub-total	1250
Mint-fertility	200	91			109	237	8640	47
							Sub-total	1297
					Total urea to buy		+20%	1556

Trials	N (lbs/a)	Area	Urea to apply (lbs)
Hyb_N1	75	70'x625'	112
Hyb_N2	150	70'x625'	276
Hyb_N3	225	70'x625'	439
Inbred-full yld	250	60'x620'	423
Mint-fertility	200		50
Total urea to buy w. 20% extra			1600

Inbred HIPS field

The plots were 10 foot (7.5 feet planted), 2-row plots. This field was very weedy according to Ramesh.

***Rotated Planted North to South **NORTH**

	ROW	1	2	3	4	5	6	7	8	9	10	11	12	13	14	15	16
	67	B	B	B	B	B	B	B	B	B	B	B	B	B	B	B	B
RANGE	66	B	B	B	B	B	B	B	B	B	B	B	B	B	B	B	B
	65	B	B	B	B	B	B	B	B	B	B	B	B	B	B	B	B
	64	B	B	**Fill**	**683**	**682**	**559**	**558**	**435**	**434**	**311**	**310**	**187**	**186**	**63**	**62**	B
	63	B	B	**Fill**	**684**	**681**	**560**	**557**	**436**	**433**	**312**	**309**	**188**	**185**	**64**	**61**	B
	62	B	B	**Fill**	**685**	**680**	**561**	**556**	**437**	**432**	**313**	**308**	**189**	**184**	**65**	**60**	B
	61	B	B	**Fill**	**686**	**679**	**562**	**555**	**438**	**431**	**314**	**307**	**190**	**183**	**66**	**59**	B
	60	B	B	**Fill**	**687**	**678**	**563**	**554**	**439**	**430**	**315**	**306**	**191**	**182**	**67**	**58**	B
	59	B	B	**Fill**	**688**	**677**	**564**	**553**	**440**	**429**	**316**	**305**	**192**	**181**	**68**	**57**	B
	58	B	B	**Fill**	**689**	**676**	**565**	**552**	**441**	**428**	**317**	**304**	**193**	**180**	**69**	**56**	B
	57	B	B	**Fill**	**690**	**675**	**566**	**551**	**442**	**427**	**318**	**303**	**194**	**179**	**70**	**55**	B
	56	B	B	**Fill**	**691**	**674**	**567**	**550**	**443**	**426**	**319**	**302**	**195**	**178**	**71**	**54**	B
**West**	55	B	B	**Fill**	**692**	**673**	**568**	**549**	**444**	**425**	**320**	**301**	**196**	**177**	**72**	**53**	B
	54	B	B	**Fill**	**693**	**672**	**569**	**548**	**445**	**424**	**321**	**300**	**197**	**176**	**73**	**52**	B
	53	B	B	**Fill**	**694**	**671**	**570**	**547**	**446**	**423**	**322**	**299**	**198**	**175**	**74**	**51**	B
	52	B	B	**Fill**	**695**	**670**	**571**	**546**	**447**	**422**	**323**	**298**	**199**	**174**	**75**	**50**	B
	51	B	B	**Fill**	**696**	**669**	**572**	**545**	**448**	**421**	**324**	**297**	**200**	**173**	**76**	**49**	B
	50	B	B	**Fill**	**697**	**668**	**573**	**544**	**449**	**420**	**325**	**296**	**201**	**172**	**77**	**48**	B
	49	B	B	**Fill**	**698**	**667**	**574**	**543**	**450**	**419**	**326**	**295**	**202**	**171**	**78**	**47**	B
	48	B	B	**Fill**	**699**	**666**	**575**	**542**	**451**	**418**	**327**	**294**	**203**	**170**	**79**	**46**	B
	47	B	B	**Fill**	**700**	**665**	**576**	**541**	**452**	**417**	**328**	**293**	**204**	**169**	**80**	**45**	B
	46	B	B	**Fill**	**701**	**664**	**577**	**540**	**453**	**416**	**329**	**292**	**205**	**168**	**81**	**44**	B
	45	B	B	**Fill**	**702**	**663**	**578**	**539**	**454**	**415**	**330**	**291**	**206**	**167**	**82**	**43**	B
	44	B	B	**Fill**	**703**	**662**	**579**	**538**	**455**	**414**	**331**	**290**	**207**	**166**	**83**	**42**	B
	43	B	B	**Fill**	**704**	**661**	**580**	**537**	**456**	**413**	**332**	**289**	**208**	**165**	**84**	**41**	B
	42	B	B	**Fill**	**705**	**660**	**581**	**536**	**457**	**412**	**333**	**288**	**209**	**164**	**85**	**40**	B
	41	B	B	**Fill**	**706**	**659**	**582**	**535**	**458**	**411**	**334**	**287**	**210**	**163**	**86**	**39**	B
	40	B	B	**Fill**	**707**	**658**	**583**	**534**	**459**	**410**	**335**	**286**	**211**	**162**	**87**	**38**	B
RANGE	39	B	B	**Fill**	**708**	**657**	**584**	**533**	**460**	**409**	**336**	**285**	**212**	**161**	**88**	**37**	B
	38	B	B	**Fill**	**709**	**656**	**585**	**532**	**461**	**408**	**337**	**284**	**213**	**160**	**89**	**36**	B
	37	B	B	**Fill**	**710**	**655**	**586**	**531**	**462**	**407**	**338**	**283**	**214**	**159**	**90**	**35**	B
	36	B	B	**Fill**	**711**	**654**	**587**	**530**	**463**	**406**	**339**	**282**	**215**	**158**	**91**	**34**	B
	35	B	B	**Fill**	**712**	**653**	**588**	**529**	**464**	**405**	**340**	**281**	**216**	**157**	**92**	**33**	B
	34	B	B	**Fill**	**713**	**652**	**589**	**528**	**465**	**404**	**341**	**280**	**217**	**156**	**93**	**32**	B
	33	B	B	**Fill**	**714**	**651**	**590**	**527**	**466**	**403**	**342**	**279**	**218**	**155**	**94**	**31**	B
	32	B	B	**Fill**	**715**	**650**	**591**	**526**	**467**	**402**	**343**	**278**	**219**	**154**	**95**	**30**	B
	31	B	B	**Fill**	**716**	**649**	**592**	**525**	**468**	**401**	**344**	**277**	**220**	**153**	**96**	**29**	B
	30	B	B	**Fill**	**717**	**648**	**593**	**524**	**469**	**400**	**345**	**276**	**221**	**152**	**97**	**28**	B
	29	B	B	**Fill**	**718**	**647**	**594**	**523**	**470**	**399**	**346**	**275**	**222**	**151**	**98**	**27**	B
	28	B	B	**Fill**	**719**	**646**	**595**	**522**	**471**	**398**	**347**	**274**	**223**	**150**	**99**	**26**	B
	27	B	B	**Fill**	**720**	**645**	**596**	**521**	**472**	**397**	**348**	**273**	**224**	**149**	**100**	**25**	B
	26	B	B	**Fill**	**721**	**644**	**597**	**520**	**473**	**396**	**349**	**272**	**225**	**148**	**101**	**24**	B
	25	B	B	**Fill**	**722**	**643**	**598**	**519**	**474**	**395**	**350**	**271**	**226**	**147**	**102**	**23**	B
	24	B	B	**Fill**	**723**	**642**	**599**	**518**	**475**	**394**	**351**	**270**	**227**	**146**	**103**	**22**	B
	23	B	B	**Fill**	**724**	**641**	**600**	**517**	**476**	**393**	**352**	**269**	**228**	**145**	**104**	**21**	B
	22	B	B	**Fill**	**725**	**640**	**601**	**516**	**477**	**392**	**353**	**268**	**229**	**144**	**105**	**20**	B
	21	B	B	**Fill**	**726**	**639**	**602**	**515**	**478**	**391**	**354**	**267**	**230**	**143**	**106**	**19**	B
	20	B	B	**Fill**	**727**	**638**	**603**	**514**	**479**	**390**	**355**	**266**	**231**	**142**	**107**	**18**	B
	19	B	B	**Fill**	**728**	**637**	**604**	**513**	**480**	**389**	**356**	**265**	**232**	**141**	**108**	**17**	B
	18	B	B	**760**	**729**	**636**	**605**	**512**	**481**	**388**	**357**	**264**	**233**	**140**	**109**	**16**	B
	17	B	B	**759**	**730**	**635**	**606**	**511**	**482**	**387**	**358**	**263**	**234**	**139**	**110**	**15**	B
	16	B	B	**758**	**731**	**634**	**607**	**510**	**483**	**386**	**359**	**262**	**235**	**138**	**111**	**14**	B
	15	B	B	**757**	**732**	**633**	**608**	**509**	**484**	**385**	**360**	**261**	**236**	**137**	**112**	**13**	B
	14	B	B	**756**	**733**	**632**	**609**	**508**	**485**	**384**	**361**	**260**	**237**	**136**	**113**	**12**	B
	13	B	B	**755**	**734**	**631**	**610**	**507**	**486**	**383**	**362**	**259**	**238**	**135**	**114**	**11**	B
	12	B	B	**754**	**735**	**630**	**611**	**506**	**487**	**382**	**363**	**258**	**239**	**134**	**115**	**10**	B
	11	B	B	**753**	**736**	**629**	**612**	**505**	**488**	**381**	**364**	**257**	**240**	**133**	**116**	**9**	B
	10	B	B	**752**	**737**	**628**	**613**	**504**	**489**	**380**	**365**	**256**	**241**	**132**	**117**	**8**	B
	9	B	B	**751**	**738**	**627**	**614**	**503**	**490**	**379**	**366**	**255**	**242**	**131**	**118**	**7**	B
	8	B	B	**750**	**739**	**626**	**615**	**502**	**491**	**378**	**367**	**254**	**243**	**130**	**119**	**6**	B
	7	B	B	**749**	**740**	**625**	**616**	**501**	**492**	**377**	**368**	**253**	**244**	**129**	**120**	**5**	B
	6	B	B	**748**	**741**	**624**	**617**	**500**	**493**	**376**	**369**	**252**	**245**	**128**	**121**	**4**	B
	5	B	B	**747**	**742**	**623**	**618**	**499**	**494**	**375**	**370**	**251**	**246**	**127**	**122**	**3**	B
	4	B	B	**746**	**743**	**622**	**619**	**498**	**495**	**374**	**371**	**250**	**247**	**126**	**123**	**2**	B
	3	B	B	**745**	**744**	**621**	**620**	**497**	**496**	**373**	**372**	**249**	**248**	**125**	**124**	**1**	B
	2	B	B	B	B	B	B	B	B	B	B	B	B	B	B	B	B
	1	B	B	B	B	B	B	B	B	B	B	B	B	B	B	B	B
		1	2	3	4	5	6	7	8	9	10	11	12	13	14	15	16

Hybrid HIPS field

Based on yield data and grain protein content, it was determined that the labels in the QR codes for the high and low nitrogen treatments were reversed. The QR codes in the data reflect the content of the original QR codes, and the nitrogen treatment variable reflects the actual level of nitrogen the plot received. The plots were 25 foot (22.5 feet planted), 4-row plots and the middle two rows were harvested.

***Rotated		Planted North to South (Rows are vertical in this map)																NORTH												
		ROW																												
		1	2	3	4	5	6	7	8	9	10	11	12	13	14	15	16	17	18	19	20	21	22	23	24	25	26	27	28	Rang
	30	Bord	Bord	Bord	Bord	Bord	Bord	Bord	Bord	Bord	Bord	Bord	Bord	Bord	Bord	Bord	Bord	Bord	Bord	Bord	Bord	Bord	Bord	Bord	Bord	Bord	Bord	Bord	Bord	
RANGE	29	Bord	Bord	Bord	Bord	Bord	Bord	Bord	Bord	Bord	Bord	Bord	Bord	Bord	Bord	Bord	Bord	Bord	Bord	Bord	Bord	Bord	Bord	Bord	Bord	Bord	Bord	Bord	Bord	
	28	Bord	Bord	Bord	Bord	Bord	Bord	Bord	Bord	Bord	Bord	Bord	Bord	Bord	Bord	Bord	Bord	Bord	Bord	Bord	Bord	Bord	Bord	Bord	Bord	Bord	Bord	Bord	Bord	
	27	Fill	1490	1465	1440	1415	1390	1365	Bord	Bord	Bord	Fill	1320	1295	1270	1245	1220	1195	Bord	Bord	Fill	1150	1125	1100	1075	1050	1025	Bord	Bord	
	26	Fill	1489	1464	1439	1414	1389	1364	Bord	Bord	Bord	Fill	1319	1294	1269	1244	1219	1194	Bord	Bord	Fill	1149	1124	1099	1074	1049	1024	Bord	Bord	
	25	Fill	1488	1463	1438	1413	1388	1363	Bord	Bord	Bord	Fill	1318	1293	1268	1243	1218	1193	Bord	Bord	Fill	1148	1123	1098	1073	1048	1023	Bord	Bord	
	24	Fill	1487	1462	1437	1412	1387	1362	Bord	Bord	Bord	Fill	1317	1292	1267	1242	1217	1192	Bord	Bord	Fill	1147	1122	1097	1072	1047	1022	Bord	Bord	
	23	Fill	1486	1461	1436	1411	1386	1361	Bord	Bord	Bord	Fill	1316	1291	1266	1241	1216	1191	Bord	Bord	Fill	1146	1121	1096	1071	1046	1021	Bord	Bord	
	22	1510	1485	1460	1435	1410	1385	1360	Bord	Bord	Bord	1340	1315	1290	1265	1240	1215	1190	Bord	Bord	1170	1145	1120	1095	1070	1045	1020	Bord	Bord	
	21	1509	1484	1459	1434	1409	1384	1359	Bord	Bord	Bord	1339	1314	1289	1264	1239	1214	1189	Bord	Bord	1169	1144	1119	1094	1069	1044	1019	Bord	Bord	
	20	1508	1483	1458	1433	1408	1383	1358	Bord	Bord	Bord	1338	1313	1288	1263	1238	1213	1188	Bord	Bord	1168	1143	1118	1093	1068	1043	1018	Bord	Bord	
	19	1507	1482	1457	1432	1407	1382	1357	Bord	Bord	Bord	1337	1312	1287	1262	1237	1212	1187	Bord	Bord	1167	1142	1117	1092	1067	1042	1017	Bord	Bord	
West	18	1506	1481	1456	1431	1406	1381	1356	Bord	Bord	Bord	1336	1311	1286	1261	1236	1211	1186	Bord	Bord	1166	1141	1116	1091	1066	1041	1016	Bord	Bord	
	17	1505	1480	1455	1430	1405	1380	1355	Bord	Bord	Bord	1335	1310	1285	1260	1235	1210	1185	Bord	Bord	1165	1140	1115	1090	1065	1040	1015	Bord	Bord	
	16	1504	1479	1454	1429	1404	1379	1354	Bord	Bord	Bord	1334	1309	1284	1259	1234	1209	1184	Bord	Bord	1164	1139	1114	1089	1064	1039	1014	Bord	Bord	
	15	1503	1478	1453	1428	1403	1378	1353	Bord	Bord	Bord	1333	1308	1283	1258	1233	1208	1183	Bord	Bord	1163	1138	1113	1088	1063	1038	1013	Bord	Bord	
	14	1502	1477	1452	1427	1402	1377	1352	Bord	Bord	Bord	1332	1307	1282	1257	1232	1207	1182	Bord	Bord	1162	1137	1112	1087	1062	1037	1012	Bord	Bord	
	13	1501	1476	1451	1426	1401	1376	1351	Bord	Bord	Bord	1331	1306	1281	1256	1231	1206	1181	Bord	Bord	1161	1136	1111	1086	1061	1036	1011	Bord	Bord	
	12	1500	1475	1450	1425	1400	1375	1350	Bord	Bord	Bord	1330	1305	1280	1255	1230	1205	1180	Bord	Bord	1160	1135	1110	1085	1060	1035	1010	Bord	Bord	
	11	1499	1474	1449	1424	1399	1374	1349	Bord	Bord	Bord	1329	1304	1279	1254	1229	1204	1179	Bord	Bord	1159	1134	1109	1084	1059	1034	1009	Bord	Bord	
	10	1498	1473	1448	1423	1398	1373	1348	Bord	Bord	Bord	1328	1303	1278	1253	1228	1203	1178	Bord	Bord	1158	1133	1108	1083	1058	1033	1008	Bord	Bord	
	9	1497	1472	1447	1422	1397	1372	1347	Bord	Bord	Bord	1327	1302	1277	1252	1227	1202	1177	Bord	Bord	1157	1132	1107	1082	1057	1032	1007	Bord	Bord	
	8	1496	1471	1446	1421	1396	1371	1346	Bord	Bord	Bord	1326	1301	1276	1251	1226	1201	1176	Bord	Bord	1156	1131	1106	1081	1056	1031	1006	Bord	Bord	
	7	1495	1470	1445	1420	1395	1370	1345	Bord	Bord	Bord	1325	1300	1275	1250	1225	1200	1175	Bord	Bord	1155	1130	1105	1080	1055	1030	1005	Bord	Bord	
	6	1494	1469	1444	1419	1394	1369	1344	Bord	Bord	Bord	1324	1299	1274	1249	1224	1199	1174	Bord	Bord	1154	1129	1104	1079	1054	1029	1004	Bord	Bord	
	5	1493	1468	1443	1418	1393	1368	1343	Bord	Bord	Bord	1323	1298	1273	1248	1223	1198	1173	Bord	Bord	1153	1128	1103	1078	1053	1028	1003	Bord	Bord	
	4	1492	1467	1442	1417	1392	1367	1342	Bord	Bord	Bord	1322	1297	1272	1247	1222	1197	1172	Bord	Bord	1152	1127	1102	1077	1052	1027	1002	Bord	Bord	
	3	1491	1466	1441	1416	1391	1366	1341	Bord	Bord	Bord	1321	1296	1271	1246	1221	1196	1171	Bord	Bord	1151	1126	1101	1076	1051	1026	1001	Bord	Bord	
RANGE	2	Bord	Bord	Bord	Bord	Bord	Bord	Bord	Bord	Bord	Bord	Bord	Bord	Bord	Bord	Bord	Bord	Bord	Bord	Bord	Bord	Bord	Bord	Bord	Bord	Bord	Bord	Bord	Bord	
	1	Bord	Bord	Bord	Bord	Bord	Bord	Bord	Bord	Bord	Bord	Bord	Bord	Bord	Bord	Bord	Bord	Bord	Bord	Bord	Bord	Bord	Bord	Bord	Bord	Bord	Bord	Bord	Bord	
		1	2	3	4	5	6	7	8	9	10	11	12	13	14	15	16	17	18	19	20	21	22	23	24	25	26	27	28	
			ROW																											
		High	High	High	High	High	High	High	Bord	Bord	Bord	Medium	Medium	Medium	Medium	Medium	Medium	Medium	Bord	Bord	Low	Low	Low	Low	Low	Low	Low			

Crawfordsville 2022

These fields were planted on May 11, 2022 and harvested on October 7, 2022. Both the inbred and hybrid fields had 3 nitrogen treatments (High, Medium, and Low). The field coordinates were 41.199066, -91.486991. The nitrogen treatments were applied on June 2, 2022 using 32% UAN.

Field A - Low and High Nitrogen

Low nitrogen is to the west in these maps. The previous crop was soybeans.

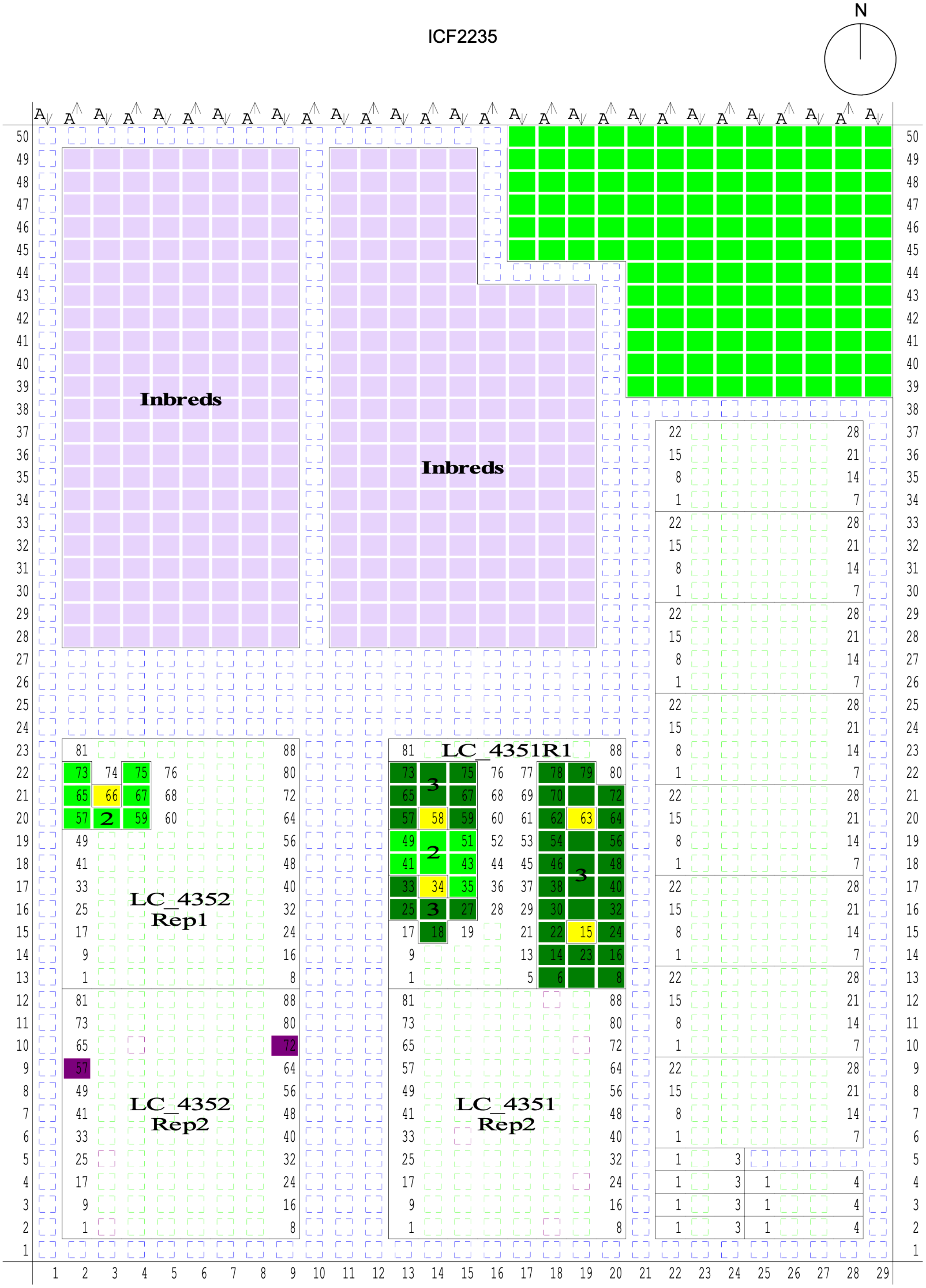
ICF2235
N
Inbreds
Inbreds
LC_4351R1
LC_4352
Rep1
LC_4352
Rep2
LC_4351
Rep2

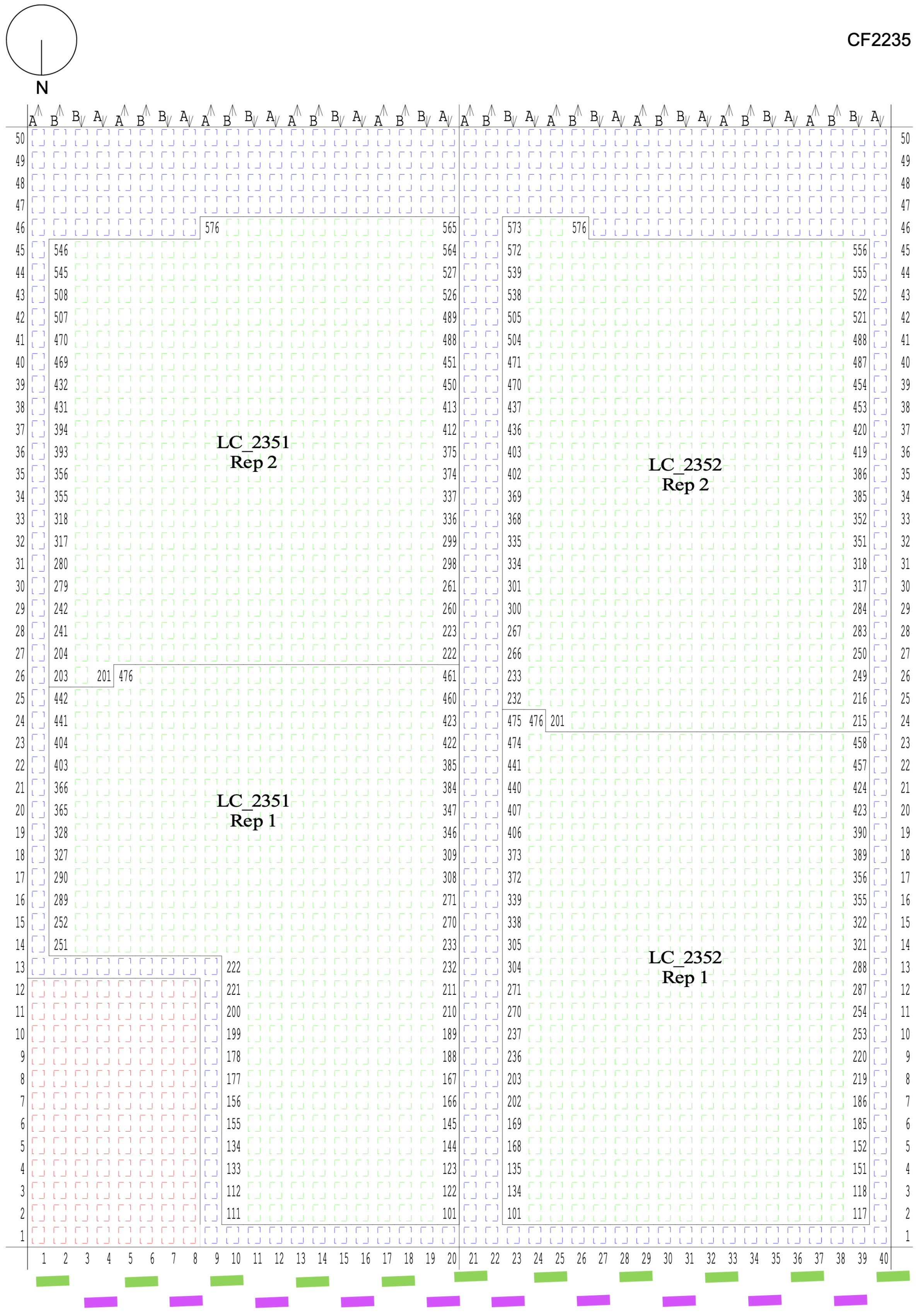

Plot Numbers

ISU.IA.Crawfordsville

Passes in purple planted farther north -LC

Field B - Medium Nitrogen

The previous crop was soybeans.

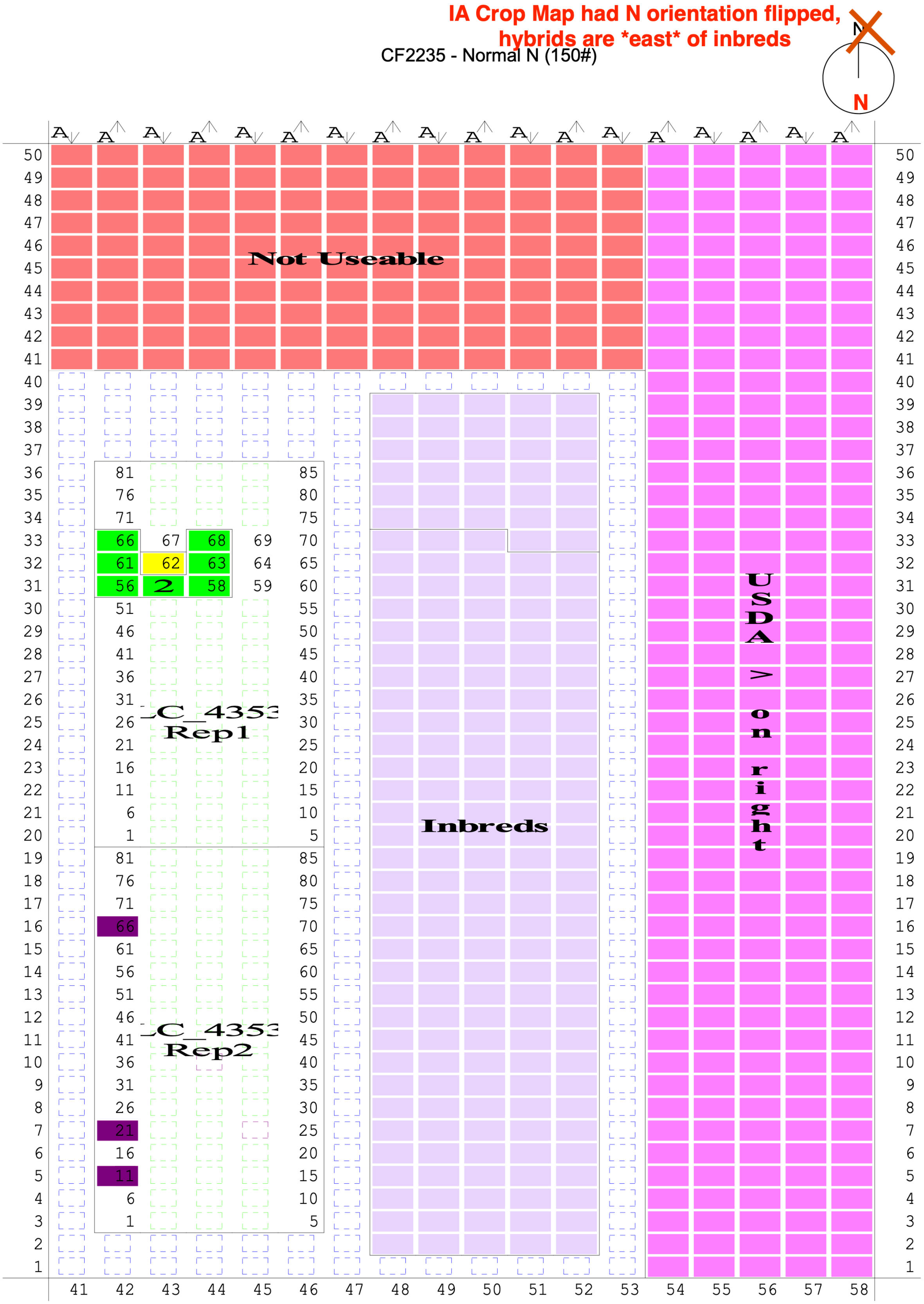
IA Crop Map had N orientation flipped,
hybrids are *east* of inbreds
CF2235 - Normal N (150#)
N
N
Not Useable
Rep1
Rep2
Inbreds
USDA > on right

CF2235.10ft - Normal N (150#)

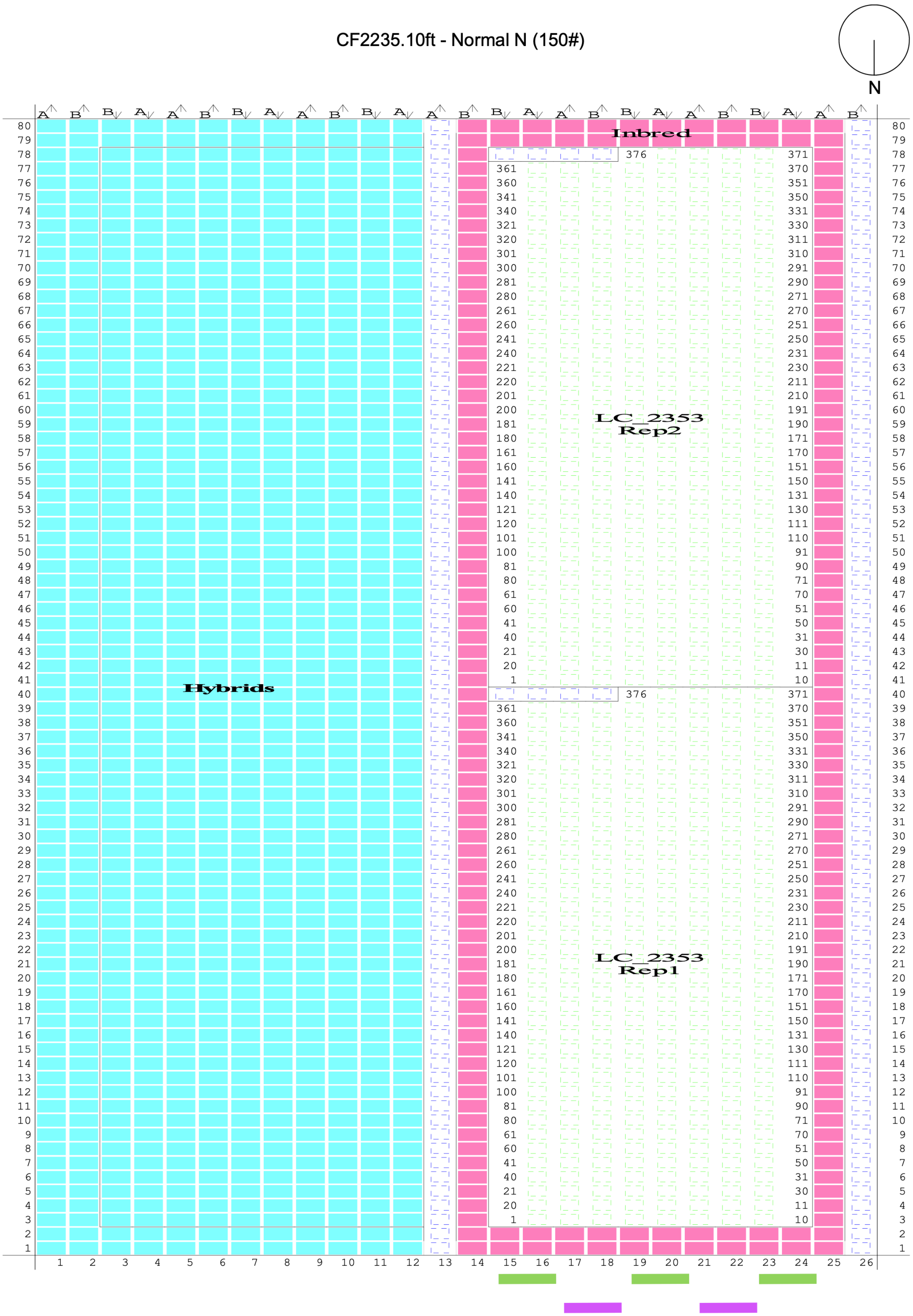

Passes in purple planted farther north -LC

Ames 2022

These fields were planted on May 23, 2022 and harvested on October 16, 2022. Both the inbred and hybrid fields had 3 nitrogen treatments (High, Medium, and Low). The High and Medium nitrogen treatments for both hybrids and inbreds were located in the B1 field. The Low nitrogen treatments for both hybrids and inbreds were located in the E1 field.

B1

This field was planted on May 22, 2022. Nitrogen was applied using urea and 32% UAN on May 17, 2022, and June 1, 2022. The field coordinates were 42.015354, -93.732519. The previous crop was corn.

CF2223.F1

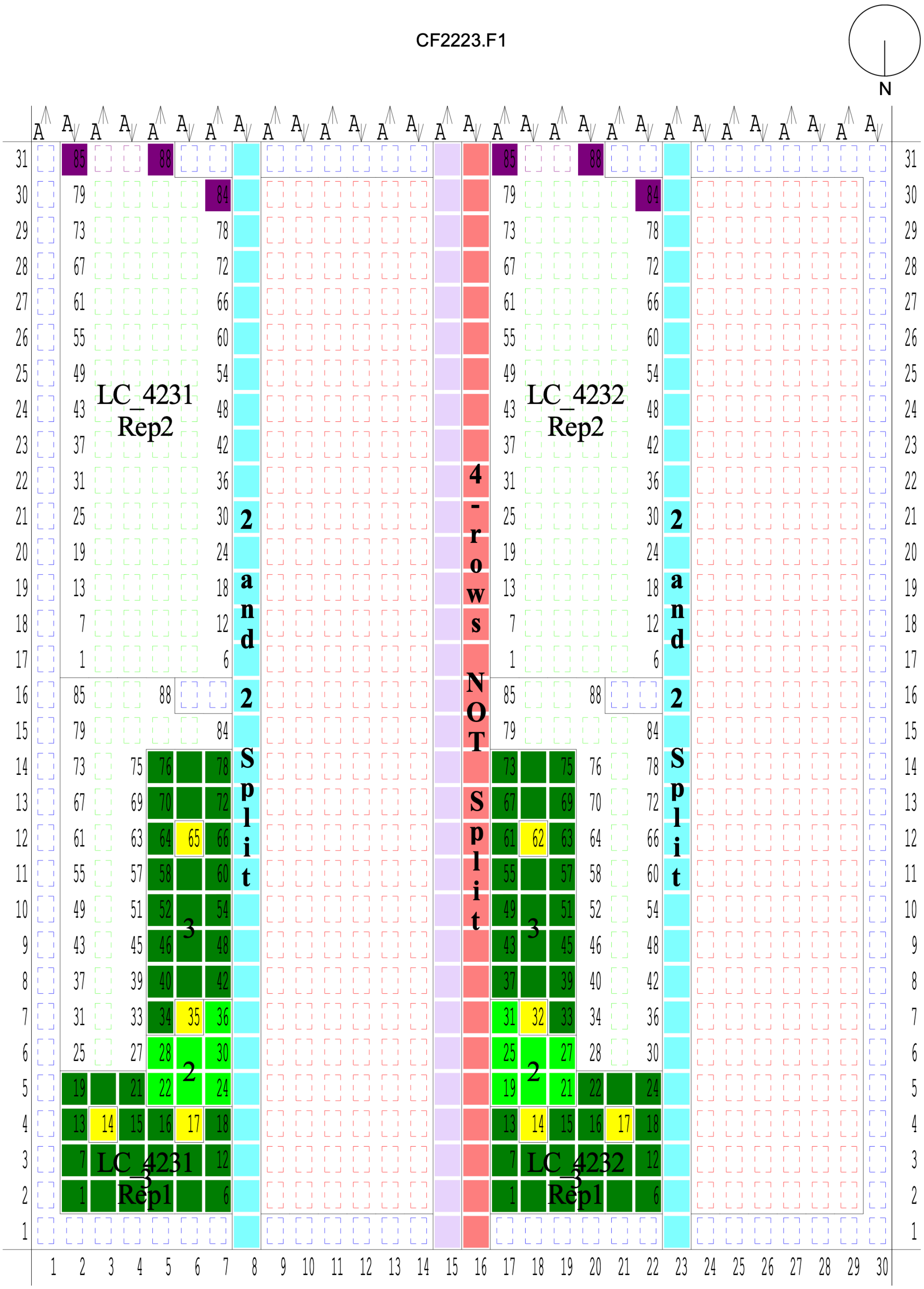
N
LC_4231
Rep2
LC_4232
Rep2
LC_4231
Rep1
LC_4232
Rep1
2 and 2 Split
4-rows NOT Split

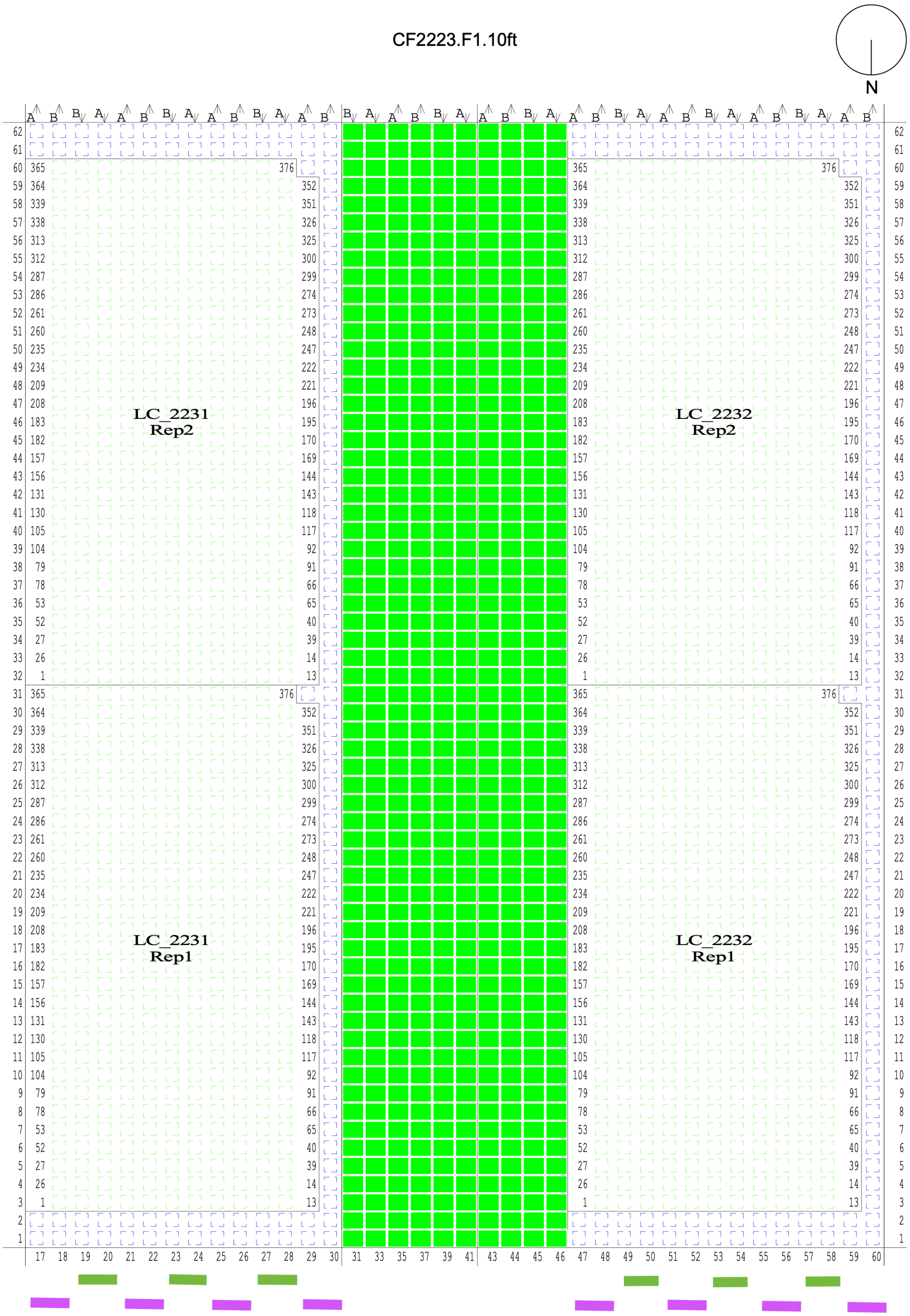

Passes in purple planted farther north -LC

E1

This field was planted on May 23, 2022. Nitrogen was applied using urea on May 21, 2022. The field coordinates were 42.012376, -93.737301. The previous crop was soybeans.

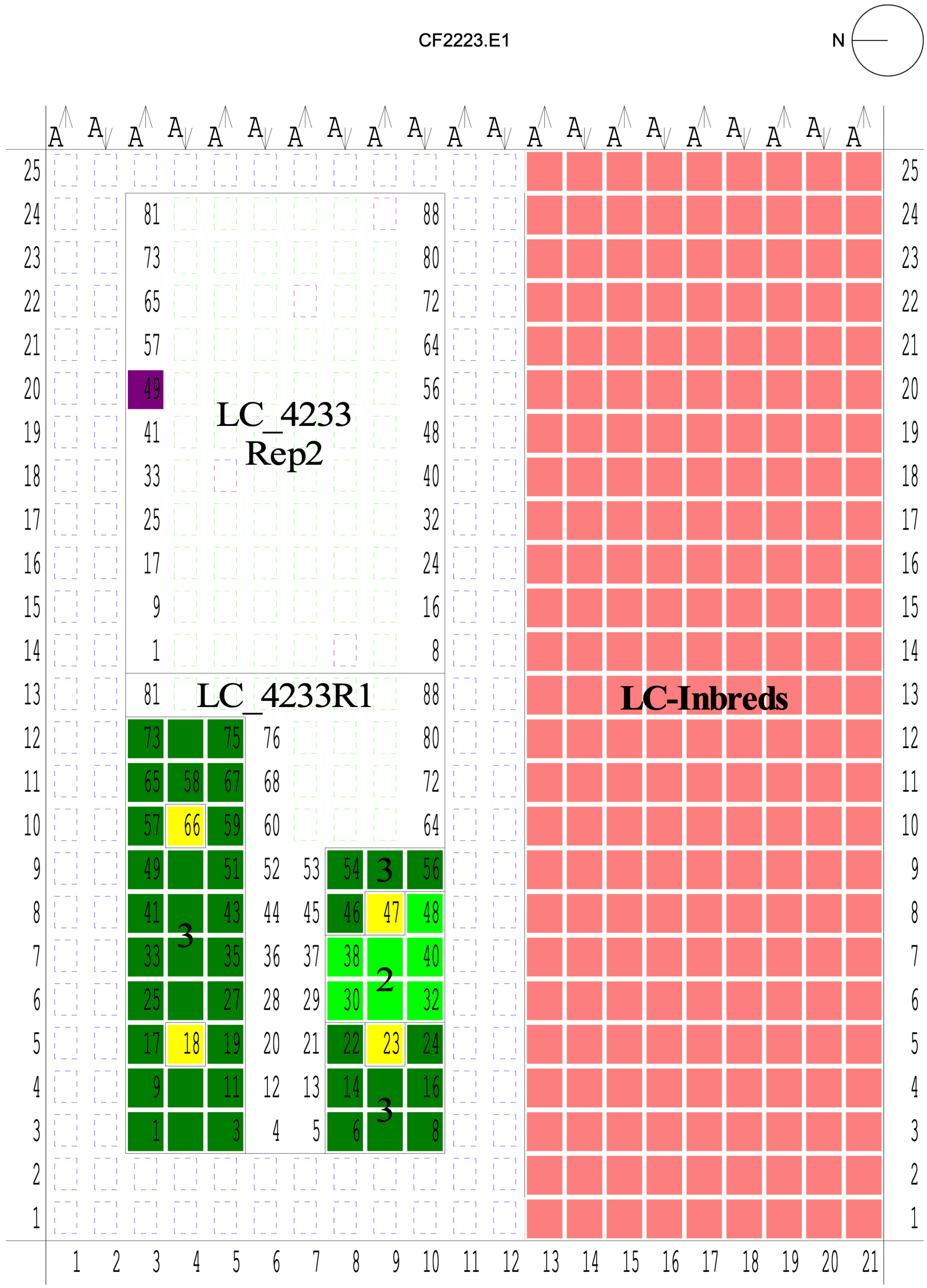
CF2223.E1
N
LC_4233
Rep2
LC_4233R1
LC-Inbreds

CF2223.E1.10ft

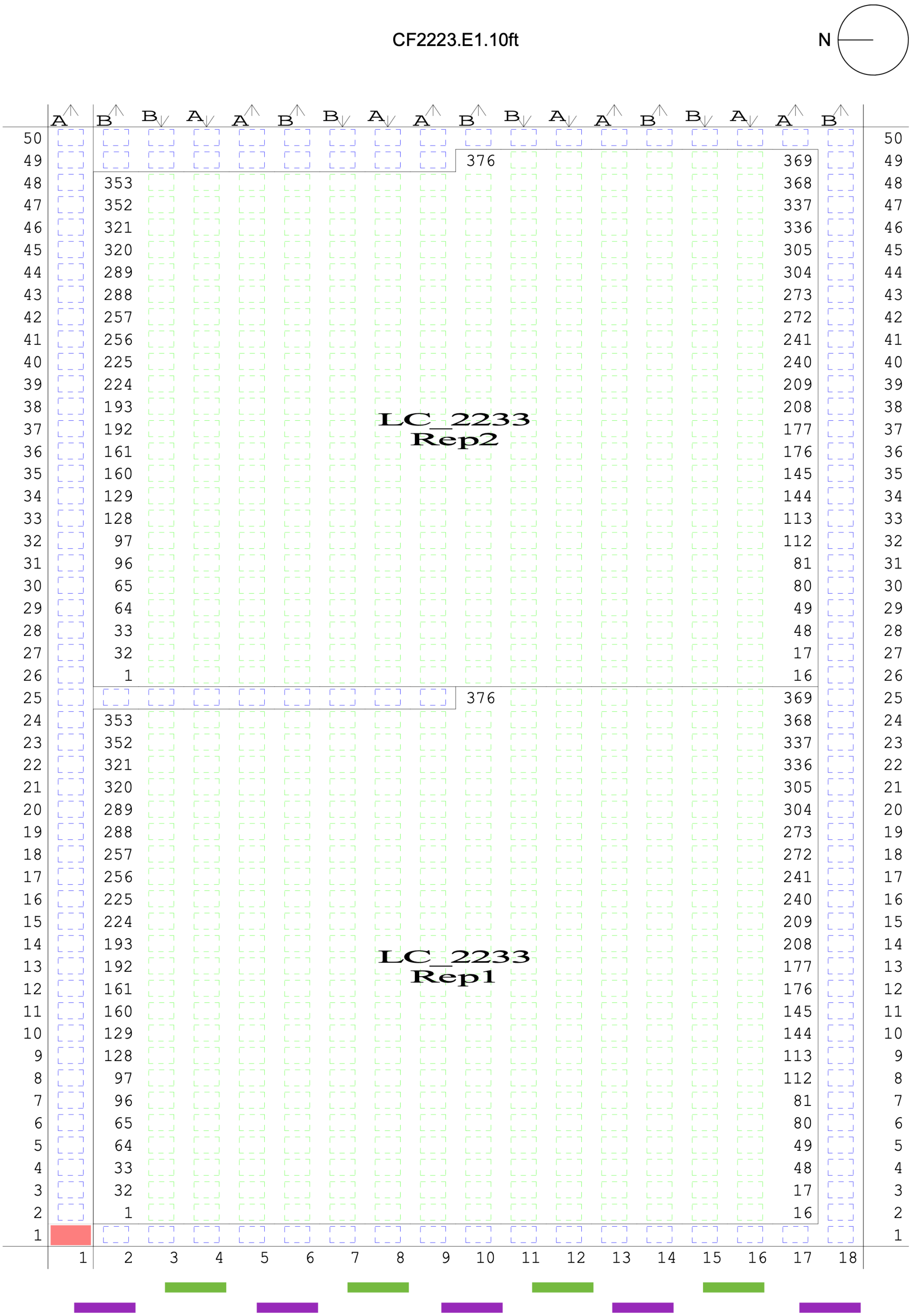

Passes in purple planted farther west -LC

North Platte 2023

Plots were 4 row plots with 30” row spacing and were 20 feet center to center, including 17.5 feet of plants and 2.5 feet of alley. Nitrogen was applied on 6/15/23, 150 lb/a N as 32-0-0 surface applied with Y-Drop applicator. The previous crop was was soybean. The field had 8 underground irrigation zones randomized within each of 4 blocks. 5 of the zones were set to irrigate with 4.5 inches of irrigation over the growing season, and 3 of the zones applied 0 inches of irrigation. The field coordinates were 41.086705°, -100.775034° and plots were planted on May 10, 2023. Hand harvesting was done on October 11, 2023, and mechanical harvest was done on October 19, 2023.

There was significant hail damage the night of July 22-23, during the middle of the tasseling period.

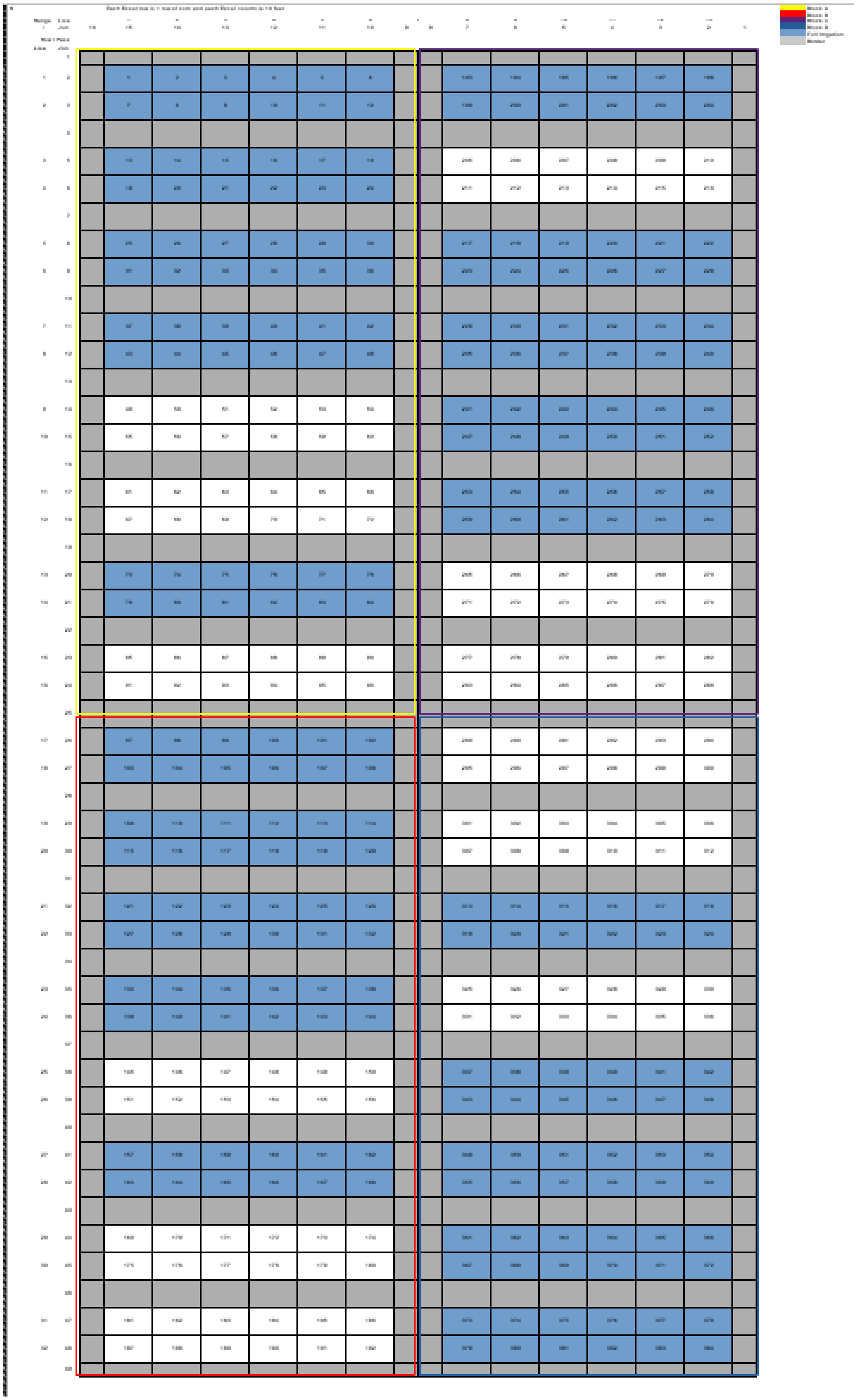

Lincoln 2023

All plots had 30 inch row spacing and 2.5 ft alleys. The field coordinates were 40.859683°, -96.596310°. The previous crop was soybeans.

Hybrid HIPS field

The field was planted on May 16, 2023. 3 rates of nitrogen (75, 150, and 225 lbs/acre) were applied as liquid urea. Plots were 4-row plots, 20 feet center to center. Hand harvest was completed on September 25, 2023 and mechanical harvesting was done on October 23, 2023.

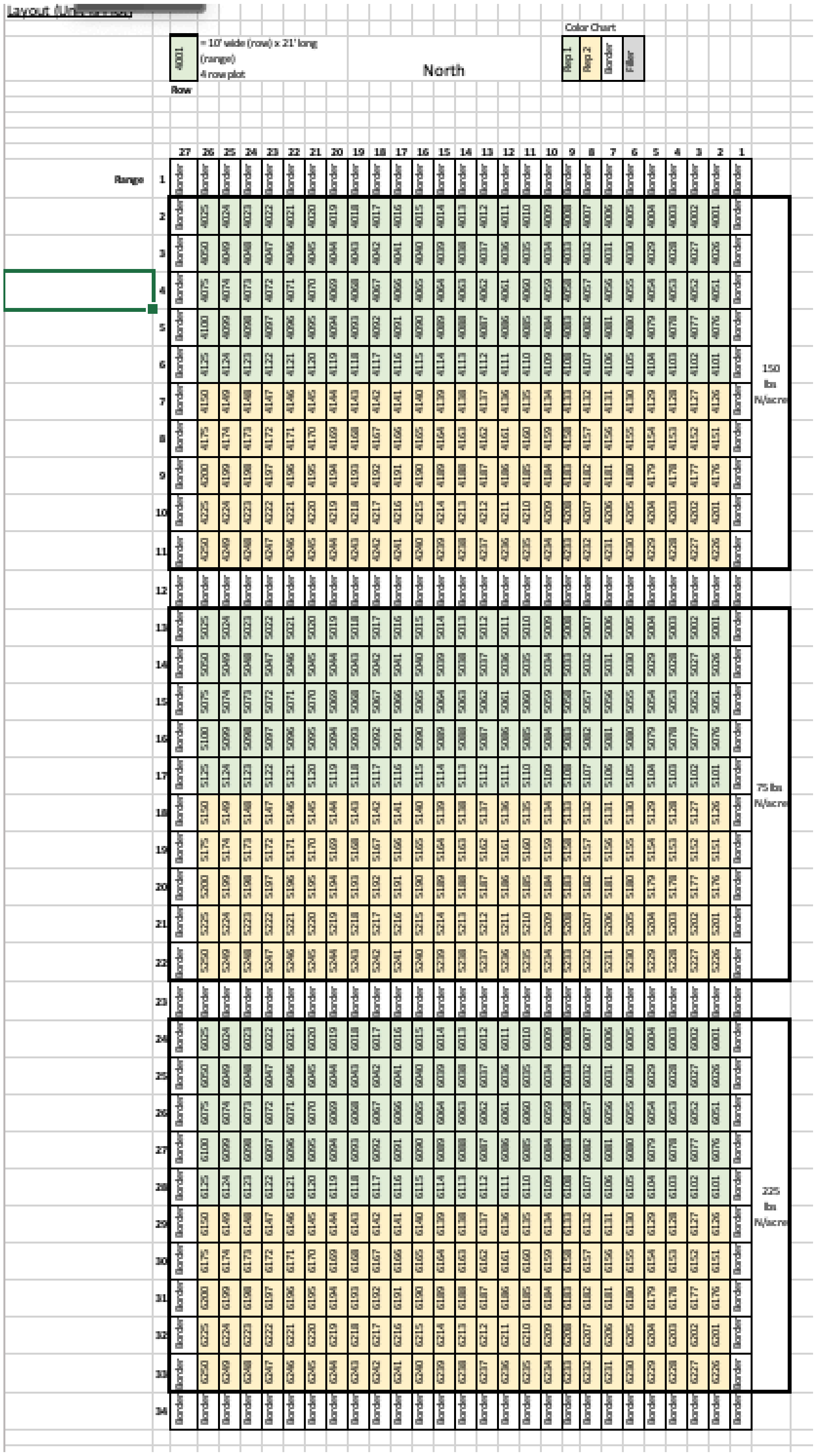

Inbred HIPS field

The field was planted on May 9, 2023. A single rate of nitrogen (150 lbs/acre) was applied as liquid urea. Plots were 2-row plots. Hand harvest was completed on September 30, 2023. Plots were 10 feet center to center with 2.5 ft alleys.

Layout (Unit is Plot) North

1001	= 5' wide (row) x 10' long (range) 2 row plot
Row	

Color Chart: Rep 1, Rep 2, Border, Filler

nbred HIPS

		34	33	32	31	30	29	28	27	26	25	24	23	22	21	20	19	18	17	16	15	14	13	12	11	10	9	8	7	6	5	4	3	2	1
Range	1	Border	Border	Border	Border	Border	Border	Border	Border	Border	Border	Border	Border	Border	Border	Border	Border	Border	Border	Border	Border	Border	Border	Border	Border	Border	Border	Border	Border	Border	Border	Border	Border	Border	Border
	2	Border	2016	2015	2014	2013	2012	2011	2010	2009	2008	2007	2006	2005	2004	2003	2002	2001	1016	1015	1014	1013	1012	1011	1010	1009	1008	1007	1006	1005	1004	1003	1002	1001	Border
	3	Border	2032	2031	2030	2029	2028	2027	2026	2025	2024	2023	2022	2021	2020	2019	2018	2017	1032	1031	1030	1029	1028	1027	1026	1025	1024	1023	1022	1021	1020	1019	1018	1017	Border
	4	Border	2048	2047	2046	2045	2044	2043	2042	2041	2040	2039	2038	2037	2036	2035	2034	2033	1048	1047	1046	1045	1044	1043	1042	1041	1040	1039	1038	1037	1036	1035	1034	1033	Border
	5	Border	2064	2063	2062	2061	2060	2059	2058	2057	2056	2055	2054	2053	2052	2051	2050	2049	1064	1063	1062	1061	1060	1059	1058	1057	1056	1055	1054	1053	1052	1051	1050	1049	Border
	6	Border	2080	2079	2078	2077	2076	2075	2074	2073	2072	2071	2070	2069	2068	2067	2066	2065	1080	1079	1078	1077	1076	1075	1074	1073	1072	1071	1070	1069	1068	1067	1066	1065	Border
	7	Border	2096	2095	2094	2093	2092	2091	2090	2089	2088	2087	2086	2085	2084	2083	2082	2081	1096	1095	1094	1093	1092	1091	1090	1089	1088	1087	1086	1085	1084	1083	1082	1081	Border
	8	Border	2112	2111	2110	2109	2108	2107	2106	2105	2104	2103	2102	2101	2100	2099	2098	2097	1112	1111	1110	1109	1108	1107	1106	1105	1104	1103	1102	1101	1100	1099	1098	1097	Border
	9	Border	2128	2127	2126	2125	2124	2123	2122	2121	2120	2119	2118	2117	2116	2115	2114	2113	1128	1127	1126	1125	1124	1123	1122	1121	1120	1119	1118	1117	1116	1115	1114	1113	Border
	10	Border	2144	2143	2142	2141	2140	2139	2138	2137	2136	2135	2134	2133	2132	2131	2130	2129	1144	1143	1142	1141	1140	1139	1138	1137	1136	1135	1134	1133	1132	1131	1130	1129	Border
	11	Border	2160	2159	2158	2157	2156	2155	2154	2153	2152	2151	2150	2149	2148	2147	2146	2145	1160	1159	1158	1157	1156	1155	1154	1153	1152	1151	1150	1149	1148	1147	1146	1145	Border
	12	Border	2176	2175	2174	2173	2172	2171	2170	2169	2168	2167	2166	2165	2164	2163	2162	2161	1176	1175	1174	1173	1172	1171	1170	1169	1168	1167	1166	1165	1164	1163	1162	1161	Border
	13	Border	2192	2191	2190	2189	2188	2187	2186	2185	2184	2183	2182	2181	2180	2179	2178	2177	1192	1191	1190	1189	1188	1187	1186	1185	1184	1183	1182	1181	1180	1179	1178	1177	Border
	14	Border	2208	2207	2206	2205	2204	2203	2202	2201	2200	2199	2198	2197	2196	2195	2194	2193	1208	1207	1206	1205	1204	1203	1202	1201	1200	1199	1198	1197	1196	1195	1194	1193	Border
	15	Border	2224	2223	2222	2221	2220	2219	2218	2217	2216	2215	2214	2213	2212	2211	2210	2209	1224	1223	1222	1221	1220	1219	1218	1217	1216	1215	1214	1213	1212	1211	1210	1209	Border
	16	Border	2240	2239	2238	2237	2236	2235	2234	2233	2232	2231	2230	2229	2228	2227	2226	2225	1240	1239	1238	1237	1236	1235	1234	1233	1232	1231	1230	1229	1228	1227	1226	1225	Border
	17	Border	2256	2255	2254	2253	2252	2251	2250	2249	2248	2247	2246	2245	2244	2243	2242	2241	1256	1255	1254	1253	1252	1251	1250	1249	1248	1247	1246	1245	1244	1243	1242	1241	Border
	18	Border	2272	2271	2270	2269	2268	2267	2266	2265	2264	2263	2262	2261	2260	2259	2258	2257	1272	1271	1270	1269	1268	1267	1266	1265	1264	1263	1262	1261	1260	1259	1258	1257	Border
	19	Border	2288	2287	2286	2285	2284	2283	2282	2281	2280	2279	2278	2277	2276	2275	2274	2273	1288	1287	1286	1285	1284	1283	1282	1281	1280	1279	1278	1277	1276	1275	1274	1273	Border
	20	Border	2304	2303	2302	2301	2300	2299	2298	2297	2296	2295	2294	2293	2292	2291	2290	2289	1304	1303	1302	1301	1300	1299	1298	1297	1296	1295	1294	1293	1292	1291	1290	1289	Border
	21	Border	2320	2319	2318	2317	2316	2315	2314	2313	2312	2311	2310	2309	2308	2307	2306	2305	1320	1319	1318	1317	1316	1315	1314	1313	1312	1311	1310	1309	1308	1307	1306	1305	Border
	22	Border	2336	2335	2334	2333	2332	2331	2330	2329	2328	2327	2326	2325	2324	2323	2322	2321	1336	1335	1334	1333	1332	1331	1330	1329	1328	1327	1326	1325	1324	1323	1322	1321	Border
	23	Border	2352	2351	2350	2349	2348	2347	2346	2345	2344	2343	2342	2341	2340	2339	2338	2337	1352	1351	1350	1349	1348	1347	1346	1345	1344	1343	1342	1341	1340	1339	1338	1337	Border
	24	Border	2368	2367	2366	2365	2364	2363	2362	2361	2360	2359	2358	2357	2356	2355	2354	2353	1368	1367	1366	1365	1364	1363	1362	1361	1360	1359	1358	1357	1356	1355	1354	1353	Border
	25	Border	2384	2383	2382	2381	2380	2379	2378	2377	2376	2375	2374	2373	2372	2371	2370	2369	1384	1383	1382	1381	1380	1379	1378	1377	1376	1375	1374	1373	1372	1371	1370	1369	Border
	26	Border	2400	2399	2398	2397	2396	2395	2394	2393	2392	2391	2390	2389	2388	2387	2386	2385	1400	1399	1398	1397	1396	1395	1394	1393	1392	1391	1390	1389	1388	1387	1386	1385	Border
	27	Border	Border	Border	Border	Border	Border	Border	Border	Border	Border	Border	Border	Border	Border	Border	Border	Border	Border	Border	Border	Border	Border	Border	Border	Border	Border	Border	Border	Border	Border	Border	Border	Border	Border

Missouri Valley 2023

The field coordinates were 41.671045°, -95.945240°. The row spacing was 30” and the previous crop was soybean. A single nitrogen rate (160 lbs/acre) was applied with NH3. Both inbred and hybrid fields were planted May 2, 2023. Hand harvest for both fields was completed on September 17, 2023.

Hybrid HIPS field

Plots were 20 feet center to center, with 2.5 ft alleys, and 4 rows. Mechanical harvest was completed on September 25, 2023.

Hybrid HIPS (By Plot #)

North

LC2125 - Rep 1 | LC2125 - Rep 2 | LC2125_ER - Rep 1 | LC2125_ER - Rep 2

		Plot Row																				
		1	2	3	4	5	6	7	8	9	10	11	12	13	14	15	16	17	18	19	20	21
Range	42	NA	NA	NA	NA	NA	NA	NA	NA	NA	NA	NA	NA	NA	NA	NA	NA	NA	NA	NA	NA	NA
	41	NA	NA	NA	NA	NA	NA	NA	NA	NA	NA	NA	NA	223	224	225	226	227	228	229	230	231
	40	NA	NA	NA	NA	NA	NA	NA	NA	NA	NA	NA	NA	222	221	220	219	218	217	216	215	214
	39	NA	NA	NA	NA	NA	NA	NA	NA	NA	NA	NA	NA	NA	NA	NA	NA	NA	NA	NA	212	213
	38	NA	NA	NA	NA	NA	NA	NA	NA	NA	NA	NA	NA	NA	NA	NA	NA	NA	NA	NA	211	210
	37	NA	NA	NA	NA	NA	NA	NA	NA	NA	NA	NA	NA	NA	NA	NA	NA	NA	NA	NA	208	209
	36	NA	NA	NA	NA	NA	NA	NA	NA	NA	NA	NA	NA	NA	NA	NA	NA	NA	NA	NA	207	206
	35	NA	NA	NA	NA	NA	NA	NA	NA	NA	NA	NA	NA	NA	NA	NA	NA	NA	NA	NA	204	205
	34	NA	NA	NA	NA	NA	NA	NA	NA	NA	NA	NA	NA	NA	NA	NA	NA	NA	NA	NA	203	202
	33	NA	NA	NA	NA	NA	NA	NA	NA	NA	NA	NA	NA	NA	NA	NA	NA	NA	NA	NA	201	131
	32	NA	NA	NA	NA	NA	NA	NA	NA	NA	NA	NA	NA	NA	NA	NA	NA	NA	NA	NA	129	130
	31	NA	NA	NA	NA	NA	NA	NA	NA	NA	NA	NA	NA	NA	NA	NA	NA	NA	NA	NA	128	127
	30	NA	NA	NA	NA	NA	NA	NA	NA	NA	NA	NA	NA	NA	NA	NA	NA	NA	NA	NA	125	126
	29	NA	NA	NA	NA	NA	NA	NA	NA	NA	NA	NA	NA	NA	NA	NA	NA	NA	NA	NA	124	123
	28	NA	NA	NA	NA	NA	NA	NA	NA	NA	NA	NA	NA	NA	NA	NA	NA	NA	NA	NA	121	122
	27	NA	NA	NA	NA	NA	NA	NA	NA	NA	NA	NA	NA	NA	NA	NA	NA	NA	NA	NA	120	119
	26	NA	NA	NA	NA	NA	NA	NA	NA	NA	NA	NA	NA	NA	NA	NA	NA	NA	NA	NA	117	118
	25	NA	NA	NA	NA	NA	NA	NA	NA	NA	NA	NA	NA	NA	NA	NA	NA	NA	NA	NA	116	115
	24	NA	NA	NA	NA	NA	NA	NA	NA	NA	NA	NA	NA	NA	NA	NA	NA	NA	NA	NA	113	114
	23	NA	NA	NA	NA	NA	NA	NA	NA	NA	NA	NA	NA	NA	NA	NA	NA	NA	NA	NA	112	111
	22	NA	NA	NA	NA	NA	NA	NA	NA	NA	NA	NA	NA	NA	NA	NA	NA	NA	NA	NA	109	110
	21	NA	NA	NA	NA	NA	NA	NA	NA	NA	NA	NA	NA	NA	NA	NA	NA	NA	NA	NA	108	107
	20	NA	NA	NA	NA	NA	NA	NA	NA	NA	NA	NA	NA	NA	NA	NA	NA	NA	NA	NA	105	106
	19	NA	NA	NA	NA	NA	NA	NA	NA	NA	NA	NA	NA	NA	NA	NA	NA	NA	NA	NA	104	103
	18	NA	NA	NA	NA	NA	NA	NA	NA	NA	NA	NA	NA	NA	NA	NA	NA	NA	NA	NA	101	102
	17	NA	181	182	183	184	185	186	187	NA	NA	NA	281	282	283	284	285	286	287	NA	NA	NA
	16	NA	180	179	178	177	176	175	174	173	172	171	280	279	278	277	276	275	274	273	272	271
	15	NA	161	162	163	164	165	166	167	168	169	170	261	262	263	264	265	266	267	268	269	270
	14	NA	160	159	158	157	156	155	154	153	152	151	260	259	258	257	256	255	254	253	252	251
	13	NA	141	142	143	144	145	146	147	148	149	150	241	242	243	244	245	246	247	248	249	250
	12	NA	140	139	138	137	136	135	134	133	132	131	240	239	238	237	236	235	234	233	232	231
	11	NA	121	122	123	124	125	126	127	128	129	130	221	222	223	224	225	226	227	228	229	230
	10	NA	120	119	118	117	116	115	114	113	112	111	220	219	218	217	216	215	214	213	212	211
	9	NA	101	102	103	104	105	106	107	108	109	110	201	202	203	204	205	206	207	208	209	210
	8	NA	NA	NA	NA	NA	NA	NA	NA	NA	NA	NA	NA	NA	NA	NA	NA	NA	NA	NA	NA	NA
	7	NA	NA	NA	NA	NA	NA	NA	NA	NA	NA	NA	NA	NA	NA	NA	NA	NA	NA	NA	NA	NA
	6	NA	NA	NA	NA	NA	NA	NA	NA	NA	NA	NA	NA	NA	NA	NA	NA	NA	NA	NA	NA	NA
	5	NA	NA	NA	NA	NA	NA	NA	NA	NA	NA	NA	NA	NA	NA	NA	NA	NA	NA	NA	NA	NA
	4	NA	NA	NA	NA	NA	NA	NA	NA	NA	NA	NA	NA	NA	NA	NA	NA	NA	NA	NA	NA	NA
	3	NA	NA	NA	NA	NA	NA	NA	NA	NA	NA	NA	NA	NA	NA	NA	NA	NA	NA	NA	NA	NA
Range	2	NA	NA	NA	NA	NA	NA	NA	NA	NA	NA	NA	NA	NA	NA	NA	NA	NA	NA	NA	NA	NA
	1	NA	NA	NA	NA	NA	NA	NA	NA	NA	NA	NA	NA	NA	NA	NA	NA	NA	NA	NA	NA	NA
		1	2	3	4	5	6	7	8	9	10	11	12	13	14	15	16	17	18	19	20	21
		Plot Row																				

Inbred HIPS field

Plots were 10 feet center to center, with 2.5 ft alleys, and 2 rows.

Inbred HIPS (By Plot #)				Rep 2										
				Rep 1										
		Plot Row	1	2	3	4	5	6	7	8	9	10	11	12
		Field Row	1 2	3 4	5 6	7 8	9 10	11 12	13 14	15 16	17 18	19 20	21 22	23 24
		84	NA	NA	NA	NA	NA	NA	NA	NA	NA	NA	NA	NA
	Range	83	NA	NA	NA	NA	NA	NA	NA	NA	NA	NA	NA	NA
		82	NA	Filler	Filler	Filler	Filler	596	595	594	593	592	591	NA
		81	NA	581	582	583	584	585	586	587	588	589	590	NA
		80	NA	580	579	578	577	576	575	574	573	572	571	NA
		79	NA	561	562	563	564	565	566	567	568	569	570	NA
		78	NA	560	559	558	557	556	555	554	553	552	551	NA
		77	NA	541	542	543	544	545	546	547	548	549	550	NA
		76	NA	540	539	538	537	536	535	534	533	532	531	NA
		75	NA	521	522	523	524	525	526	527	528	529	530	NA
		74	NA	520	519	518	517	516	515	514	513	512	511	NA
		73	NA	501	502	503	504	505	506	507	508	509	510	NA
		72	NA	500	499	498	497	496	495	494	493	492	491	NA
		71	NA	481	482	483	484	485	486	487	488	489	490	NA
		70	NA	480	479	478	477	476	475	474	473	472	471	NA
		69	NA	461	462	463	464	465	466	467	468	469	470	NA
		68	NA	460	459	458	457	456	455	454	453	452	451	NA
		67	NA	441	442	443	444	445	446	447	448	449	450	NA
		66	NA	440	439	438	437	436	435	434	433	432	431	NA
		65	NA	421	422	423	424	425	426	427	428	429	430	NA
		64	NA	420	419	418	417	416	415	414	413	412	411	NA
		63	NA	401	402	403	404	405	406	407	408	409	410	NA
		62	NA	400	399	398	397	396	395	394	393	392	391	NA
		61	NA	381	382	383	384	385	386	387	388	389	390	NA
		60	NA	380	379	378	377	376	375	374	373	372	371	NA
		59	NA	361	362	363	364	365	366	367	368	369	370	NA
		58	NA	360	359	358	357	356	355	354	353	352	351	NA
		57	NA	341	342	343	344	345	346	347	348	349	350	NA
		56	NA	340	339	338	337	336	335	334	333	332	331	NA
		55	NA	321	322	323	324	325	326	327	328	329	330	NA
		54	NA	320	319	318	317	316	315	314	313	312	311	NA
		53	NA	301	302	303	304	305	306	307	308	309	310	NA
		52	NA	300	299	298	297	296	295	294	293	292	291	NA
		51	NA	281	282	283	284	285	286	287	288	289	290	NA
		50	NA	280	279	278	277	276	275	274	273	272	271	NA
		49	NA	261	262	263	264	265	266	267	268	269	270	NA
		48	NA	260	259	258	257	256	255	254	253	252	251	NA
		47	NA	241	242	243	244	245	246	247	248	249	250	NA
		46	NA	240	239	238	237	236	235	234	233	232	231	NA
		45	NA	221	222	223	224	225	226	227	228	229	230	NA
		44	NA	220	219	218	217	216	215	214	213	212	211	NA
		43	NA	201	202	203	204	205	206	207	208	209	210	NA
		42	NA	Filler	Filler	Filler	Filler	496	495	494	493	492	491	NA
		41	NA	481	482	483	484	485	486	487	488	489	490	NA
		40	NA	480	479	478	477	476	475	474	473	472	471	NA
		39	NA	461	462	463	464	465	466	467	468	469	470	NA
		38	NA	460	459	458	457	456	455	454	453	452	451	NA
		37	NA	441	442	443	444	445	446	447	448	449	450	NA
		36	NA	440	439	438	437	436	435	434	433	432	431	NA
		35	NA	421	422	423	424	425	426	427	428	429	430	NA
		34	NA	420	419	418	417	416	415	414	413	412	411	NA
		33	NA	401	402	403	404	405	406	407	408	409	410	NA
		32	NA	400	399	398	397	396	395	394	393	392	391	NA
		31	NA	381	382	383	384	385	386	387	388	389	390	NA
		30	NA	380	379	378	377	376	375	374	373	372	371	NA
		29	NA	361	362	363	364	365	366	367	368	369	370	NA
		28	NA	360	359	358	357	356	355	354	353	352	351	NA
		27	NA	341	342	343	344	345	346	347	348	349	350	NA
		26	NA	340	339	338	337	336	335	334	333	332	331	NA
		25	NA	321	322	323	324	325	326	327	328	329	330	NA
		24	NA	320	319	318	317	316	315	314	313	312	311	NA
		23	NA	301	302	303	304	305	306	307	308	309	310	NA
		22	NA	300	299	298	297	296	295	294	293	292	291	NA
		21	NA	281	282	283	284	285	286	287	288	289	290	NA
		20	NA	280	279	278	277	276	275	274	273	272	271	NA
		19	NA	261	262	263	264	265	266	267	268	269	270	NA
		18	NA	260	259	258	257	256	255	254	253	252	251	NA
		17	NA	241	242	243	244	245	246	247	248	249	250	NA
		16	NA	240	239	238	237	236	235	234	233	232	231	NA
		15	NA	221	222	223	224	225	226	227	228	229	230	NA
		14	NA	220	219	218	217	216	215	214	213	212	211	NA
		13	NA	201	202	203	204	205	206	207	208	209	210	NA
		12	NA	200	199	198	197	196	195	194	193	192	191	NA
		11	NA	181	182	183	184	185	186	187	188	189	190	NA
		10	NA	180	179	178	177	176	175	174	173	172	171	NA
		9	NA	161	162	163	164	165	166	167	168	169	170	NA
		8	NA	160	159	158	157	156	155	154	153	152	151	NA
		7	NA	141	142	143	144	145	146	147	148	149	150	NA
		6	NA	140	139	138	137	136	135	134	133	132	131	NA
		5	NA	121	122	123	124	125	126	127	128	129	130	NA
		4	NA	120	119	118	117	116	115	114	113	112	111	NA
		3	NA	101	102	103	104	105	106	107	108	109	110	NA
		2	NA	NA	NA	NA	NA	NA	NA	NA	NA	NA	NA	NA
		1	NA	NA	NA	NA	NA	NA	NA	NA	NA	NA	NA	NA
		Field Row	1 2	3 4	5 6	7 8	9 10	11 12	13 14	15 16	17 18	19 20	21 22	23 24
		Plot Row	1	2	3	4	5	6	7	8	9	10	11	12

Ames 2023

The field coordinates were 42.014654°, -93.728797°. The previous crop was soybeans. Both inbreds and hybrids were planted on May 19, 2023. 3 rates of nitrogen (75, 150 and 225 lbs/acre) were applied with 32% UAN. Row spacing was 30”. In the map, hybrids are located between the sets of inbreds.

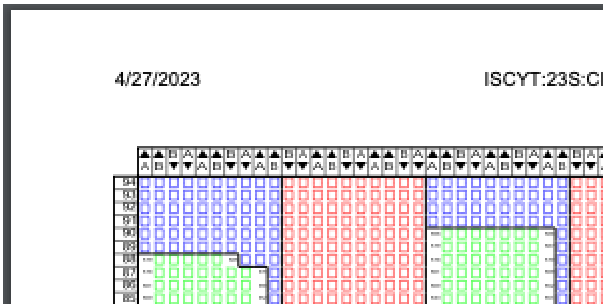

Hybrid HIPS field

Plots were 4 row plots, 20 feet center to center with 2.5 ft alleys. Hand harvest was completed on October 15, 2023. Mechanical harvest was completed on October 19, 2023.

Inbred HIPS field

Plots were 2 row plots, 10 feet center to center with 2.5 ft alleys. Hand harvest was completed between October 17 and November 16, 2023.

Crawfordsville 2023

The field coordinates were 41.194394°, -91.478950°. The previous crop was soybeans. Both inbreds and hybrids were planted on May 4, 2023. 3 rates of nitrogen (75, 150 and 225 lbs/acre) were applied with 32% UAN. Row spacing was 30”. In the map, hybrids are located between the sets of inbreds.

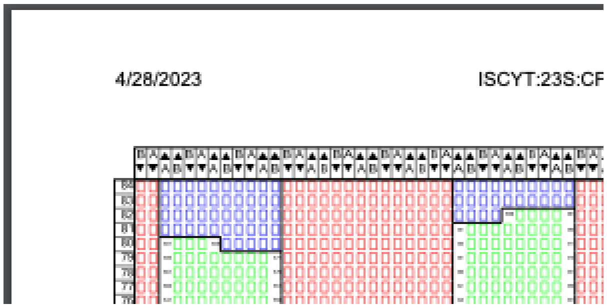

Hybrid HIPS field

Plots were 4 row plots, 20 feet center to center with 2.5 ft alleys. Hand harvest was completed on between September 29 and October 1, 2023. Mechanical harvest was completed on October 2, 2023.

Inbred HIPS field

Plots were 2 row plots, 10 feet center to center with 2.5 ft alleys. Hand harvest was completed on between September 29 and October 1, 2023.

Lincoln 2024

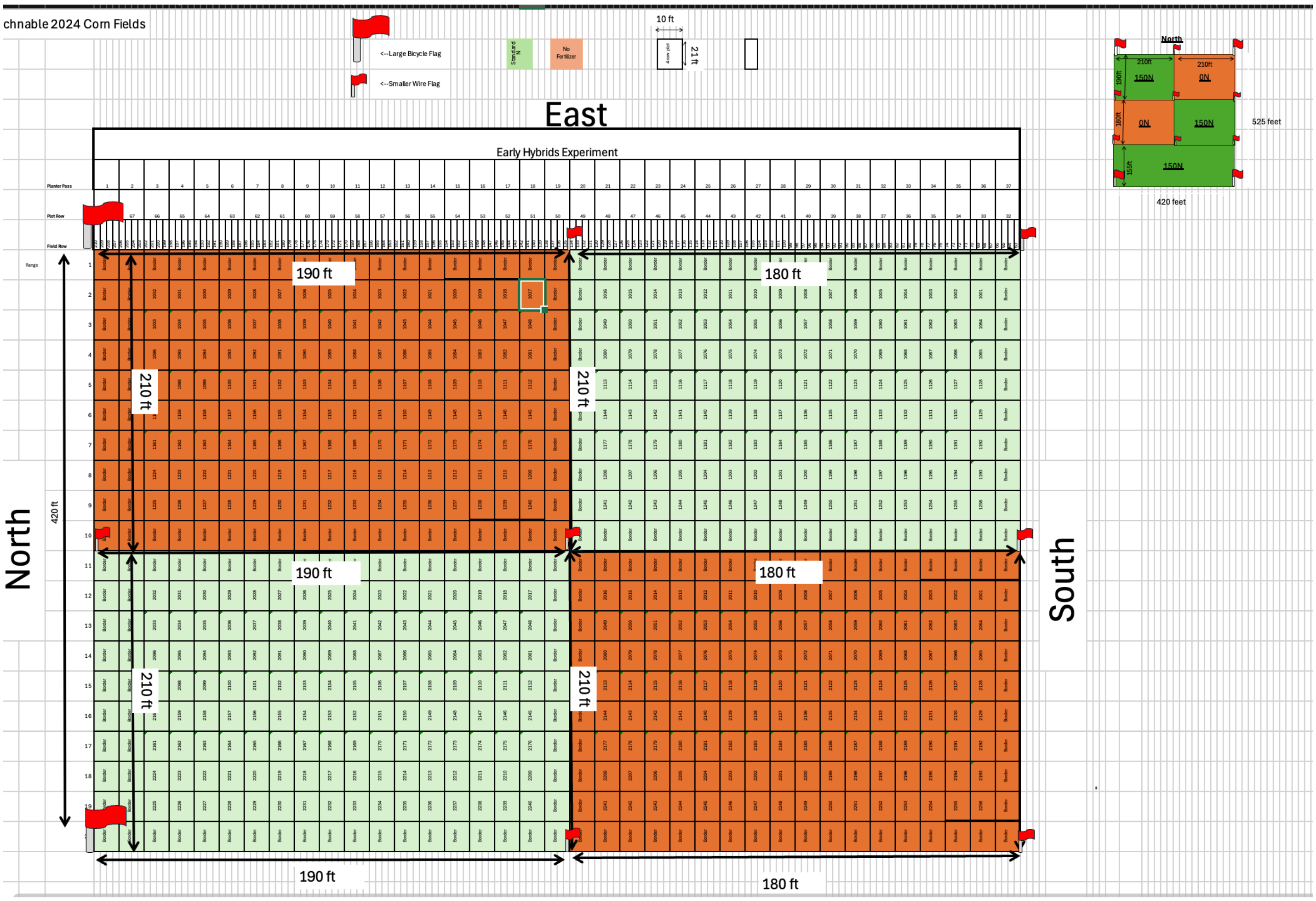

Location: University of Nebraska-Lincoln Havelock Research Farm
Field Block 2-15 Latitude: 40.863628 Longitude: -96.600976

Field Design: Seed was generated, packaged, and supplied by Lisa Coffey at ISU (lmcoffey@iastate.edu (mailto:lmcoffey@iastate.edu)) 4 total blocks (replications), 2 N+ and 2 N- N- Blocks - No supplemental nitrogen applied N+ Blocks - 150 lbs/acre of N applied 128 experimental plots/block (127 genotypes + 1 extra rep of [Std Q60 x M441D] as a planting check Four-row plots 30” row spacing 21 feet (center of alley to center of alley), 18 feet 6 inches (planted length)

Planting/Germination/Harvest Issues:
Planting: GPS disconnection caused a shift in Row 51 Ranges 1-10 towards the front of the field, Gap at Row 51, Range 10, filled with commercial seed, last experimental plot (originally Row 51, Range 2) in Range 1 (Border) Germination: 10 Plots with little/no germination: 1031, 1122, 1163, 1183, 1203,1238, 2064, 2094, 2216, 2255 Germination: 1 Plot - left two row did not germinate but right looked good: 2038 Harvest: Plots 2158 and 2163 combined together (mixed) during harvest, Yield data discarded

Field Management: Corn/Soybean crop rotation - soybeans in 2023 4/8/24 - 150 lbs/acre of liquid urea applied to N+ blocks 5/8/24 - 64 fl oz/acre Acuron + 32 fl oz/acre Liberty applied 5/15/24 - Field Planted 6/7/24 - Low/no germination plots filled with commercial hybrid seed (P1185AM) 6/10/24 - 48 fl oz/acre Acuron + NIS applied 6/18/24 - 6/20/24 - Field hand-weeded, canopy formed shortly after, no further weed issues 9/26/24 - Field Harvested UNL Farms (Josh Reznicek) with 2-row plot combine

Data Collection:
6/28/24 and 6/30/24 - Stand Count: Recorded hand-counts of the number of plants in the middle two rows of a plot in FieldBook 7/7/24 to 8/2/24 - Male Flowering: Recorded the date the plot reached the half-bloom stage (50% of plants have anthers emerging) in FieldBook 7/8/24 to 8/2/24 - Female Flowering: Recorded the date the plot reached the half-bloom stage (50% of plants have silks emerging) in FieldBook 7/31/24 - Chlorophyll Concentration: Collected using Apogee MC-100 Chlorophyll Concetration Meter, Fourth leaf from the top of the plant measured (flag leaf is 1st leaf) at roughly the midpoint of leaf (long) and the midpoint between midrib and leaf edge, Recorded the average of 3 plants per in FieldBook, Measured in absolute units [μmol m-2] 9/20/24 and 9/24/24 - Ear Height: Recorded the height of the ear of three plants per plot in FieldBook, Measured in feet using a ruled scale, Average of the three values calculated during data processing 9/20/24 and 9/24/24 - Plant Height: Recorded the height of three plants per plot in FieldBook, Measured from the ground to the tip of the tassel in feet using a ruled scale, Average of the three values calculated during data processing 9/20/24 and 9/24/24 - Lodging Percentage: If plots appear significantly lodged to the point of affecting combine data (>50% lodged), the lodging percentage was visually estimated and recorded in FieldBook. If the plots were only slightly lodged, “Stem Lodged” counts were divided by “Stand Count” to calculated a lodging percentage 9/20/24 and 9/24/24 - Stem Lodged: If plots had partial lodging (less than 50%), stem lodged plants in the middle two rows were counted and recorded, Stem lodging was defined as plants broken below the primary ear 9/26/24 - Yield: The middle two rows of each plot were harvested using a Zurn 160 Plot Combine by Josh Reznicek, Weight, Moisture, and Test Weight data was recorded by the combine software (Harvest Master’s Mirus Data Collection software, Yield was calculated from weight and moisture (plots weights adjusted to a standarized 15.5% moisture, divided by plot area (92.5ft2) and divided by 43560 to get lbs/acre, divided by 56 to get Bushels/acre

Weather Station: 6/5/24 - Weather station installed 6/5/24 to 6/27/24 - Wind direction vane not properly calibrated, wind direction during this time period is innacurate, DISCARDED 6/7/24 to 6/27/24 - Wind speed fan removed to replacing missing fan on Clearwater unit. Wind speed during this time period is innaccurate, DISCARDED

Severe Weather Events: 7/1/24 to 7/2/24 - Significant wind/rainfall event; Weather station showed a 24 hour precipitation total of 1.89” at 9am on 7/1/24, Weather station showed a 24 hour precipitation total of 6.26” at 7am on 7/2/24, Reports of 5”+ in Havelock during the night/early morning of 7/2/24 alone, Field flooded, flood damage occurred in low-lying plots, Water damaged plots indicated in the plot notes, Some lodging but largely recovered 7/31/24 - Significant wind/rain event, Weather Station showed a 24 hour precipitation total of 1.92” at 10am on 8/1/24, Reports of 75-90 mph winds, Significant lodging occurred and was noted in FieldBook

Other/Misc. Planting issue in Border rows (67 and 68) due to micommunication. Row 68 Ranges 19 and 20, and Row 67 Rang 20 were empty and filled with B104 inbred seed. This did not affect the experiemntal design Accuron should be applied at the optimal time to achieve desired weed control. The optimal time would be as weed seedling emerge and at appropriate soil moisture (4-5 days after rain) Low-yielding plots did not have enough grain (Minimum 4lbs) to measure moisture/test weight. These values were reported as 0s in the combine data and were replaced with NAs to avoid confusion

Clearwater 2024

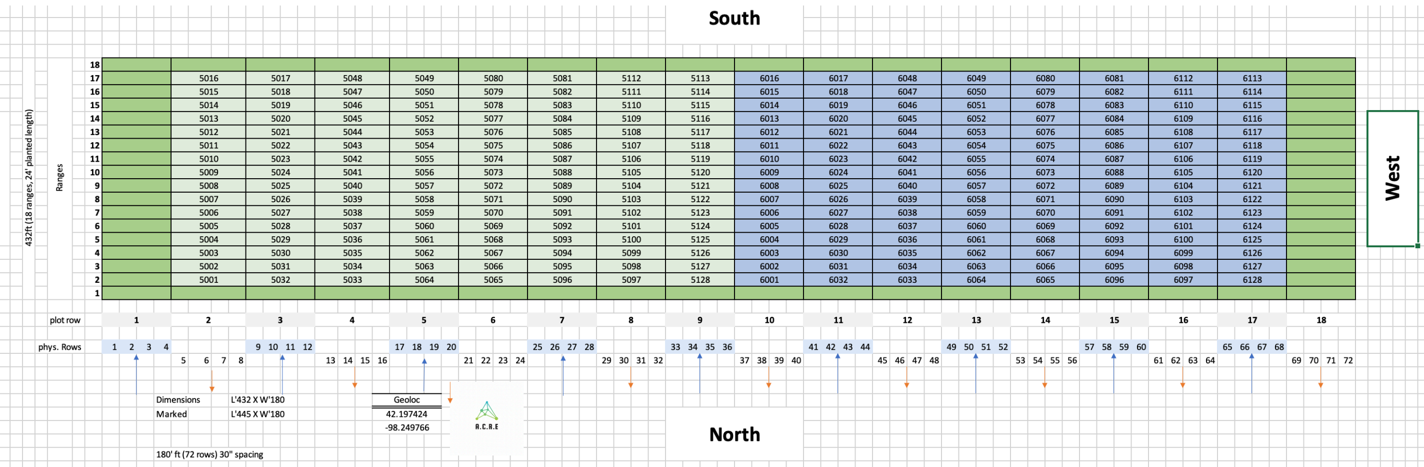

Location: Apex Custom Research Enterprises (A.C.R.E.) (nick@apexcustomresearchenterprises.com (mailto:nick@apexcustomresearchenterprises.com)) Clearwater, Nebraska Latitude: 42.204116 Longitude: - -98.246518

Field Design: Seed was generated, packaged, and supplied by Lisa Coffey at ISU (lmcoffey@iastate.edu (mailto:lmcoffey@iastate.edu)) 2 total blocks (replications) 128 experimental plots/block (127 genotypes + 1 extra rep of [Std Q60 x M441D] as a check) Four-row plots 30" row spacing 20 feet (center of alley to center of alley), 17 feet (planted length) Managed tp popcorn production standards, Pesticide, fertilizer, and irrigation outlined below

Planting Issues: Planting error in Row 17 Range 13, missed planting, gap at Row 17 Range 13, Plots shifted towards front of the field, last experimental plot (originally Row 17 Range 2) now in Range 1 (Border) 6 Plots with little/no germination: 5032, 5030, 5077, 6016, 6092, 6103

Field Management: Soybeans planted in 2023, Rye cover crop prior to 2024 season Total fertilizer application (lb/acre): N-P-K = 254-76-83 4/2/24 - Dry fertilizer application: 102.4 lbs/acre Potash (0-0-60, 61 lbs/acre K), 116.9 lbs/acre MESZ (12-40-0-10S-1ZN, 14 lb/acre N, 46 lb/acre P, 11.7 lb/acre S, 1.2 lb/acre ZN), 37.9 lb/acre KMAG (0-0-21.5-21.5-10.5, 8 lb/acre K, 8 lb/acre Mg, 4 lb/acre S) 4/20/14 - Burndown herbicide and N application: 16 oz/acre 2-4D LV6 AS 2.5G, 40 oz/acre Roundup Powermax 3, 51 lb/acre ammonium sulfate (21-0-0-24S, 11 lb/acre N, 12.2 lb/acre S) 5/3/24 - Field planted and dry fertilizer application: 24.8 lb/acre Potashh (0-0-60, 14 lb/acre K), 59.4 lb/acre MESZ (12-40-0-10S-1ZN, 9 lb/acre N, 30 lb/acre P, 7.5 lb/acre S, 0.75 lb/acre ZN) 5/18/24 - Herbicide and fertilizer application: 16 oz/acre 2-4D LV6 AS 2.5G, 4 oz/acre Arctic 3.2EC MB, 1 lb/acre, Harness Extra 6.0 Bulk, 20 gal/acre Liquid N (32-0-0, 59.4 lb/acre N), 2.6 gal/acre Thiosulfate (12-0-0-26S, 3 lb/acre N, 6.5 lb/acre S), 6/12/24 - Herbicide and fertilizer application: 1.8 oz/acre Accent Q, 1.5 lb/acre Atrazine 90DF Winfield, 3 oz/acre Laudis, 51 lb/acre ammonium sulfate (21-0-0-24S, 11 lb/acre N, 12.2 lb/acre S) 6/18/24 - Field cultivated, fertigation: 10.6 gal/acre Advancedf Liquid N (28-0-0-5S, 32.2 lb/acre N, 5.8 lb/acre S) 6/28/2024 - Fertigation: 11.4 gal/acre Advancedf Liquid N (28-0-0-5S, 34.2 lb/acre N, 6.1 lb/acre S) 7/11/2024 - Fertigation: 11.3 gal/acre Advancedf Liquid N (28-0-0-5S, 33.8 lb/acre N, 6 lb/acre S) 7/16/2024 - Fertigation: 10.6 gal/acre Advancedf Liquid N (28-0-0-5S, 31.7 lb/acre N, 5.7 lb/acre S) 8/8/2024 - Fertigation: 4.9 gal/acre Advancedf Liquid N (28-0-0-5S, 14.6 lb/acre N, 2.6 lb/acre S) 10/9/24 and 10/10/24 - Harvested

Irrigation: Approximately 16" of supplemental irrigation from July-September (~1.5" per week), Center-pivot irrigation system

Data Collection:

Populations: Recorded the number of plants in the middle two rows of the plot, dived that number by 2 to get an average stand per row, and this multiplied this value by 1000 (1 row is ~1/1000 acre) to get populations in

plants/acre Standability and Std %: Visually rated plots from 0-10 - SEE SCALE BELOW), The std% is the square of the standability rating, 0 = no lodging (all plants standing), 2 = 4% lodged, 3 = 9% lodged, 4 = 16% lodged, 5 = 25% lodged, 6 = 36% lodged, 7 = 49% lodged, 8 = 64% lodged, 9 = 81% lodged, 10 = 100% lodged (all plants lodged/broken/on ground) 7/8/24 to 7/23/24 - Male Flowering: Recored the date the plot reached the half-bloom stage (50% of plants have anthers emerging) 7/10/24 to 7/24/24 - Female Flowering: Recorded the date the plot reached the half-bloom stage (50% of plants have silks emerging) 10/9/24 to 10/10/24 - Yield: The middle two rows of each plot were harvested with a plot combine by Nick Hoffman of A.C.R.E., Weight, Moisture and yield data was recorded by the combine software

Weather Station: 6/10/24 - Weather station installed

Severe Weather Events: One small wind event which did not do much damage, some corn borer pressure but not excessive

Other/Misc.: Wildlife (raccoons) predated certain plots, damaging plants, removing ears and reducing yield. These plots are indicated with a “wildlife damage” note in the Notes column, Damage was first noticed after flowering, Nick Hoffman orignally thought the damage was limited to 1-2 hybrids in both reps, Damage ended up being more extensive with Nick observing more damage to “white multicolered hybrids”, Trial was surrounded by popcorn, Raccoons appeared to much prefer field corn, Particulary palatable hybrids targeted. Some plots were nearly 100% damaged while adjacent plots appeared untouched; Nick describes the trial’s proximity to a riparian area as a contributing factor. He was unsure of the best method of controlling raccoons or what level of damage warranted further action and none was taken. Nick was disappoitned by this and did not forsee this issue as this was his first year with the business/field. Minor corn borer pressure, not excessive

Additional Data Available

Upon request, the following data is available:

	Location										
Phenotype	Scottsbluff 2022	North Platte 2022	Lincoln 2022	Missouri Valley 2022	Ames 2022	Crawfordsville 2022	North Platte 2023	Lincoln 2023	Missouri Valley 2023	Ames 2023	Crawfordsville 2023
Ear-level phenotypes included in data set at plot-level	x	x	x	x	x	x	x	x	x	x	x
Replicate NIR measurements (3 per plot)	x	x	x	x							
Leaf length (2 plants per plot)			x								
Leaf width (2 plants per plot)			x								
Plant-level plant height measurements (2 plants per plot)			x					x			
Ear drop number		x									
Row-level stand counts		x		x	x	x					
Tassel tip height	x		x					x			
Kernel striping	x	x	x	x							
Occurrence of sweet corn kernels					x	x				x	x
Shoot lodging percent		x		x	x	x			x	x	x
Root lodging percent		x		x	x	x			x	x	x
Plot-level latitude and longitude				x	x	x			x	x	x

Appendix

Documentation for Additional Data Available

Leaf Length

This is the length in centimeters of the ear leaf from the stalk to leaf tip of one plant from the plot. It is abbreviated as **'leaf_len1'**. This information for the Lincoln location was taken from the sheet 'Combined Dataset' in the file 'Summary of Lincoln Hybrid HIPS 2022 Data.xlsx'.

Leaf Width

This is the width in centimeters of the midpoint of the ear leaf of one plant from the plot. It is abbreviated as **'leafWidth1'**. This information for the Lincoln location was taken from the sheet 'Combined Dataset' in the file 'Summary of Lincoln Hybrid HIPS 2022 Data.xlsx'.

Ear

This is the order the ears were phenotyped. It is abbreviated in the code as 'earNum', but does not appear in the final dataset as the ear data was transformed to have four columns each for ear width, kernel fill length, kernel row number, kernels per row, ear weight, seed color, total kernel count, cob length, cob width, cob weight, and 100 kernel weight. These variables with the suffix '1' correspond to ears with an ear number of 1 in the dataset, and this convention is maintained for the variable suffixes 2-4. All ears with an ear number greater than 4 were dropped to create a balanced dataset.

Ear Weight

This is the weight of one ear from the plot in grams prior to shelling (i.e. with the kernels attached to the cob). This corresponds to the ear with an ear number of 1 from the original dataset. It is abbreviated as **'earWt1'**. This information for Lincoln hybrids, Missouri Valley hybrids and inbreds, and some of the North Platte location, was taken from the file '2022_Hybrid HIPS - Post Harvest Data - Prototype File.csv'. Information for the remaining ears from North Platte and the Scottsbluff hybrids were taken from the file 'NP-SB_2022'.

Ear Drop Number

This is the number of ears that fell to the ground in the middle two rows of the plot for hybrids. It is abbreviated as **'earDropNum'**. This information for North Platte was taken from the sheets 'No Irr Data', 'Reduced Irr Data', and 'Full Data' in the file '2022_Schnable_HIPS_data_v4.xlsx'.

Stand Count 1

This is the number of standing plants in one row of the plot. It is abbreviated as **'standCt1'**. This information for Missouri Valley was taken from the sheets 'RawData (4-Row)' and 'RawData (2-Row)' for hybrids and inbreds, respectively, in the file 'YTMC_Lisa_Plot_Coordinates_v4.xlsx'. This information for North Platte was taken from the sheets 'No Irr Data', 'Reduced Irr Data', and 'Full Data' in the file '2022_Schnable_HIPS_data_v4.xlsx'.

Stand Count 2

This is the number of standing plants in a second row of the plot. It is abbreviated as **‘standCt2’**. This information for Missouri Valley was taken from the sheets ‘RawData (4-Row)’ and ‘RawData (2-Row)’ for hybrids and inbreds, respectively, in the file ‘YTMC_Lisa_Plot_Coordinates_v4.xlsx’. This information for North Platte was taken from the sheets ‘No Irr Data’, ‘Reduced Irr Data’, and ‘Full Data’ in the file ‘2022_Schnable_HIPS_data_v4.xlsx’.

Historical Flowering Time

This is the historical flowering time, in days, of the inbred and was used to for blocking the inbreds. It is abbreviated as **‘histFT’**. This information was taken from the sheet ‘RawData (2-Row)’ in the file ‘YTMC_Lisa_Plot_Coordinates_v4.xlsx’.

Historical Plant Height

This is the historical plant height, in centimeters, of the inbred, and was used for blocking the inbreds. It is abbreviated as **‘histPlantHt’**. This information was taken from the sheet ‘RawData (2-Row)’ in the file ‘YTMC_Lisa_Plot_Coordinates_v4.xlsx’.

Inbred Block

This is the block the inbred was placed in based on historical plant height and days to flowering. It is abbreviated as **‘block’**. This information was taken from the sheet ‘RawData (2-Row)’ in the file ‘YTMC_Lisa_Plot_Coordinates_v4.xlsx’.

Population

This is the genotypic population the plants grown in the plot are from, either the Hybrid HIPS population, abbreviated as ‘Hybrid’, or the Inbred HIPS population, also known as the SAM population, abbreviated as ‘Inbred’. It is abbreviated as **‘population’**.

Ear Length - Pre-Shelling

This is the mean length of the ear prior to shelling (i.e. with the kernels on the ear) of one ear from the plot. It is abbreviated as **‘earLen’**. This information for the Ames and Crawfordsville locations was taken from the files in the folder ‘3 Ear Traits Station’ and converted from millimeters to centimeters. For these two locations, when the ear had severe bending, a string was used to measure the length and this is denoted in the notes field. This data was not collected at the North Platte, Scottsbluff, Lincoln, and Missouri Valley locations.

Tassel Tip Height

This is the height in centimeters of the plant, including the tassel, of one plant from the plot (in the case of Scottsbluff) or the average of this measurement for two plants from the plot in Lincoln. All measurements from plants noted as stunted or without a silk were marked as missing data prior to calculation. It is abbreviated as **‘tasselTipHt’**. This information for the Lincoln location hybrids was taken from the sheet ‘Combined Dataset’ in the file ‘Summary of Lincoln Hybrid HIPS 2022 Data.xlsx’. For Scottsbluff hybrids, this data was taken from the sheet ‘Hybrid_height data’ from the file ‘Corn_data_Scottsbluff-2022_rk_11.11.2022’ and converted from inches. Plot 1322 at the Scottsbluff location was dropped due to having a value more than 114 centimeters (roughly 3.7 feet) greater than all other observations.

KernelStriping

This is whether or not the kernels from the plot exhibited striping. It is abbreviated as **‘kernelStriping’**. This information for hybrids and Missouri Valley inbreds was taken from the file ‘plotleveleardata_v2.csv’. This information was not collected for the Ames and Crawfordsville locations.

Percent Shoot Lodge

This is the percent of plants that lodged due to stalk breakage in the middle two rows of the plot for hybrids and in the whole plot for inbreds. It is abbreviated as **‘pctStalkLodge’.** This information for Missouri Valley, Crawfordsville, and Ames was taken from the sheets ‘RawData (4-Row)’ and ‘RawData (2-Row)’ for hybrids and inbreds, respectively, in the file ‘YTMC_Lisa_Plot_Coordinates_v4.xlsx’. This information for North Platte was taken from the sheets ‘No Irr Data’, ‘Reduced Irr Data’, and ‘Full Data’ in the file ‘2022_Schnable_HIPS_data_v4.xlsx’.

Percent Root Lodge

This is the percent of plants that lodged due to insufficient roots in the middle two rows of the plot for hybrids and in the whole plot for inbreds. It is abbreviated as **‘pctStalkLodge’.** This information for Missouri Valley, Crawfordsville, and Ames was taken from the sheets ‘RawData (4-Row)’ and ‘RawData (2-Row)’ for hybrids and inbreds, respectively, in the file ‘YTMC_Lisa_Plot_Coordinates_v4.xlsx’. This information for North Platte was taken from the sheets ‘No Irr Data’, ‘Reduced Irr Data’, and ‘Full Data’ in the file ‘2022_Schnable_HIPS_data_v4.xlsx’.

latitude

This is the latitude of the plot. This information for Missouri Valley, Crawfordsville, and Ames was taken from the sheets ‘RawData (4-Row)’ and ‘RawData (2-Row)’ for hybrids and inbreds, respectively, in the file ‘YTMC_Lisa_Plot_Coordinates_v4.xlsx’.

longitude

This is the longitude of the plot. This information for Missouri Valley, Crawfordsville, and Ames was taken from the sheets ‘RawData (4-Row)’ and ‘RawData (2-Row)’ for hybrids and inbreds, respectively, in the file ‘YTMC_Lisa_Plot_Coordinates_v4.xlsx’.
